# Optimization of Selectivity and Pharmacokinetic Properties of Salt-Inducible Kinase Inhibitors that Led to the Discovery of Pan-SIK Inhibitor GLPG3312

**DOI:** 10.1021/acs.jmedchem.3c01428

**Published:** 2023-12-26

**Authors:** Taouès Temal-Laib, Christophe Peixoto, Nicolas Desroy, Elsa De Lemos, Florence Bonnaterre, Natacha Bienvenu, Olivier Picolet, Eric Sartori, Denis Bucher, Miriam López-Ramos, Carlos Roca Magadán, Wendy Laenen, Thomas Flower, Patrick Mollat, Olivier Bugaud, Robert Touitou, Anna Pereira Fernandes, Stephanie Lavazais, Alain Monjardet, Monica Borgonovi, Romain Gosmini, Reginald Brys, David Amantini, Steve De Vos, Martin Andrews

**Affiliations:** † Galapagos SASU, 102 Avenue Gaston Roussel, 93230 Romainville, France; ‡ Galapagos NV, Generaal De Wittelaan L11, A3, 2800 Mechelen, Belgium

## Abstract

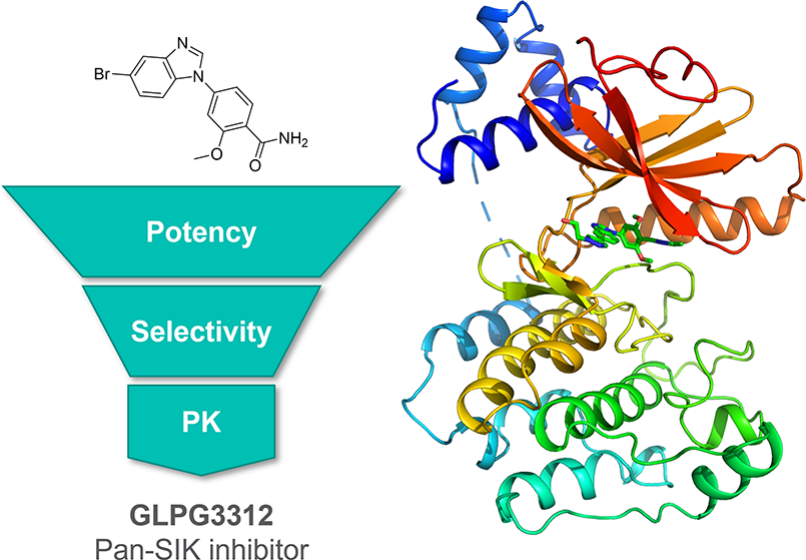

Salt-inducible kinases (SIKs) SIK1, SIK2, and SIK3 are serine/threonine kinases and form a subfamily of the protein kinase AMP-activated protein kinase (AMPK) family. Inhibition of SIKs in stimulated innate immune cells and mouse models has been associated with a dual mechanism of action consisting of a reduction of pro-inflammatory cytokines and an increase of immunoregulatory cytokine production, suggesting a therapeutic potential for inflammatory diseases. Following a high-throughput screening campaign, subsequent hit to lead optimization through synthesis, structure–activity relationship, kinome selectivity, and pharmacokinetic investigations led to the discovery of clinical candidate GLPG3312 (compound **28**), a potent and selective pan-SIK inhibitor (IC_50_: 2.0 nM for SIK1, 0.7 nM for SIK2, and 0.6 nM for SIK3). Characterization of the first human SIK3 crystal structure provided an understanding of the binding mode and kinome selectivity of the chemical series. GLPG3312 demonstrated both anti-inflammatory and immunoregulatory activities *in vitro* in human primary myeloid cells and *in vivo* in mouse models.

## Introduction

**Figure 1:**
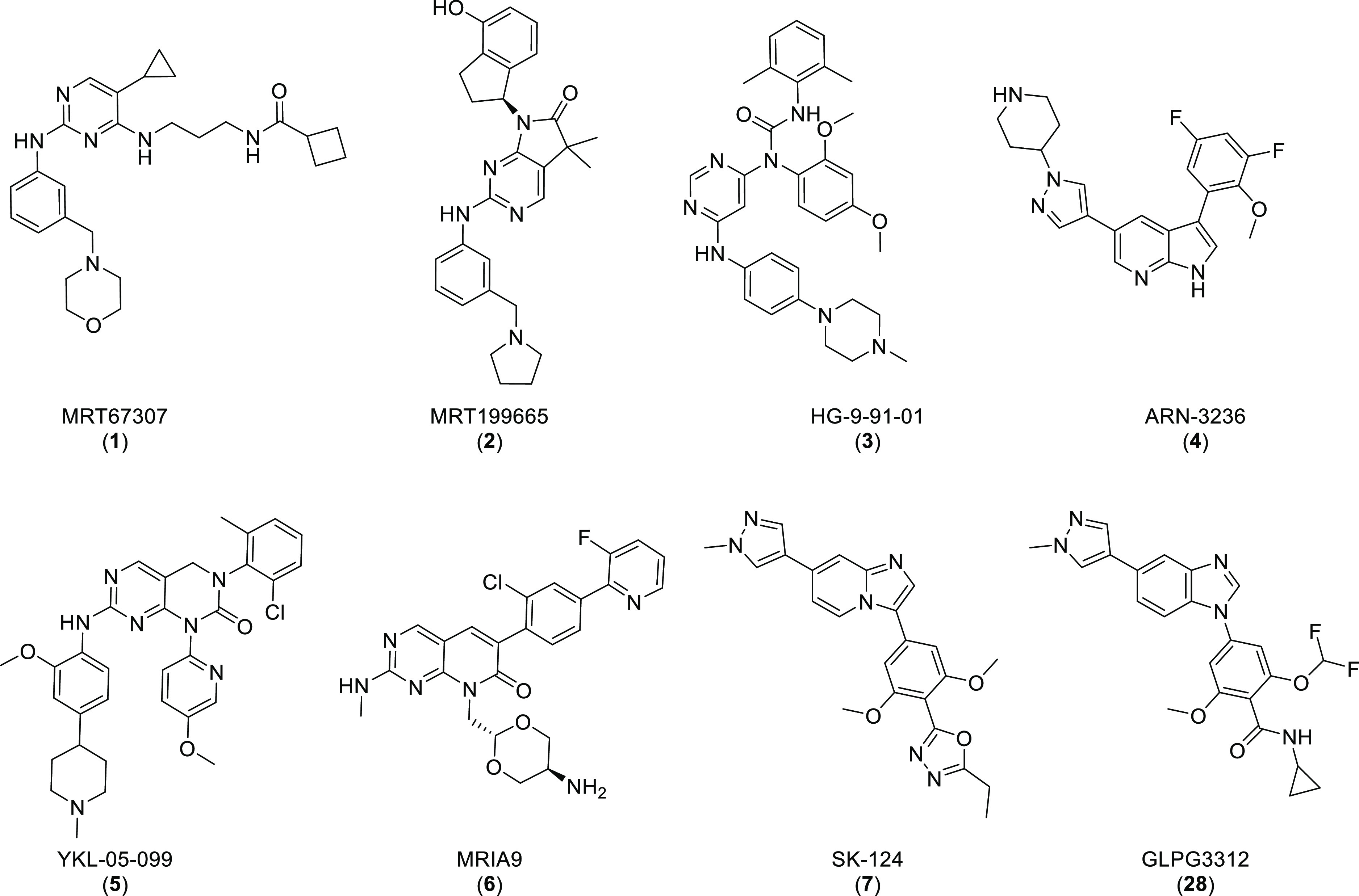
Structures of SIK inhibitors described in the literature and of GLPG3312 (**28**).

The salt-inducible kinase (SIK) family comprises three isoforms (SIK1, SIK2, and SIK3) that belong to the AMP-activated protein kinase (AMPK) family of serine/threonine protein kinases.^1^ SIKs have been implicated in the regulation of several physiological processes, including circadian rhythms, bone formation, skin pigmentation, metabolism, and modulation of inflammatory cytokine production. Consequently, the potential of SIK inhibitors has drawn interest in various therapeutic areas.^2^ Several SIK inhibitors such as compounds **1**–**7** (Figure 1) have been reported and used to investigate SIK biology *in vitro* and *in vivo*. As frequently observed with kinase inhibitors, compounds **1**–**7** also inhibit numerous other kinases that may limit their potential therapeutic applications (Table 1).

**Table 1:** Potency and Selectivity of SIK Inhibitors

Compound	SIK1 IC_50_, nM	SIK2 IC_50_, nM	SIK3 IC_50_, nM	other protein kinases (IC_50_, nM; <1000 nM)	kinase panel size; kinases above inhibition cutoff
MRT67307 (**1**)^6^	250	67	430	TBK1 (19), MARK1 (27), MARK3 (36), MARK4 (41), MARK2 (52), IKKε (160), NUAK1 (230), AMPKα1/α2 (810), MELK (900)	108; >80% inhibition at 1 μM: Aurora B, JAK2, MLK1, MLK3
MRT199665 (**2**)^6^	110	12	43	MARK1 (2), MARK2 (2), MARK4 (2), MARK3 (3), NUAK1 (3), AMPKα1/α2 (10), MELK (29), NUAK2 (120)	108; >80% inhibition at 1 μM: IR, JAK2, MLK1, MLK3
HG-9-91-01 (**3**)^6^	0.9	0.6	9.6	NUAK2 (145)	108; >80% inhibition at 0.1 μM: Src, YES1, EPH-A2, EPH-A4
ARN-3236 (**4**)^8,11,12^	21.6	<1	6.6		74; >80% inhibition at 0.5 μM: JAK2, LCK, NUAK2, SRPK1, VEGFR2
YKL-05-099 (**5**)^9^	10 (binding)	40	30 (binding)		140; >80% inhibition at 1 μM: RIPK2, ABL, Src, DDR2, MAP4K3, P38a, BTK, EPH-B3, YES1, EPH-B4, EPH-B2, EPH-A4, Lck, BRK
MRIA9 (**6**)^13^	55	48	22	PAK2 (41), PAK3 (140), KHS1 (210), NLK (250), PAK1 (580), MAP2K4 (830)	443
SK-124 (**7**)^14^	6.5	0.4	1.2	IC_50_ <100 nM: PDGFRα (15), CSK (54), TNIK (56), TBK1 (67), IKKε (70), ABL1 (72)	300

In healthy individuals, a tightly regulated balance of pro- and anti-inflammatory pathways maintains immune homeostasis. However, in inflammatory diseases, imbalances in pro-inflammatory versus immunoregulatory processes cause chronic inflammation, which, if left untreated, leads to tissue damage in the body. Despite a broad range of treatment options in inflammatory diseases like rheumatoid arthritis (RA) and inflammatory bowel disease (IBD), many patients do not achieve full remission, highlighting a significant unmet medical need.^3–5^ Myeloid cells play key roles during the initiation, propagation, and resolution of inflammation. The SIKs control gene regulation and act as a molecular switch, and inhibition has been shown to reprogram myeloid cell types to an immunoregulatory phenotype.^6–9^ The evaluation of pharmacological pan-SIK kinase inhibitors **1**–**4** (Figure 1) in *in vitro* macrophage and dendritic cell models stimulated with Toll-like receptor (TLR) 2 or TLR4 ligands has been shown to reduce the release of tumor necrosis factor (TNF) α and interleukin (IL) 12 and to stimulate the production of anti-inflammatory mediators, such as IL-10.^6,8,9^ Consistent with *in vitro* observations, intraperitoneal dosing of compound **5** to mice prior to lipopolysaccharide (LPS) challenge was found to lead to a reduced abundance of TNFα and increased IL-10 levels in the serum of mice relative to controls. More recently, intraperitoneal administration of HG-9-91-01 (**3**) in mouse models of colitis led to an improvement of the disease score coupled with a decrease of TNFα and IL-12, and an increase of IL-10 in colonic tissues.^10^

Altogether, this body of evidence suggests that selective SIK inhibition is an attractive therapeutic approach for the treatment of inflammatory diseases such as IBD. We embarked on a drug discovery program with the goal to identify a potent pan-SIK inhibitor with excellent kinome selectivity and suitable pharmacokinetic and ADMET properties for *in vivo* evaluation after oral dosing and for further preclinical development.

Here, we report the identification of a new chemotype for SIK inhibition, the first X-ray crystal structure of SIK3 that enabled an understanding of the binding mode and selectivity of the new chemotype, and the optimization of kinase selectivity and pharmacokinetic properties that led to pan-SIK inhibitor clinical candidate GLPG3312.

## Results and Discussion

### Hit Identification

A high-throughput screening (HTS) campaign of approximately 42,000 compounds from the Galapagos kinase-focused internal library was conducted using the ADP-Glo assay^15^ and using AMARA peptide as the phosphorylation substrate. Compounds found to be potent in inhibiting SIK3 had their IC_50_s further determined for SIK1 and SIK2. From this screening, compound **8** from 4-(5-substituted-benzimidazol-1-yl)-2-methoxy-benzamide chemical series was identified as a hit compound with IC_50_ values of 424, 300, and 188 nM against SIK1, SIK2, and SIK3, respectively (Table 2). Compound **8** had moderate molecular weight (346 Da) and lipophilicity (CLogP = 3.25) and displayed bromine and amide moieties readily amenable to modifications, making the molecule a suitable hit for further optimization.

**Table 2:**
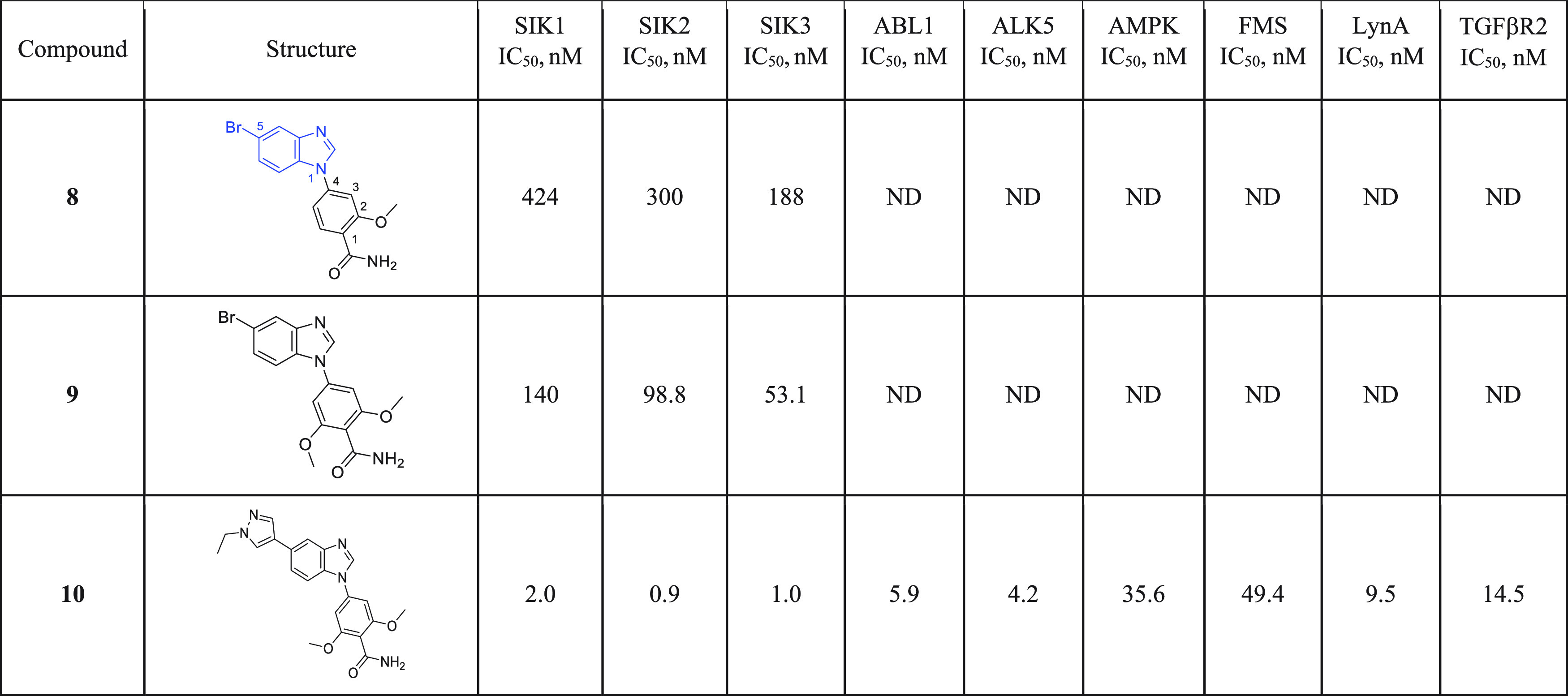
Hit Identification and Preliminary SAR Optimization–Activity of Compounds **8**, **9**, and **10**

### SAR Optimization

Starting from compound **8** that displayed moderate inhibitory activity, the impact of adding substituents and the expansion of existing substitution vectors was explored to increase potency while monitoring kinase selectivity. Initial SAR investigation showed that the introduction of a second methoxy group to the phenyl ring of **8** resulted in a 3-fold gain of potency against SIK1, SIK2, and SIK3 for **9** (Table 2). More importantly, replacement of the bromine at position 5 of the benzimidazole core of **9** with an *N*-ethyl pyrazole moiety resulted in a more than 50-fold boost of potency, affording nanomolar pan-SIK inhibitor **10** (Table 2). The selectivity profile of the very potent pan-SIK inhibitor **10** was evaluated against a panel of kinases, including ABL1, ALK5, AMPK, FMS, LynA, and TGFβR2, selected both for their homology with SIK and their undesirable pharmacological inhibition.^16–18^ Compound **10** was found to inhibit ABL1, ALK5, AMPK, FMS, LynA, and TGFβR2 kinases with an IC_50_ value below 50 nM (Table 2). Further optimization of the structure–activity relationship aimed to improve the selectivity against these kinases while maintaining potency against SIKs.

We theorized that the carboxamide group possibly interacts in the phosphate binding region of multiple kinases, and such moiety can serve as an off-target pharmacophore, hence replacement and alkylation of the carboxamide group were investigated. Replacement of this group by a methyl ester (**11**) or a carboxylic acid (**13**) led to a 10-fold or more drop of potency against SIKs, suggesting a contribution of the hydrogen bond donor group of the amide to the potency of compound **10** on SIKs (Table 3). Substitution of the carboxamide by a hydroxymethyl group containing such a hydrogen bond-donating group (**12**) only showed a 2- to 4-fold decrease of activity. The SAR was found to be distinct between off-target kinases; for example, replacement of the carboxamide group (**10**) by a methyl ester (**11**) led to a more than 100-fold decrease in activity against AMPK but had no impact on activity against FMS. In contrast, replacement of the carboxamide group (**10**) by a carboxylic acid had a minor impact on the activity against AMPK but led to more than a 40-fold drop of activity against FMS. Compound **12** had an interesting profile, retaining potent activity below 10 nM on SIKs and improved selectivity against ALK5, AMPK, LynA, and TGFβR2. As further selectivity improvement was desired against ABL1 and FMS, alkylation of the amide group was investigated next to assess whether off-target activity could be decreased by a steric clash in this region.

**Table 3:**
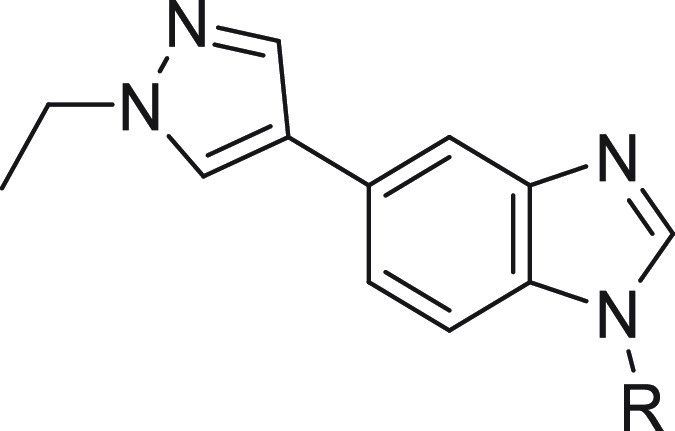

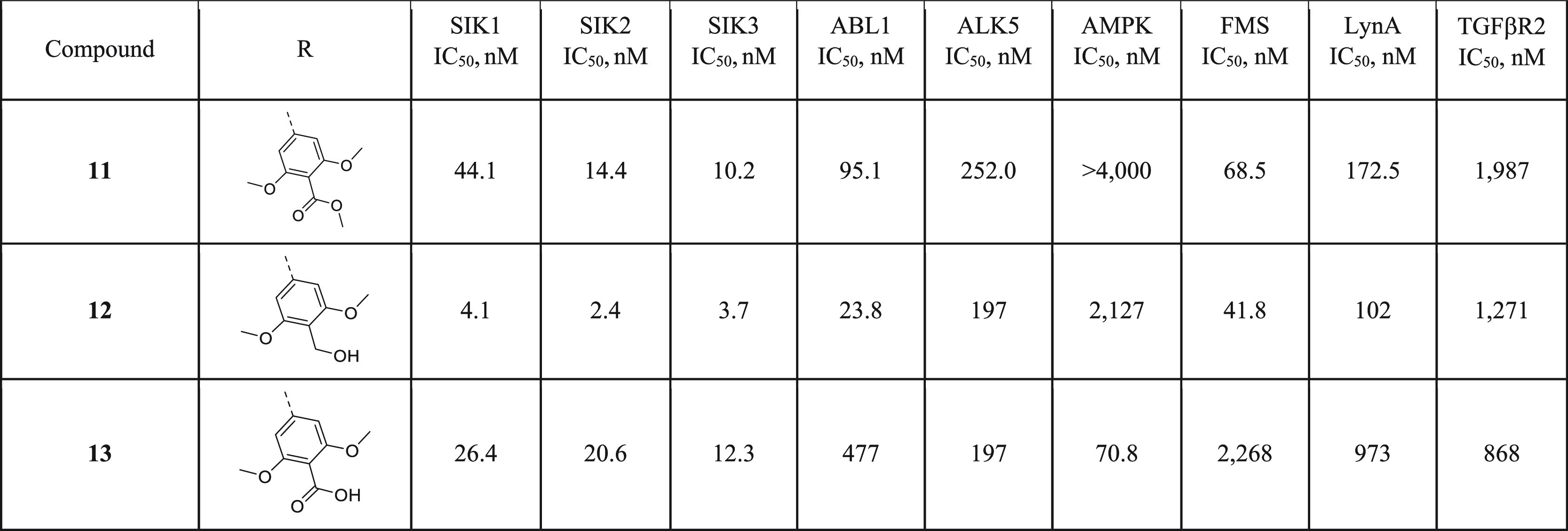
Analogues with Replacement of the Carboxamide Group

Secondary and tertiary amides of compound **10** were prepared and evaluated (Table 4). Introduction of a small substituent such as methyl (**14**) or ethyl (**15**) led to a 3- to 12-fold loss of potency against SIKs and a more than 20-fold drop of activity against ALK5, AMPK, and TGFβR2. In contrast, bulkier substituents, such as in trifluoroethyl (**16**) and cyclopropyl (**17**), retained similar potency against SIK3 to **10** and slightly decreased activity on SIK1 and SIK2. Trifluoroethyl (**16**) and cyclopropyl (**17**) analogues also led to further improvement of selectivity against off-target kinases ABL1, ALK5, and LynA compared with **10**, **14**, and **15**. A further increase of the size of the substituent with a *tert*-butyl group (**18**) caused a major loss of activity against SIKs likely due to a steric clash and resulted in micromolar activity against the three isoforms. Similarly, additional alkylation on the carboxamide with a methyl group in **19** led to a more than 100-fold drop of potency against SIKs, which could be due to a steric clash or loss of the hydrogen bond donor capacity of the amide. Modification of the methoxy groups was also investigated as an option to impact potency and off-target selectivity. Interestingly, replacing one of the methoxy groups on the phenyl ring by a difluoromethoxy group in compound **20** gave a 3-fold gain of potency against SIKs compared with **15** and decreased activity against the six off-target kinases, in particular AMPK with an IC_50_ superior to 4 μM. Compound **20** displayed similarly low nanomolar activity against SIKs as **10** and an IC_50_ above 150 nM for all the off-target kinases identified for **10**. As depicted in the next section, the difluoromethoxy group can make a hydrogen bond interaction in SIKs and lead to a steric clash in other kinases such as AMPK, leading to improvement of on-target potency and off-target selectivity. In summary, exploration of the SAR of the benzamide moiety led to the identification of trifluoroethyl (compound **16**) and cyclopropyl (compound **17**) moieties as amide substituents, providing high potency on SIKs and improved selectivity against off-targets compared to unsubstituted amide. Introduction of a second methoxy group on the phenyl ring in the ortho position of the carboxamide moiety increased potency on SIKs, and replacement of one of the methoxy groups by a difluoromethoxy group led to a gain of potency on SIKs and enhanced selectivity against off-targets (compound **20**).

**Table 4:**
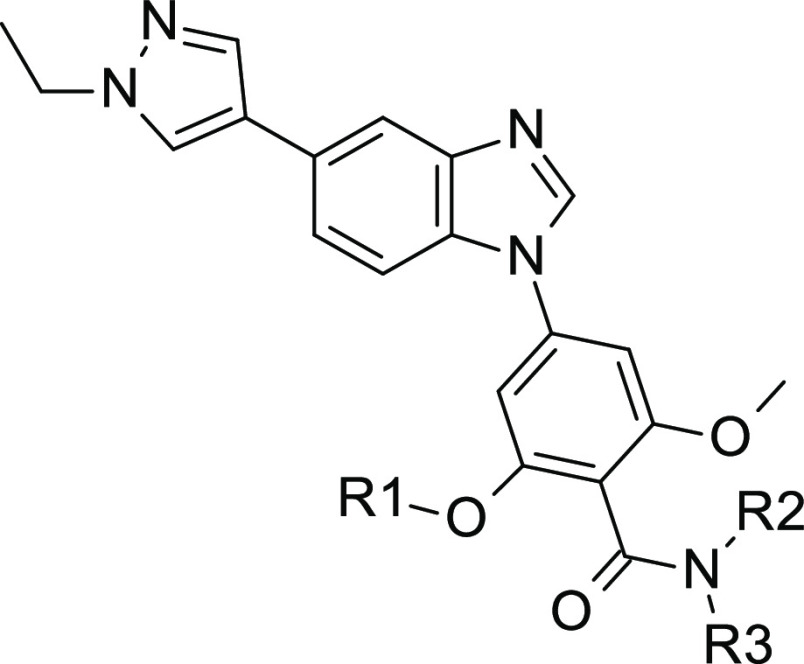
Exploration of Carboxamide Alkylation

Compound	R1	R2	R3	SIK1 IC_50_, nM	SIK2 IC_50_, nM	SIK3 IC_50_, nM	ABL1 IC_50_, nM	ALK5 IC_50_, nM	AMPK IC_50_, nM	FMS IC_50_, nM	LynA IC_50_, nM	TGFβR2 IC_50_, nM
**10**	Me	H	H	2	0.9	1.0	5.9	4.2	35.6	49.4	9.5	14.5
**14**	Me	Me	H	25.3	10.5	5.0	36.0	469	772	128.5	47.3	1363
**15**	Me	Et	H	20.2	7.9	2.9	52.5	550.6	658.6	84.4	75.3	703.2
**16**	Me	CH_2_–CF_3_	H	10.8	3.3	1.4	238.6	1550	582.2	40.5	363.0	771.8
**17**	Me	CyPr	H	8.1	2.5	1.3	133.0	1017	263.2	72.9	164.9	1044
**18**	Me	*t*Bu	H	>3987	>3920	>4000	ND	ND	ND	ND	ND	ND
**19**	Me	CyPr	Me	762.5	597.0	163.7	ND	ND	>4000	ND	ND	ND
**20**	CHF_2_	Et	H	5.8	2.3	1.0	172.4	1207	>4000	193.6	380	1327

The SAR around the pyrazole group was also explored to understand the impact on potency and selectivity using **15** as the basis, and the results are shown in Table 5. Shortening of the ethyl group to a methyl group as in **21** retained similar activity against SIKs and selectivity against off-targets as **15**. Introduction of a hydrogen bond-donating group in hydroxyethyl (**22**) and methyl carboxamide (**23**) derivatives also retained activity against SIKs but also increased selectivity against ABL1, ALK5, and TGFβR2. Other substitutions such as cyanomethyl (**24**), methoxyethyl (**25**), and 4-tetrahydropyranyl (**26**) did not bring further improvement on potency against SIKs or on selectivity against off-targets. Overall, substitution of the pyrazole ring with alkyl groups bearing hydrogen bond-accepting and hydrogen bond-donating groups as in compounds **22**–**26** was found to have a limited impact on SIK activity consistent with a moiety pointing toward the solvent region as described in the next section. The improvement of selectivity against ABL1, ALK5, and TGFβR2 off-target kinases may result from a different environment and less flexibility in this region in these kinases compared to SIKs.

**Table 5:**
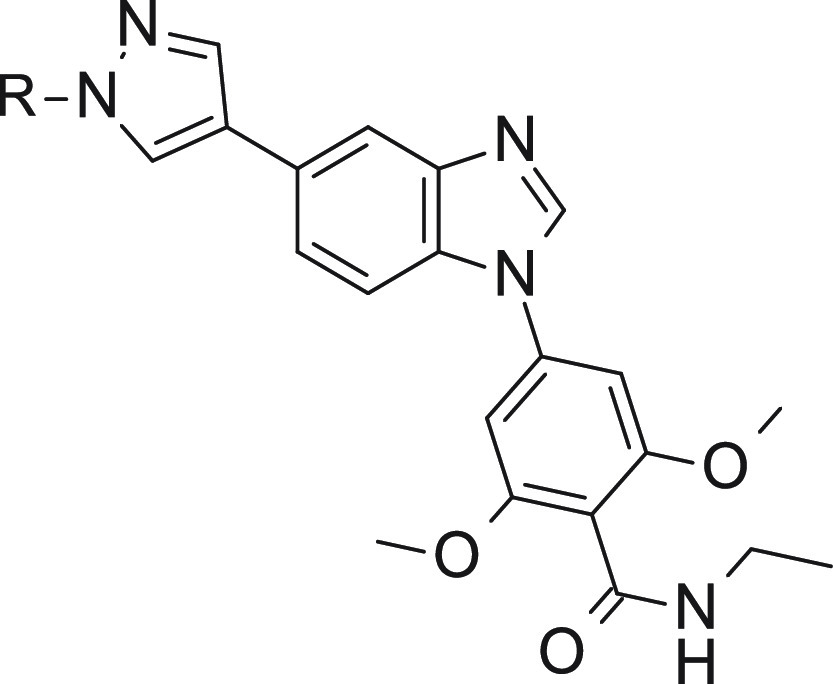

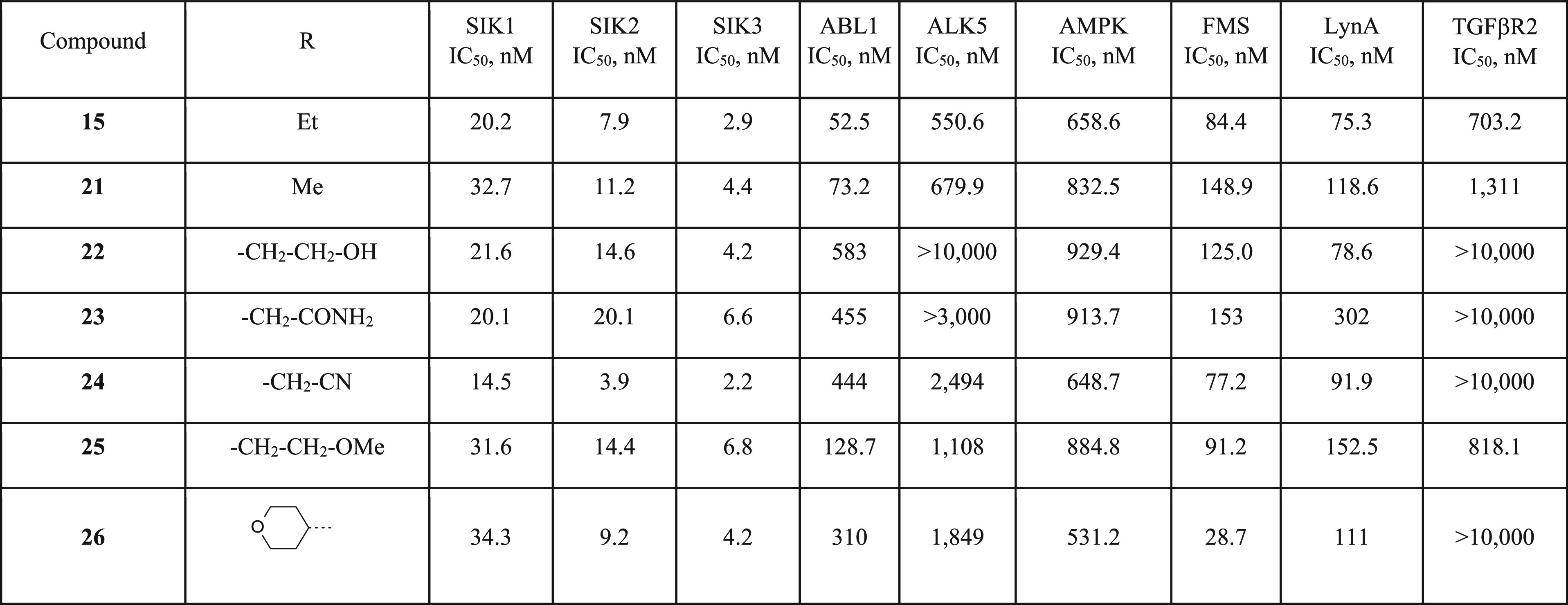
Exploration of Pyrazole Alkylation

### Co-crystal Structure of SIK3 with 22

**Figure 2:**
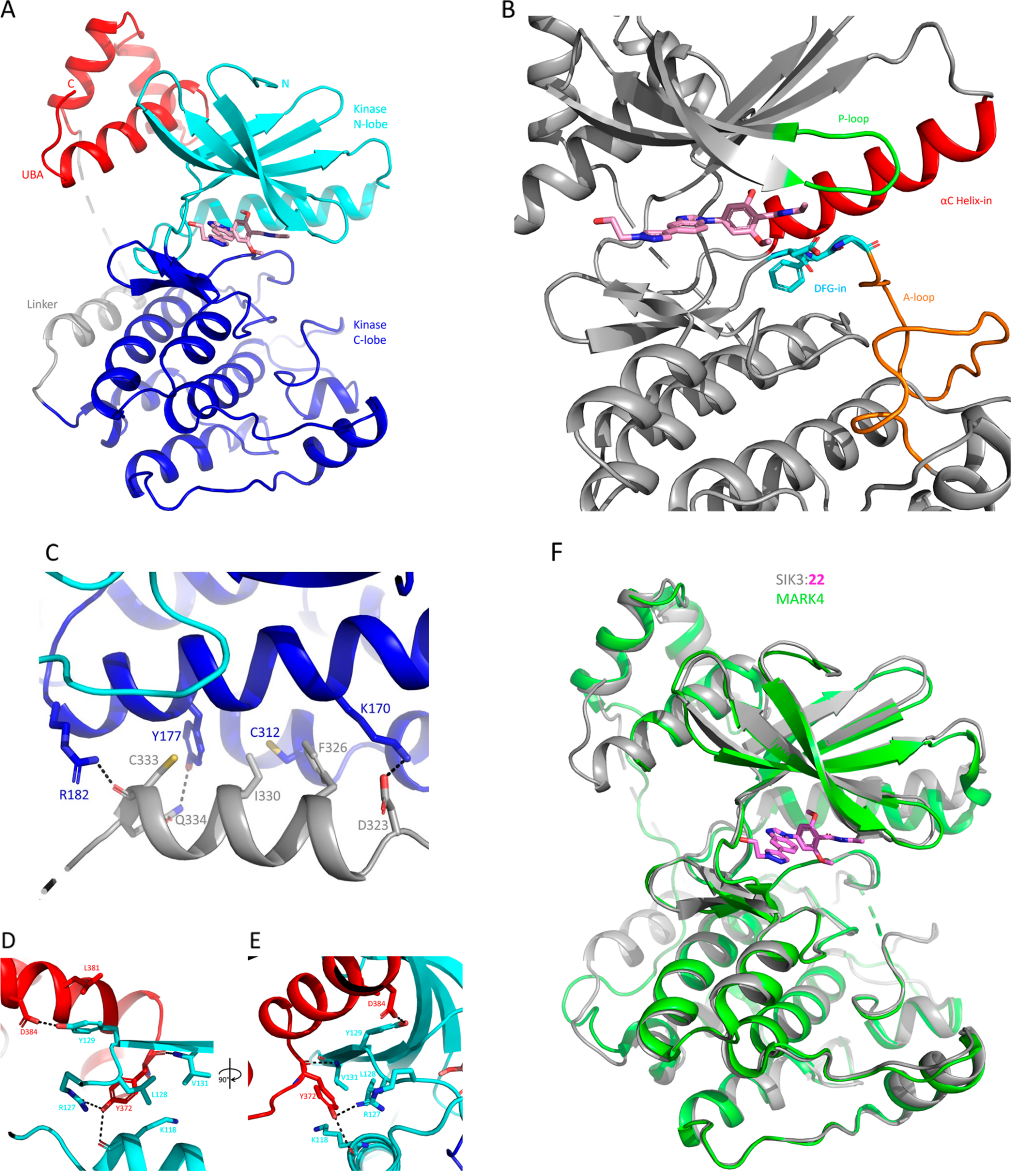
(A) Crystal structure of SIK3 (60–394 T221D) in a complex with **22**. The *N*- and *C*-lobes of the kinase domain are colored cyan and dark blue, respectively, with **22** (pink) binding in the catalytic pocket. The UBA domain, colored red, is connected to the kinase domain via a linker (gray) and packs against the kinase *N*-lobe. (B) Crystal structure of SIK3 (gray) around the binding site of **22** (pink), highlighting important regions such as P-loop (green), αC-helix-in (red), DFG-in motif (blue), and A-loop (orange). (C) Interface between kinase domain and linker region connecting to the UBA domain, colored as in (A). Key residues are shown as sticks and labeled. Hydrogen bond interactions are presented as dashed lines. (D) Interface between the *N*-lobe of the kinase domain and UBA domain. (E) Same region as (D) but rotated 90 deg. (F) Superimposition of SIK3 (gray) with **22** (pink) and MARK4 (green, PDB ID: 5ES1), RMSD: 0.82 Å.

To our knowledge, no crystal structure from the SIK family has been reported, and we disclose here the first experimentally determined crystal structure from the SIK family. The crystal structure of SIK3 (60-394 T221D) in a complex with **22** was determined to 3.1 Å. The structure contains a classical bilobed kinase catalytic domain with a flexible hinge region connecting the two lobes and forming a hydrophobic cleft serving as the binding site for ATP where compound **22** is bound (Figure 2A). The *N*-terminal lobe consists of five β-sheets and one α-helix called αC. The first two β-sheets (called β1 and β2) are linked by a loop (P-loop), which confers additional flexibility to this region (Figure 2B). The *C*terminal lobe is mainly α-helical and contains a tripeptide motif, DFG (Asp-Phe-Gly), that marks the beginning of the activation segment (A-loop). Kinases can adopt catalytically active or inactive conformations that regulate their function.^19^ In the active conformation, the aspartate of the DFG motif points into the ATP-binding site (DFG-in conformation), and in the inactive conformation, it points to the back-pocket (DFG-out). The second key feature of the active conformation for kinases is the orientation of the αC helix, which in an active state is rotated inward toward the active site (αC-helix-in). In the crystal structure, the kinase domain of SIK3 adopts an active-like conformation (DFG-in, αC-helix-in). The kinase catalytic domain is connected by a linker to an α-helical ubiquitin-associated (UBA) domain. The linker contains an α-helical segment which is locked in place via both hydrophobic and electrostatic interactions with the kinase *C*-lobe (Figure 2C). The UBA domain packs onto the *N*-terminal lobe of the catalytic domain, forming an extensive interface consisting of 536 Å^2^ in buried surface area,^20^ distal to the catalytic cleft where **22** is located (Figure 2D,E). This domain arrangement closely resembles that of other AMPK-related kinase (ARK) family members (MARK1–4) (Figure 2F).^21–24^

**Figure 3:**
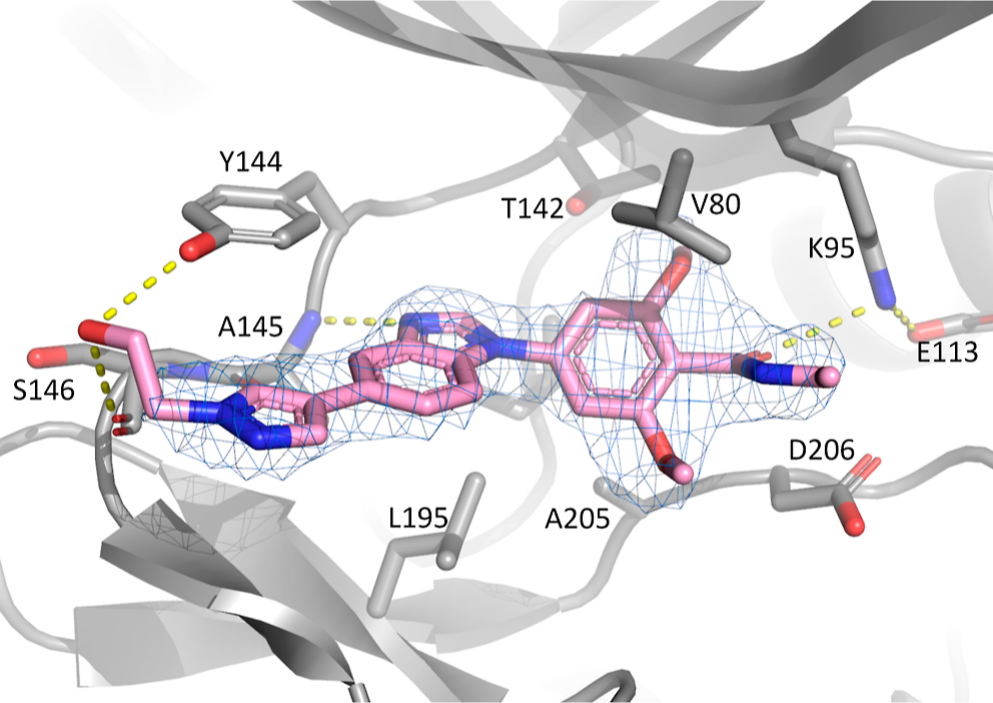
X-ray structure of **22** in complex with SIK3 (3.1 Å resolution). SIK3 is represented as a gray cartoon, with key interacting residues as gray sticks. Compound **22** is shown as pink sticks. Hydrogen bond interactions are shown as dotted yellow lines. A feature-enhanced map contoured at 1.0σ around the ligand is displayed as a blue mesh. The tip of the ligand hydroxyethyl chain is not well resolved, and the hydroxyl group is suitably positioned to form hydrogen bonds with either the backbone carbonyl of Ser146 or the side chain of Tyr144.

As mentioned above, compound **22** binds in the ATP site with the protein adopting an active-like conformation (DFG-in, αC-helix-in) and as such can be classed as a type 1 kinase inhibitor. The benzimidazole nitrogen of **22** establishes a hydrogen bond interaction with the backbone NH of Ala145 at the hinge (Figure 3). The phenyl ring is out of plane relative to the benzimidazole scaffold, and the side chains of Val80 and Ala205 provide lipophilic contacts to the substituted phenyl group. The electron density maps support the modeled orientation of the ethyl amide chain, pointing toward the solvent region. The amide group forms a hydrogen bond contact with Lys95 but not with Asp206. The proximity of the NH of the amide group and the methoxy substituent on the phenyl ring suggests that in a flexible environment an internal hydrogen bond interaction could occur, helping the orientation of the carbonyl group of the amide to interact with Lys95. The pyrazole ring is coplanar with the benzimidazole scaffold, allowing a displaced π–π interaction with Tyr144 and a weak hydrogen bond between the slightly polarized C–H group of the pyrazole and the carbonyl moiety of Ala145. The ligand hydroxyethyl group is suitably positioned to form hydrogen bonds with either the backbone carbonyl of Ser146 or the side chain of Tyr144, but the hydroxy tip is not well resolved in this structure, suggesting a weak interaction. The presence of the hydroxyl group in **22** is not related to a boost in potency compared with the ethyl group in **15** or methyl group in **21**, suggesting the weakness of the hydrogen bond interaction either with Ser146 or Tyr144. Another hypothesis to rationalize this effect could be that the addition of the hydroxyl group changes the hydration network in this solvent-exposed region, balancing the positive effect of the hydrogen bond between the ligand and target.

**Figure 4:**
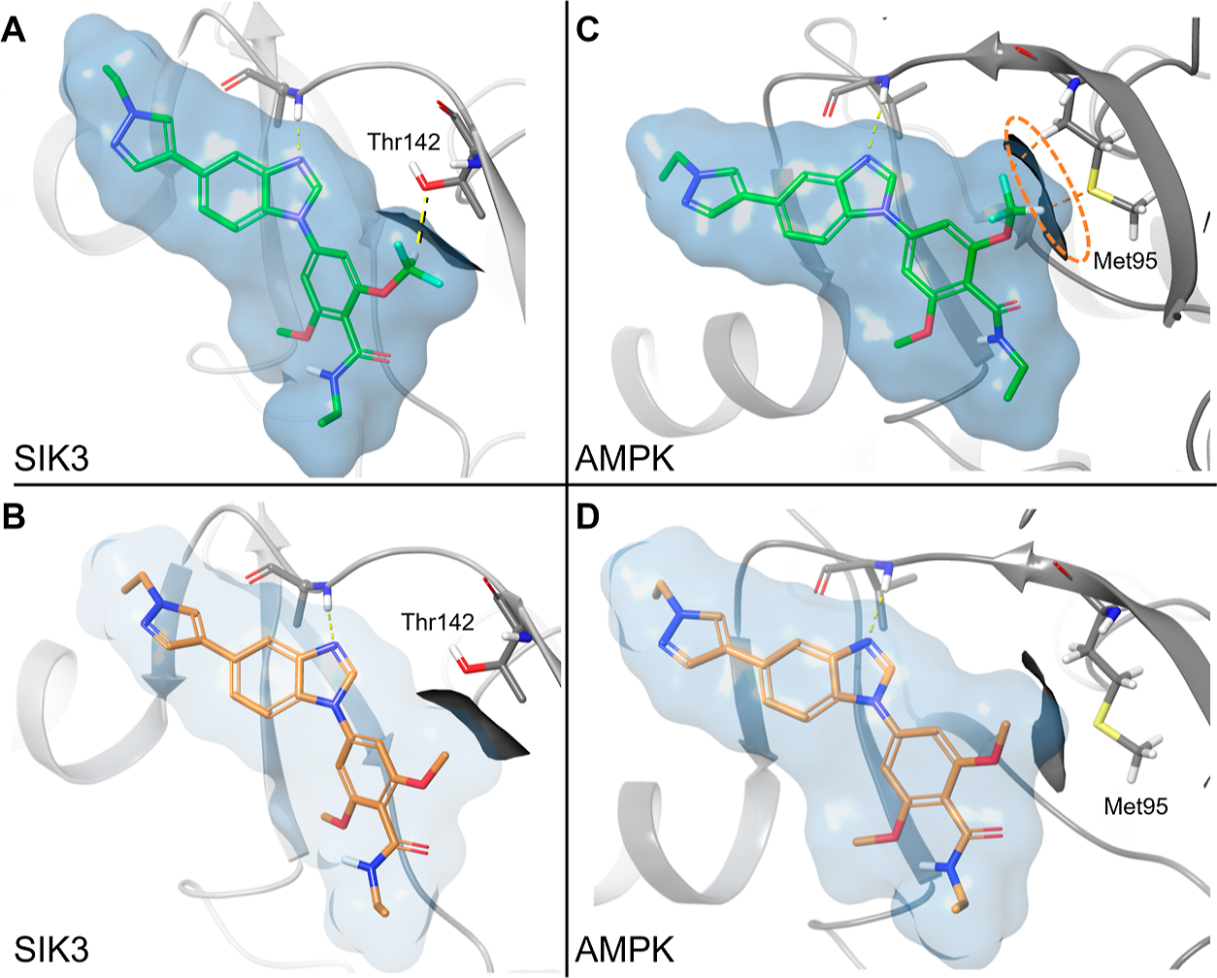
(A) Docking pose of compound **20** (shown as green sticks) in SIK3. (B) Docking pose of compound **15** (shown as orange sticks) in SIK3. (C) Docking pose of compound **20** (shown as green sticks) in AMPK (PDB ID 7JHG). (D) Docking pose of compound **15** (shown as orange sticks) in AMPK (PDB ID 7JHG). Zoom-in view of the back-pocket. The surfaces of **20** and **15** are represented in blue, while the surfaces of Thr142 and Met95 are shown in black. The steric hindrance between Met95 and the OCHF_2_ group is highlighted with an orange dashed line.

The crystal structure enabled analysis of the effects of different substitutions on selectivity and potency of the compounds by comparison with structures of other kinases. Kinases contain a single residue in the ATP-binding site, known as a gatekeeper residue, that separates the adenine binding site from an adjacent hydrophobic pocket usually called back-pocket. When one of the methoxy groups of compound **15** is replaced by a difluoromethoxy moiety in compound **20** (Table 4), a loss of activity against AMPK was observed. A likely explanation is the difference of the gatekeeper residue between the SIK family and AMPK. In the SIK family, the threonine gatekeeper (SIK3 Thr142) results in a back-pocket that can accommodate the methoxy and difluoromethoxy groups (Figure 4A,B). In contrast, the presence of a methionine gatekeeper (Met95) in AMPK reduces the volume of the back-pocket, leading to a possible clash with the larger difluoromethoxy group, whereas the methoxy group would be tolerated (Figure 4C,D). Moreover, potential interaction through a hydrogen bond between the polarized hydrogen of the difluoromethoxy moiety and the hydroxyl group of the side chain of the threonine gatekeeper in the SIK family (SIK3 Thr142, Figure 4A) could explain the increased potency observed for **20** compared to **15.**

The crystal structure also revealed possible reasons for the impact of amide alkylation on the off-target selectivity (Table 4). These alkyl groups could point toward the top part of this pocket region, occupying the bottom part of the P-loop. Ethyl **15**, trifluoroethyl **16**, and cyclopropyl **17** groups are well tolerated in SIKs because of their size; however, the bulkier *tert*-butyl **18** is not, likely due to steric hindrance in this small pocket. As hypothesized in previous publications, the P-loop could play a key role in ligand binding and selectivity,^25,26^ providing a potential explanation of the impact of these substituents on the off-target selectivity. Second alkylation of the amide in **19** is not tolerated, as it increases steric bulk and leads to loss of the possible internal hydrogen bond between the –NH of the amide and the oxygen of the methoxy substituent.

In summary, we report the first crystal structure of SIK3 kinase and UBA domains in complex with compound **22**. The kinase domain of SIK3 adopts an active-like conformation (DFG-in, αC-helix-in), and compound **22** occupies the ATP binding site and hence can be classified as a type 1 kinase inhibitor. Compound **22** is stabilized in SIK3 by the hydrogen bond interactions between one nitrogen of the benzimidazole scaffold and the backbone NH of an alanine residue at the hinge, as well as between the carbonyl of the amide group of **22** and the side chain of a lysine residue. Additionally, lipophilic contacts in the binding site made between the substituted phenyl ring and hydrophobic residues and an aromatic interaction between the pyrazole ring and tyrosine side chain in the hinge result in high potency for this chemical series.

Compound **20** having a difluoromethoxy group as the replacement of a methoxy group was docked and highlighted a possible hydrogen bond interaction between the polarized hydrogen of the difluoromethoxy moiety with the hydroxyl group of the side chain of the gatekeeper threonine residue in SIKs. SAR exploration showed that the difluoromethoxy group and alkylation of the amide could enhance kinase selectivity; we hypothesize that the difference of gatekeeper residues and of flexibility of the P-loop between kinases account for the observed gain of selectivity through the generation of steric clashes.

Overall, the first experimentally determined crystal structure of SIK3 provides a unique contribution, opening new opportunities to explore the SIK family by enabling structure-based drug design, understanding the SAR within this chemical series and other known SIK inhibitors, structural comparison with other kinases to rationalize selectivity, and investigation of protein–protein interactions.

### Optimization of Mouse Pharmacokinetic Properties

Following optimization of the potency on SIKs and off-target selectivity, pharmacokinetic properties in mice were investigated next to select a potent and selective lead molecule with low clearance and high oral bioavailability to explore the impact of SIK inhibition *in vivo* in mouse models after oral dosing.

As shown previously, compound **20** is a potent and selective SIK inhibitor with IC_50_ values of 5.8 nM on SIK1, 2.3 nM on SIK2, and 1.0 nM on SIK3. Compound **20** displayed intrinsic unbound clearances of 7.16 and <1.93 L/h/kg in mouse microsomes and hepatocytes, respectively (Table 6). This good metabolic stability *in vitro* was suitable for *in vivo* characterization in mice. Following iv administration at 1 mg/kg, the compound showed a moderate total plasma clearance of 2.33 L/h/kg but a high unbound clearance of 79.0 L/h/kg. A low oral bioavailability of 12% was determined following administration of an oral dose of 15 mg/kg. Compound **27** with a methyl group replacing the ethyl group on the pyrazole ring retains similar activity on SIKs with IC_50_ values of 6.9 nM on SIK1, 3.3 nM on SIK2, and 1.1 nM on SIK3. Compound **27** showed good metabolic stability *in vitro* with intrinsic unbound clearances of <3.05 and <1.45 L/h/kg in mouse microsomes and hepatocytes, respectively. Following iv administration of **27** at 1 mg/kg, low total and moderate unbound plasma clearances of 0.758 and 22.3 L/h/kg, respectively, were observed. An oral bioavailability of 60% was determined following administration of an oral dose of 5 mg/kg of **27**. Overall, compound **27** had similar potency as compound **20** against SIKs and improved pharmacokinetic properties with lower clearance and higher oral bioavailability than **20**. Compound **28** with a cyclopropyl carboxamide replacing the ethyl carboxamide inhibits SIKs more potently than **27** with IC_50_ values of 2.0 nM on SIK1, 0.7 nM on SIK2, and 0.6 nM on SIK3. Compound **28** displayed good metabolic stability *in vitro* with intrinsic unbound clearances of 4.76 and <1.75 L/h/kg in mouse microsomes and hepatocytes, respectively. Following iv administration of **28** at 1 mg/kg, low total and unbound plasma clearances of 0.945 and 10.2 L/h/kg, respectively, were observed. An oral bioavailability of 60% was determined following administration of an oral dose of 5 mg/kg of **28**. Overall, compound **28** displayed comparable pharmacokinetic properties to compound **27** with improved potency on SIKs.

**Table 6:**
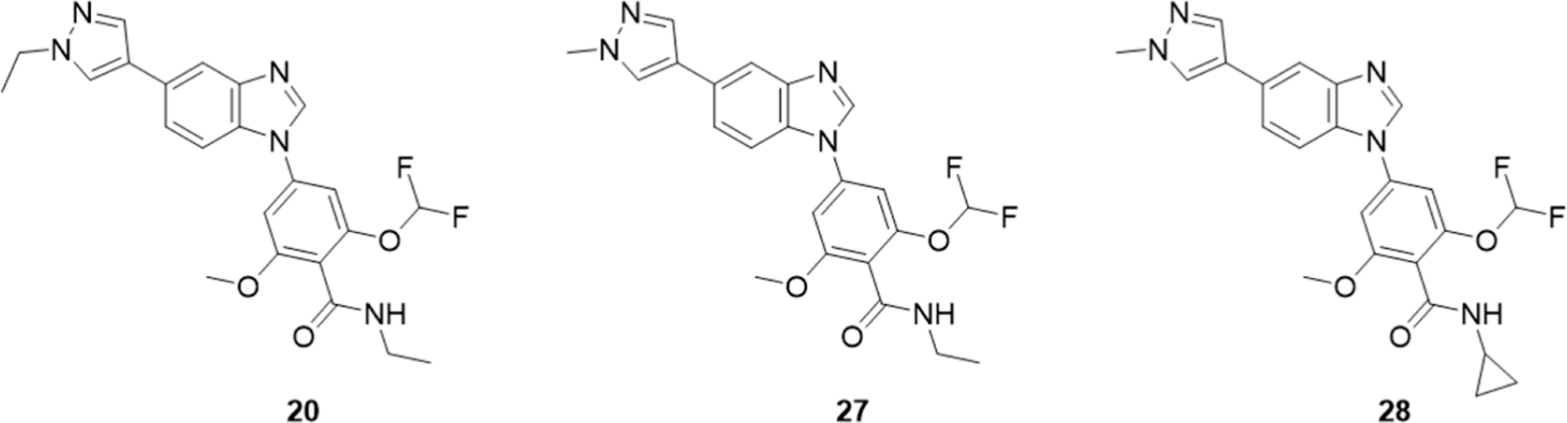
Pharmacokinetics in Mice and the Structure–Property Relationship

Compound	**20**	**27**	**28**
SIK1/SIK2/SIK3 IC_50_ (nM)	5.8/2.3/1.0	6.9/3.3/1.1	2.0/0.7/0.6
Mic CL_int,u_ (L/h/kg) mouse	7.16	<3.05	4.76
Hep CL_int,u_ (L/h/kg) mouse	<1.93	<1.46	<1.75
mouse PK (iv): 1 mg/kg			
CL (L/h/kg)	2.33	0.758	0.945
CL_u_ (L/h/kg)^a^	79.0	22.3	10.2
*V*_ss_ (L/kg)	0.815	0.722	0.723
*T*^1^/_2_ (h)	0.13	0.76	0.58
mouse PK (po):			
dose po (mg/kg)	15	5	5
F	12%	60%	60%

a Fraction unbound in mouse plasma is 0.0295, 0.034, and 0.093 for **20**, **27**, and **28**, respectively.

In summary, starting from compound **20**, shortening of the ethyl group on the pyrazole ring to a methyl group in compound **27** improved the *in vivo* total and unbound clearance and oral bioavailability. Then, replacement of ethyl carboxamide with cyclopropyl carboxamide in **28** enhanced activity on SIKs while retaining low plasma clearance and high oral bioavailability. Lead molecule **28**, also called GLPG3312, exhibited the desired pharmacokinetic properties to explore SIK inhibition *in vivo* in mouse models. *In vitro* and *in vivo* properties of compound **28** were also further characterized to assess its suitability for preclinical development.

### Rat and Dog Pharmacokinetics

Rats and dogs are the preferred species for *in vivo* toxicology investigations in preclinical development, and pharmacokinetic properties from several preclinical species are generally used to predict human pharmacokinetic properties. Thus, the pharmacokinetic properties of **28** were also evaluated in rats and dogs (Table 7). In rats, following iv administration at 1 mg/kg, **28** was characterized by a low total plasma clearance of 0.466 L/h/kg, a low unbound plasma clearance of 4.78 L/h/kg, and a moderate steady-state volume of distribution of 0.678 L/kg. The elimination half-life was 1 h. The absolute oral bioavailability was 41.4% after administration of an oral dose of 5 mg/kg. In dogs, following iv administration at 1 mg/kg, **28** was characterized by a low total plasma clearance of 0.332 L/h/kg, a low unbound plasma clearance of 1.67 L/h/kg, and a large steady-state volume of distribution of 1.76 L/kg. The apparent elimination half-life was 5.1 h. The absolute oral bioavailability was 45.5% after 30 mg/kg oral dosing. Overall, compound **28** displayed low clearance and moderate to high oral bioavailability in mice, rats, and dogs. These pharmacokinetic properties were deemed suitable for further preclinical evaluation.

**Table 7:** Pharmacokinetics of **28** in Rats and Dogs

rat PK (iv): 1 mg/kg	CL (L/h/kg)	0.466
	CL_u_ (L/h/kg)^a^	4.78
	*V*_ss_ (L/kg)	0.678
	*T*^1^/_2_ (h)	1.0
rat PK (po): 5 mg/kg	*F* %	41.4
dog PK (iv): 1 mg/kg	CL (L/h/kg)	0.332
	CL_u_ (L/h/kg)^a^	1.67
	*V*_ss_ (L/kg)	1.76
	*T*^1^/_2_ (h)	5.1
dog PK (po): 30 mg/kg	*F* %	45.5

a Fraction unbound in plasma is 0.0974 and 0.1991 in rats and dogs, respectively.

### Kinase Selectivity Profile of **28**

**Figure 5:**
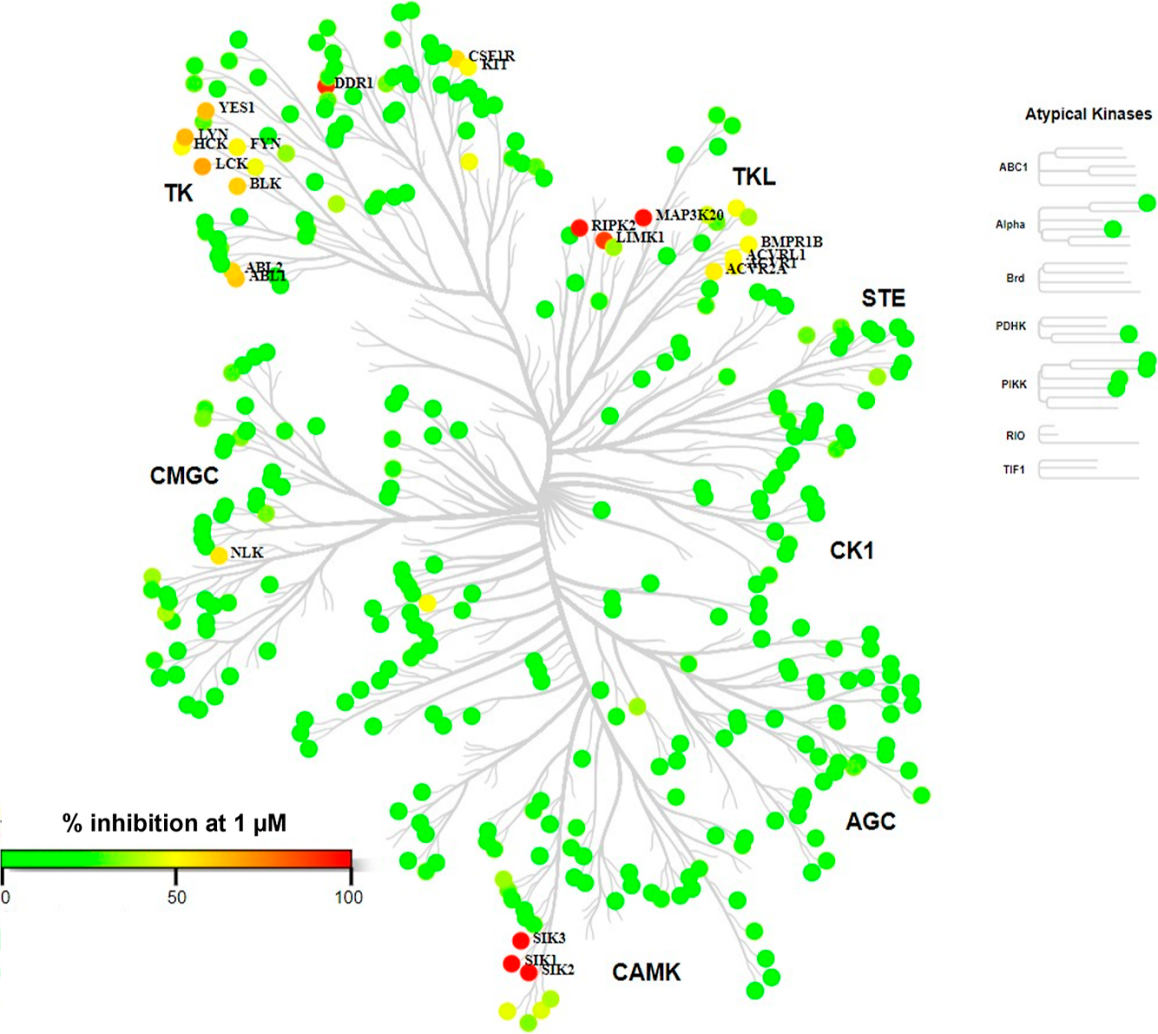
Kinome tree of **28** at 1 μM.

In addition to potent SIK inhibition and good pharmacokinetic properties, we aimed to identify a compound with good kinome selectivity to explore the therapeutic potential of SIK inhibition only. The inhibition of enzymatic activity by compound **28** at 1 μM was assessed against a panel of 380 kinases and is represented in Figure 5 (the percentage of inhibition for each kinase is available in the Supporting Information). Apart from SIKs, compound **28** showed higher than 80% inhibition at 1 μM on four other kinases: DDR1, LIMK1, MAP3K20, and RIPK2. Several kinases showed between 50 and 80% inhibition at 1 μM, and as shown in Table 8, IC_50_ was determined for all off-targets with inhibition equal to or higher than 50% at 1 μM, and the fold shift versus IC_50_ on SIK isoforms was calculated. RIPK2 was the most potent off-target identified for **28** with an IC_50_ value of 19.7 nM. IC_50_ on RIPK2 is approximately 10-fold less potent than that on SIK1 and 30-fold less potent than those on SIK2 and SIK3. The next most potent off-target kinase was DDR1 with an IC_50_ value of 57 nM. IC_50_ on DDR1 is approximately 30-fold less potent than that on SIK1 and more than 80-fold less potent than that on SIK2 and SIK3.

**Table 8:** Potency of **28** on Off-Targets from a 380 Kinase Panel Inhibited by 50% or More at 1 μM of **28** and Fold Shift versus Potency on SIK1, SIK2, and SIK3

kinase	IC_50_, nM	fold shift versus SIK1/SIK2/SIK3 IC_50_
RIPK2	19.7	9.8/28.1/32.8
DDR1	57	28.5/81.4/95
LIMK1	67.3	33.6/96.1/112.2
MAP3K20	103	51.5/147.1/171.7
LYN	272	135.9/388.3/453
ABL1	325	162.5/464.4/541.8
LCK	385	192.5/550.1/641.8
FYN	447	223.6/639/745
ABL2	493	246.6/704.6/822
YES1	598	298.8/853.9/996.2
FMS	616	308.0/880.1/1027
BLK	730	364.9/1043/1216
ACVR2A	860	429.8/1229/1433
ACVR1	992	496.2/1418/1654
NLK	1034	517.2/1478/1724
BMPR1B	1103	551.4/1575/1838
GAK	1308	654/1869/2180
ACVRL1	1333	666.7/1905/2222
KIT	1432	716.4/2047/2388
HCK	2511	1255/3587/4184

In summary, the profiling of compound **28** against a panel of 380 kinases at 1 μM showed excellent selectivity. RIPK2 was identified as the main off-target. Compound **28** is approximately 10-fold more potent on SIK1 than on RIPK2 and 30-fold more potent on SIK2 and SIK3 than on RIPK2. Compound **28** is therefore a highly selective pan-SIK inhibitor suitable to investigate SIK pharmacology *in vitro* and *in vivo*.

### Human In Vitro Pharmacodynamic Profile of **28**

**Figure 6:**
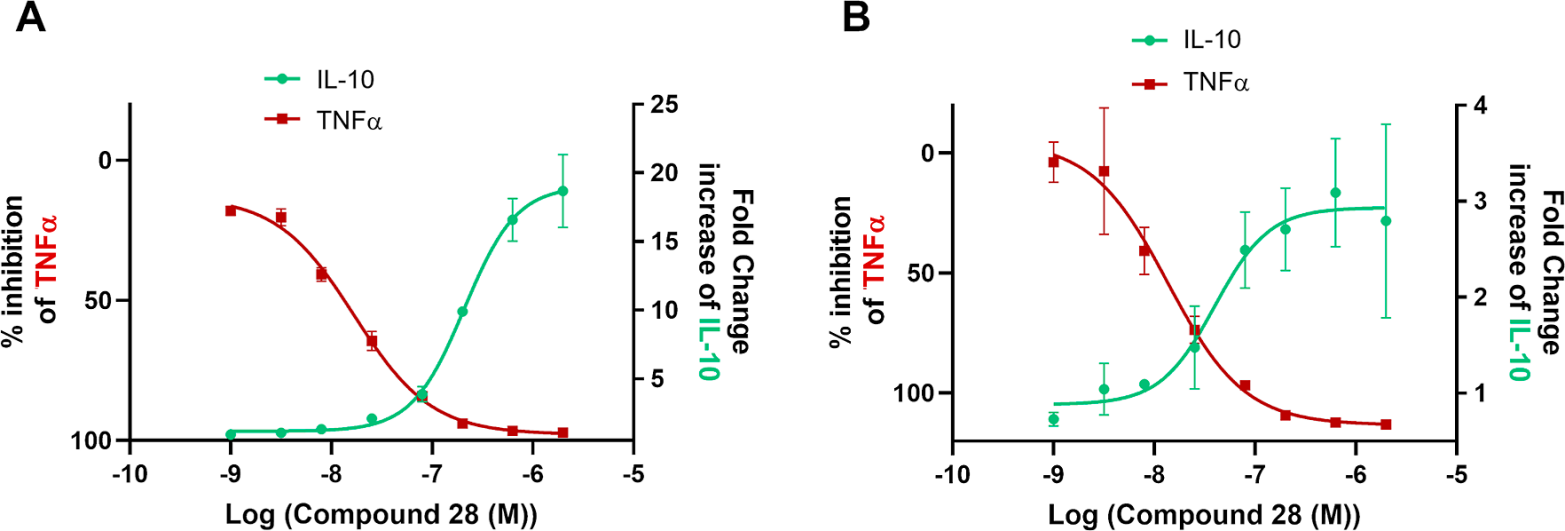
Effects of **28** on IL-10 and TNFα production in LPS-stimulated human monocytes (A) and MdM (B) from representative experiments.

Myeloid cells, including monocytes and macrophages, play key roles during the initiation, propagation, and resolution of inflammation. Upon stimulation, myeloid cells can release pro-inflammatory (e.g., TNFα) and anti-inflammatory (e.g., IL-10) cytokines. We investigated the impact of SIK inhibition on cytokine release using compound **28** in *in vitro* cell assays using primary human monocytes and monocyte-derived macrophages (MdM) stimulated with LPS. In both cell types, **28** dose-dependently inhibited TNFα release, with average IC_50_ values of 17 nM and 34 nM, respectively (Table 9). Simultaneously, compound **28** enhanced the release of IL-10 in both cell types. Data on IL-10 are expressed as fold-induction versus LPS trigger at the top concentration of 20 μM evaluated in the assay, as inaccurate curve fitting on IL-10 induction across different experiments did not allow robust EC_50_ determination. Compound **28** led to 14.8- and 2.8-fold average inductions of IL-10 at 20 μM relative to LPS-only conditions for monocytes and MdM, respectively (Figure 6 and Table 9). Generally, a higher magnitude of IL-10 induction was observed with compound **28** in monocytes compared with that in MdM. Although these observational results were not further studied, we hypothesize that differences in the expression of SIK isoforms or components of the SIK-mediated signal transduction pathway could serve as an explanation for the differences in the magnitude of IL-10 induction between both cell types. Moreover, as shown in the representative curves in Figure 6, the induction of IL-10 by compound **28** starts at higher concentrations than TNFα inhibition, which suggests that the required level of SIK inhibition might be different for the two activities.

**Table 9:** Activity of **28** in Phenotypic Cellular Assays

	TNFα pIC_50_ ± SEM/IC_50_	IL-10 fold induction ± SEM
human monocytes^a^	7.8 ± 0.13/17 nM	14.8 ± 4.7 at 20 μM
human MdM^b^	7.5 ± 0.14/34 nM	2.8 ± 0.3 at 20 μM

a Levels of TNFα and IL-10 measured 4 h post-stimulation with LPS were compared with LPS-only conditions (*n* = 6).

b Levels of TNFα (*n* = 5) and IL-10 (*n* = 4) were measured at 20 and 2 h post-LPS stimulation, respectively, and compared with LPS-only conditions at the same time point. Donors for which no accurate calculation of IC_50_ was possible on TNFα (1 donor) or that displayed a lower response of positive control than expected on IL-10 (*n* = 2) were excluded.

Overall, compound **28** inhibited the production of TNFα and increased the release of IL-10 by primary human myeloid cells stimulated by LPS. Compound **28** therefore displays both anti-inflammatory and immunoregulatory activities *in vitro*.

### Murine In Vivo Pharmacodynamic Profile of **28**

**Figure 7:**
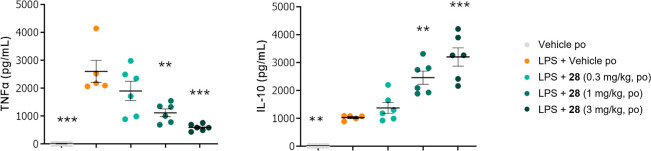
Plasma levels of TNFα and IL-10 after an in vivo LPS challenge and oral administration of **28**.

To assess *in vivo* the effect observed on TNFα and IL-10 *in vitro*, we explored the activity of **28** in an *in vivo* acute LPS challenge model in mice. In this model, stimulation by LPS elicits an immune response with increased levels of TNFα and IL-10 circulating in blood. LPS was injected intraperitoneally 15 min after oral administration of **28** at doses of 0.3, 1, and 3 mg/kg or the corresponding vehicle. Blood was collected 1.5 h post-LPS stimulation, and levels of TNFα and IL-10 in plasma were quantified. As shown in Figure 7, **28** dose-dependently reduced the release of TNFα with 27.0, 57.2, and 77.5% inhibition at 0.3, 1, and 3 mg/kg, respectively, compared with vehicle in mice stimulated with LPS. **28** also dose-dependently increased the plasma concentration of IL-10 by 1.3,- 2.4-, and 3.1-fold at 0.3, 1, and 3 mg/kg, respectively, compared with the vehicle in mice stimulated with LPS.

In summary, compound **28** inhibited the production of TNFα and increased the release of IL-10 in mice stimulated with LPS. Compound **28** therefore displays both anti-inflammatory and immunoregulatory activities *in vivo*.

## Chemistry

**Scheme 1:**
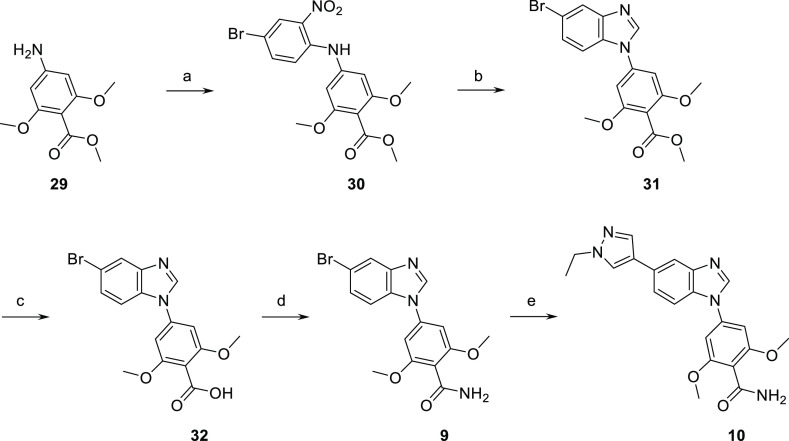
Preparation of Primary Amide Derivatives **9** and **10** Reagents and conditions: (a) 4-bromo-1-fluoro-2-nitrobenzene, LHMDS, THF, 0 °C to rt, 87%; (b) SnCl_2_, 2H_2_O, EtOH, 85 °C, then trimethyl orthoformate, 85 °C, 70%; (c) NaOH 2M, MeOH, THF, 65 °C, 94%; (d) HATU, DIPEA, ammonium chloride, DMF, rt, quantitative; and (e) 1-ethylpyrazole-4-boronic acid pinacol ester, Cs_2_CO_3_; Pd(dppf)Cl_2_.DCM, dioxane, water, 100 °C, 57%.

**Scheme 2:**
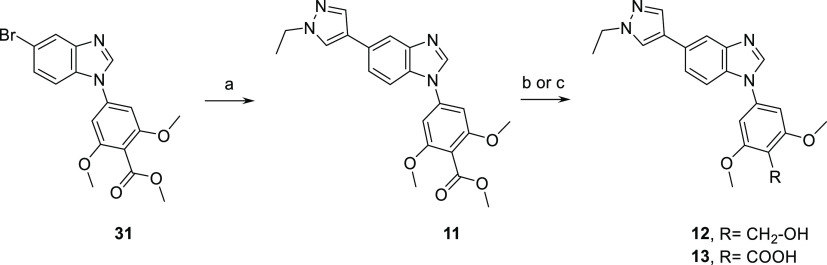
Synthesis of Compounds **11**–**13** with No Carboxamide Moiety Reagents and conditions: (a) 1-ethylpyrazole-4-boronic acid pinacol ester, Cs_2_CO_3_ Pd(PPh_3_)_4_, dioxane, water, 90 °C, 100%; (b) lithium aluminum hydride (1 M in THF), THF, rt, 87%; and (c) NaOH, MeOH, 95 °C, 65%.

**Scheme 3:**
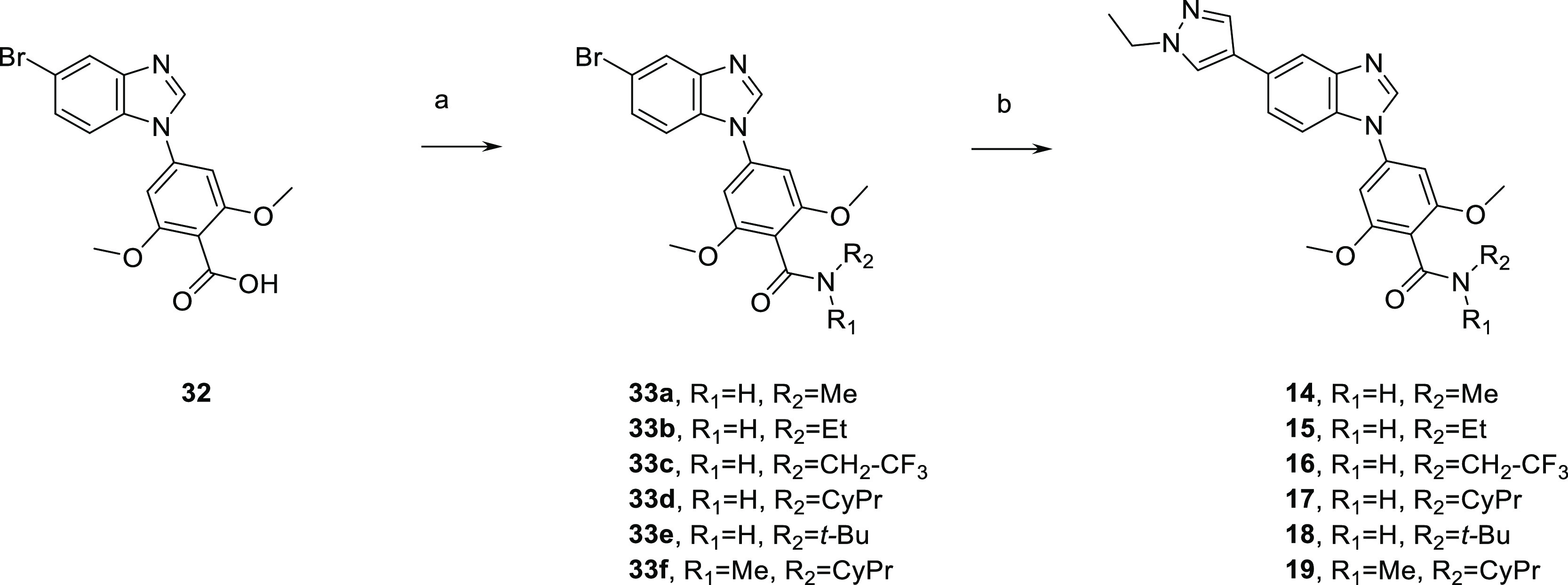
Preparation of Secondary and Tertiary Amide Derivatives **14**–**19** Reagents and conditions: (a) HATU, DIPEA, or TEA, alkylamine or alkylamine hydrochloride, DMF, rt, 50–96% and (b) 1-ethylpyrazole-4-boronic acid pinacol ester, Cs_2_CO_3_; Pd(PPh_3_)_4_, dioxane, water 90–110 °C, 29–98%.

**Scheme 4:**
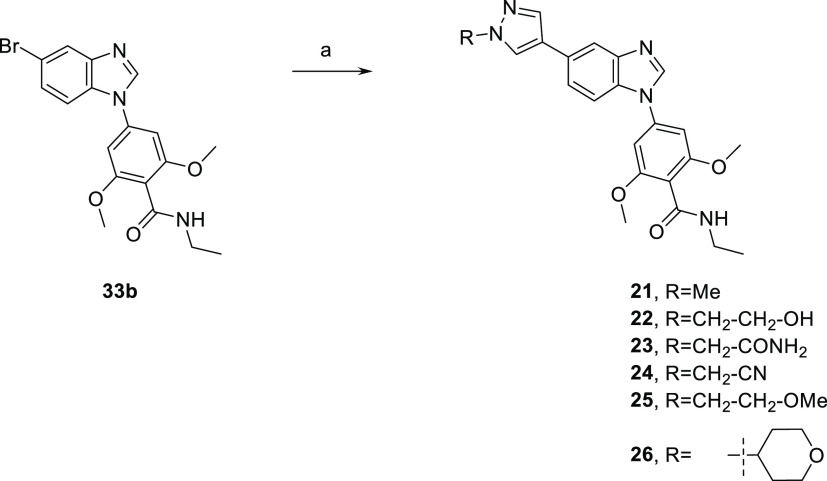
Preparation of *N*-Substituted Pyrazole Compounds **21**–**26** Reagents and conditions: (a) 1-alkylpyrazole-4-boronic acid pinacol ester, Cs_2_CO_3_, Pd(PPh_3_)_4_, dioxane, water, 90–110 °C, 14–81%.

A general method for the preparation of benzimidazole derivatives is depicted in Scheme 1.^27,28^ Nucleophilic aromatic substitution on 4-bromo-1-fluoro-2-nitrobenzene with the anion of 4-methoxycarbonyl-3,5-dimethoxyaniline **29** gave intermediate **30**. Reduction of the nitro group with stannous chloride followed by *in situ* cyclization with trimethyl orthoformate led to the construction of the benzimidazole ring in **31**. Saponification of the methyl ester of **31** and amide coupling afforded carboxamide compound **9**. 5-Ethyl pyrazole derivative **10** was then obtained by Suzuki coupling from **9**. In parallel, Suzuki coupling on methyl ester intermediate **31** gave 5-ethyl pyrazole derivative **11** (Scheme 2). Reduction of the methyl ester with lithium aluminum hydride afforded benzylic alcohol analogue **12**, and saponification of the ester gave benzoic acid analogue **13**. Secondary and tertiary amide derivatives **14**–**19** were prepared by amide coupling with carboxylic acid **32**, followed by Suzuki coupling on intermediates **33a**–**33f** (Scheme 3). Alkyl pyrazole analogues **21**–**22** and **24**–**26** were prepared from ethyl amide intermediate **33b** by Suzuki coupling with the corresponding alkyl pyrazole boronic acid pinacol ester reagents (Scheme 4). Partial hydrolysis of the alkyl nitrile group occurred upon heating under basic conditions in the Suzuki coupling to prepare **24**. Side product amide derivative **23** was isolated, and represented a valuable alkyl pyrazole analogue bearing a hydrogen bond-accepting and -donating group.

**Scheme 5:**
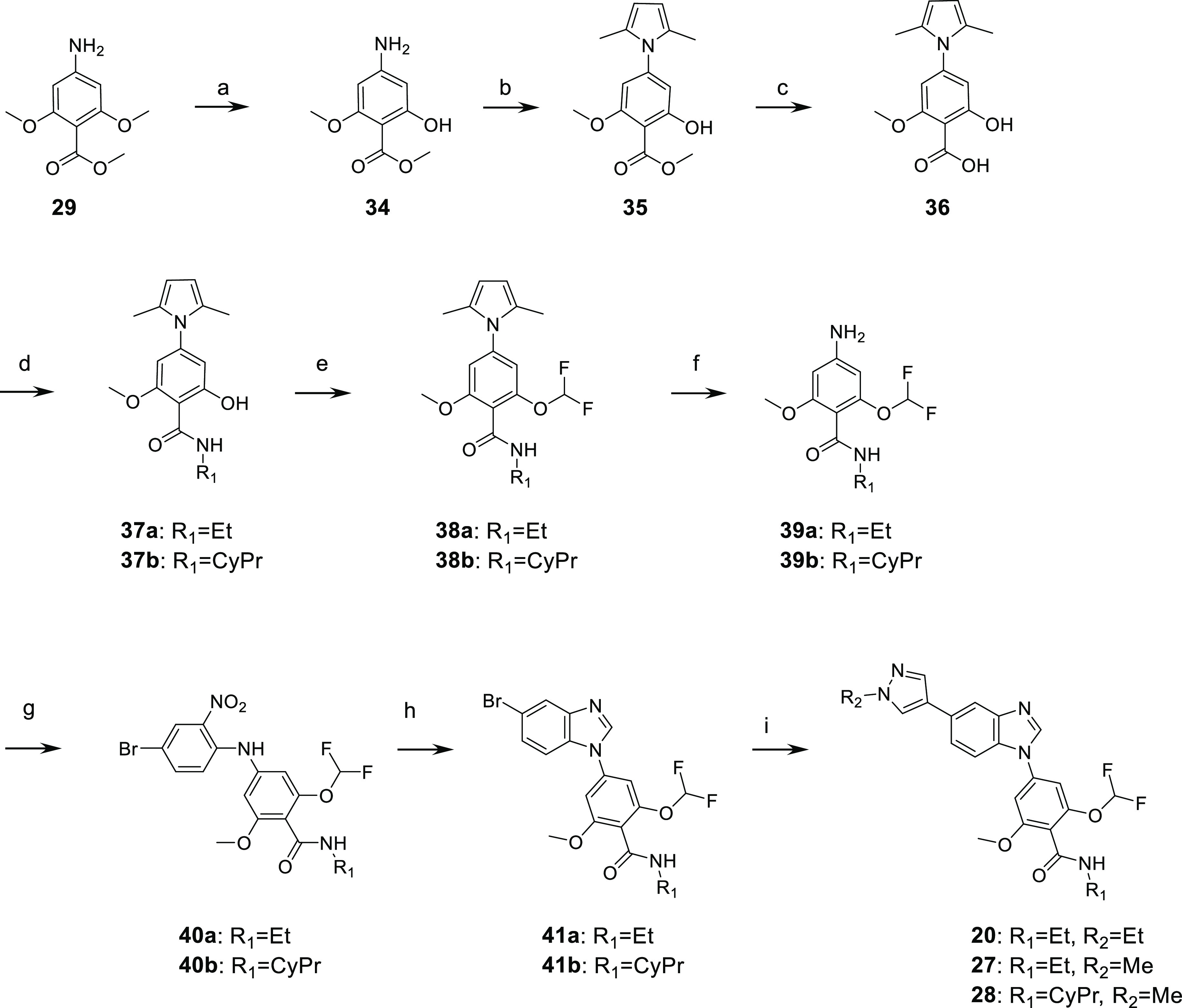
Preparation of Difluoromethoxy Derivatives **20**, **27**, and **28** Reagents and conditions: (a) BCl_3_ 1 M in DCM, 0 °C to rt, 58%; (b) 2.5-hexadione, AcOH, 110 °C, 93%; (c) NaOH 2M, MeOH reflux, 96%; (d) HATU, DIPEA, amine, DMF, rt, 78% (**37a**) and 55% (**37b**); (e) KOH, bromodifluoromethyl diethylphosphonate, ACN/H_2_O, −10 °C, 92% (**38a**) and 92% (**38b**); (f) hydroxylamine hydrochloride, EtOH/H_2_O, reflux, 75% (**39a**) and 68% (**39b**); (g) 4-bromo-1-fluoro-2-nitrobenzene, LHMDS or NaH, THF, −10 or 0 °C to rt, 27% (**40a**) and 27% (**40b**); (h) Zn dust, AcOH, rt, then PTSA, trimethyl orthoformate, MeOH, reflux, 71% (**41a**) and 73% (**41b**); and (i) 1-alkylpyrazole-4-boronic acid pinacol ester, Cs_2_CO_3_, Pd(PPh_3_)_4_ dioxane/water, 90 °C, 75% (**20**), 87% (**27**), and 84% (**28**).

As highlighted above, SAR identified the difluoromethoxy group on the phenyl ring in compounds **20**, **27** and lead molecule **28** as an important feature for potency on SIKs and selectivity against off-targets. No suitable aniline building block bearing the difluoromethoxy group was available; a dedicated synthesis of the required aniline moieties was therefore designed as shown in Scheme 5. Mono demethylation of commercially available **29** was performed with boron trichloride to generate phenol derivative **34**, and the aniline group was then protected as a 2,5-dimethylpyrrole in **35**.^29^ Saponification of the methyl ester gave **36**, which underwent amide coupling with ethyl amine and cyclopropyl amine to yield secondary amide intermediates **37a** and **37b**, respectively. Difluoromethylation of the phenol was performed using bromodifluoromethyl diethylphosphonate to give corresponding difluoromethoxy derivatives **38a** and **38b**.^30^ Dimethylpyrrole deprotection was performed with hydroxylamine in a refluxed mixture of ethanol and water, affording key aniline intermediates **39a** and **39b**. The same strategy as previously was used to construct the benzimidazole ring system. Nucleophilic aromatic substitution on 4-bromo-1-fluoro-2-nitrobenzene with the anion of **39a** and **39b** gave intermediates **40a** and **40b**, respectively, and then reduction of the nitro group in the presence of zinc dust in acetic acid and cyclization with trimethyl orthoformate in methanol led to 5-bromobenzimidazole intermediates **41a** and **41b**. Finally, Suzuki coupling with methyl or ethyl pyrazole boronic acid pinacol ester reagents afforded compounds **20**, **27** and lead molecule **28**.

## Conclusions

In summary, we have identified a series of highly potent and selective SIK inhibitors. Following an HTS campaign, a new chemotype displaying pan-SIK inhibition was identified, and SAR was explored to improve selectivity against a panel of kinases while improving potency against SIKs. The first crystal structure of SIK3 was generated, allowing a better understanding of the binding mode and selectivity of the chemical series. Optimization of pharmacokinetic properties finally led to pan-SIK inhibitor **28** (GLPG3312), which displayed low nanomolar IC_50_ for the three SIK isoforms and excellent kinase selectivity. **28** demonstrated a dual profile with both anti-inflammatory and immunoregulatory activities *in vitro* in human primary innate immune cells stimulated with LPS and *in vivo* in mice challenged with LPS. **28** was progressed into a phase 1 clinical trial evaluating a modified release exposure regimen (NCT03800472). In parallel, Galapagos investigated selective SIK2/SIK3 inhibitors and identified SIK1 inhibition as dispensable for the targeted pharmacology of SIK inhibitors. **28** was superseded by a new selective SIK2/SIK3 inhibitor candidate, GLPG3970, whose identification will be described in a future publication.

## Experimental Section

All reagents were of commercial grade and used as received, without further purification, unless otherwise stated. **8** was purchased from BioFocus, UK, as screening compound BF000743935. Testing of **28** on a 380-kinase panel and follow-up IC_50_ determination were performed at Eurofins (Eurofins Cerep, Le Bois l’Evêque, France). *Homo sapiens* SIK1 (full length, reference 02-131), ALK5 (catalytic domain aa200-503, reference 09-141), AMPKα1/β2/γ1 (full length, reference 02-147), LynA (full length, reference 08-171), and TGFβR2 (catalytic domain aa194-567, reference 09-142) were purchased from Carna Biosciences, DE. *H. sapiens* SIK2 (full length, reference PR8353A), ABL1 (full length, reference P3049), and FMS (catalytic domain aa538-910, reference PV3249) were purchased from Invitrogen, BE. AMARA peptide (AMARAASAAALARRR, A11-58) and SAMStide substrate (HMRSAMSGLHLVKRR, S07-58) were obtained from SignalChem, NL. Poly(Glu, Tyr) substrate (reference P0275), casein substrate (reference C4765), and 5′-AMP (reference A1752) were obtained from Sigma-Aldrich, BE. Commercially available anhydrous solvents were used for reactions conducted under a nitrogen or an argon atmosphere. Reagent-grade solvents were used in all other cases unless otherwise specified. Column chromatography was performed on silica gel 60 (thickness: 35–70 μm). ^1^H NMR spectra were recorded on a 400 MHz Bruker Avance spectrometer (SEI probe) or a 300 MHz DPX Bruker spectrometer (QNP probe). Chemical shifts (δ) for ^1^H NMR spectra are reported in ppm relative to tetramethylsilane (δ 0.00) or the appropriate residual solvent peak (i.e., CHCl_3_ [δ 7.27], as an internal reference). Multiplicities are given as singlet (s), doublet (d), doublet of doublet (dd), doublet of doublet of doublet (ddd), doublet of quartet (dq), doublet of triplet (dt), doublet of triplet of doublet (dtd), triplet (t), quartet (q), quintuplet (quin), multiplet (m), and broad (br). Electrospray MS spectra were obtained with a Waters Acquity UPLC instrument equipped with a Waters Acquity photodiode array detector and a single quad detector mass spectrometer. Columns used were a UPLC ethylene-bridged hybrid (BEH) C18 1.7 μm, 2.1 × 5 mm VanGuard precolumn with Acquity UPLC BEH C18 1.7 μm, 2.1 × 30 mm column or Acquity UPLC BEH C18 1.7 μm, and 2.1 × 50 mm column. All of the methods used MeCN/H_2_O gradients. MeCN and H_2_O contained either 0.1% formic acid or 0.05% NH_3_. As needed, an autopurification system from Waters was used for the LC–MS purification. LC–MS columns used were Waters XBridge Prep C18 5 μm, ODB 30 mm inner diameter (ID) × 100 mm length (L) (preparative column), and Waters XBridge C18 5 μm, 4.6 mm ID × 100 mm L (analytical column). All the methods used MeCN/H_2_O gradients. MeCN and H_2_O contained either 0.1% formic acid or 0.1% diethylamine. All final compounds reported were analyzed using these analytical methods, and purities were >95% unless otherwise indicated.

### Chemistry

#### General Procedure A for Amide Bond Forming Reaction

To the carboxylic acid derivative (1.0 equiv) in dimethylformamide (DMF) (5–8 mL per mmol of carboxylic acid) at rt, triethylamine (TEA) or N,N diisopropylethylamine (DIPEA) (2–15.0 equiv) and hexafluorophosphate azabenzotriazole tetramethyl uronium (HATU) (1.5 equiv) were added. The reaction mixture was stirred for 15 min, then alkylamine or alkylamine hydrochloride (1.2–10 equiv) was added, and stirred until full conversion (15 min–overnight). The reaction mixture was concentrated under a reduced pressure. The residue was diluted with dichloromethane (DCM) and NaHCO_3_ aqueous saturated solution. The organic layer was separated, washed with brine, dried over Na_2_SO_4_, filtered, and concentrated under reduced pressure. The crude residue was purified by flash chromatography on silica gel (eluting with a gradient of DCM/EtOAc) to afford the expected amide derivative.

#### General Procedure B for Suzuki–Miyaura Coupling

Under a nitrogen atmosphere, a solution of 5-bromo-1*H*-benzo[*d*]imidazole derivative (1.0 equiv), 1-alkylpyrazole-4-boronic acid pinacol ester (1.2–1.5 equiv), Pd(PPh_3_)_4_ (0.13–0.2 equiv), and Cs_2_CO_3_ (2.0–3.0 equiv) in dioxane/water 4:1 (8–30 mL per mmol of bromo derivative) were heated at 90–110 °C until reaction completion (15 min–overnight). At rt, DCM and brine were added, and the organic layer was dried over Na_2_SO_4_, filtered, and concentrated in vacuo. The crude residue was purified by flash chromatography on silica gel (eluting with DCM/MeOH gradient) to afford the desired compound.

##### Methyl 4-((4-Bromo-2-nitrophenyl)amino)-2,6-dimethoxybenzoate (**30**)

Under a nitrogen atmosphere, a solution of lithium hexamethyldisilazane (LHMDS) in tetrahydrofuran (THF) (0.5 M, 109.02 mL) was added dropwise over 2 h to a rapidly stirred solution of 4-methoxycarbonyl-3,5-dimethoxyaniline **29** (5.00 g, 23.70 mmol, 1.0 equiv) and 4-bromo-1-fluoro-2-nitrobenzene (5.21 g, 23.70 mmol, 1.0 equiv) in THF (125 mL), which was partially immersed in an ice-cold water bath. Upon completion of the addition, the dark purple solution was stirred for further 30 min, allowing the temperature to rise, and then the reaction was quenched with water (100 mL). THF was removed in vacuo, and then 2N HCl solution (50 mL) was added to the remaining rapidly stirred mixture. The resulting precipitate was isolated by filtration, washed with water, and dried under vacuum. The solid was then triturated with Et_2_O/hexane 60:40 and then dried under vacuum to give product **30** (8.46 g, 87% isolated yield) as a dark orange powder, which was used without further purification. LC–MS: *m*/*z* = 411.3, 413.3 [M + H].

##### Methyl 4-(5-Bromo-1*H*-benzo[*d*]imidazol-1-yl)-2,6-dimethoxybenzoate (**31**)

A mixture of nitro compound **30** (5.04 g, 12.29 mmol, 1.0 equiv), tin(II) chloride dihydrate (11.1 g, 49.2 mmol, 4.0 equiv), and ethanol (170 mL) was heated at 85 °C for 2.5 h. The mixture was cooled to rt, then trimethyl orthoformate (5.3 mL, 48.4 mmol, 3.9 equiv) was added, and the mixture was heated at 85 °C for 3.5 h. The mixture was cooled to rt, and the solvents were removed under reduced pressure. The residue was redissolved in ethyl acetate, and the solution was washed with 2 M aqueous sodium hydroxide solution, followed by saturated sodium bicarbonate solution and was then dried (MgSO_4_). The solvent was removed under reduced pressure, and the dark purple residue was triturated with diethyl ether, filtered, and washed with diethyl ether to afford **31** as a purple solid (3.36 g, 70% isolated yield) used for the next step without further purification. LC–MS: *m*/*z* = 391.3, 393.3 [M + H].

##### 4-(5-Bromo-1*H*-benzo[*d*]imidazol-1-yl)-2,6-dimethoxybenzoic acid (**32**)

Methyl ester **31** (2.97 g, 7.61 mmol, 1.0 equiv) was dissolved in methanol (20 mL) and THF (30 mL), and 2 M aqueous sodium hydroxide (20 mL) was added. The reaction mixture was stirred overnight at 65 °C. The reaction mixture was cooled to rt, and the organic solvents were removed under reduced pressure. The aqueous suspension was diluted with water and acidified with dilute hydrochloric acid until a pH between 1 and 2 was reached. After cooling in ice for 1 h, the suspension was filtered, and the resulting solid was washed with water and dried in air to afford **32** as a purple solid (2.70 g, 94% isolated yield). LC–MS: *m*/*z* = 377.2, 379.2 [M + H].

##### 4-(5-Bromo-1*H*-benzo[*d*]imidazol-1-yl)-2,6-dimethoxybenzamide (**9**)

HATU (2.8 g, 7.37 mmol, 1.2 equiv) was added to a stirred solution of carboxylic acid **32** (2.3 g, 6.1 mmol, 1.0 equiv) and diisopropylethylamine (3.3 mL, 18.3 mmol, 3.0 equiv) in DMF (25 mL) at rt. After 10 min, ammonium chloride (1.0 g, 18.3 mmol, 3.0 equiv) was added, and the reaction mixture was stirred overnight. Most of the solvent was removed under reduced pressure, and the residue was treated with water (100 mL) and stirred vigorously. The resulting solid was filtered, washed with water, and dried under vacuum to afford the desired product, **9**, as a gray solid (2.28 g, quantitative isolated yield). ^1^H NMR (400 MHz, DMSO-*d*_6_): δ ppm 8.67 (s, 1H), 8.01 (d, *J* = 1.9 Hz, 1H), 7.70 (d, *J* = 8.6 Hz, 1H), 7.61 (s, 1H), 7.50 (dd, *J* = 8.7, 1.9 Hz, 1H), 7.32 (s, 1H), 6.97 (s, 2H), 3.84 (s, 6H). LC–MS: *m*/*z* = 376.0, 378.0 [M + H].

##### 4-(5-(1-Ethyl-1*H*-pyrazol-4-yl)-1*H*-benzo[*d*]imidazol-1-yl)-2,6-dimethoxybenzamide (**10**)

In a sealed vial, under a nitrogen atmosphere, to a solution of **9** (0.093 g, 0.25 mmol, 1.0 equiv) and 1-ethyl-4-(4,4,5,5-tetramethyl-1,3,2-dioxaborolan-2-yl)pyrazole (0.068 g, 0.30 mmol, 1.2 equiv) in a mixture (4:1) of dioxane (1.2 mL) and water (0.3 mL), Cs_2_CO_3_ (161 mg, 0.49 mmol, 2.0 equiv) and 1,1′-Pd(dppf)Cl_2_.DCM (21.2 mg, 0.024 mmol, 0.1 equiv) were added. The reaction mixture was stirred at 100 °C for 1 h. The reaction mixture was cooled to rt and concentrated in vacuo. The crude residue was purified by preparative LCMS to afford compound **10** (55 mg, 57% isolated yield). ^1^H NMR (400 MHz, DMSO-*d*_6_): δ ppm 8.59 (s, 1H), 8.24 (d, *J* = 0.8 Hz, 1H), 7.99 (dd, *J* = 1.7, 0.6 Hz, 1H), 7.93 (d, *J* = 0.8 Hz, 1H), 7.70 (dd, *J* = 8.5, 0.7 Hz, 1H), 7.63–7.55 (m, 2H), 7.34–7.29 (m, 1H), 6.98 (s, 2H), 4.16 (q, *J* = 7.3 Hz, 2H), 3.85 (s, 6H), 1.43 (t, *J* = 7.3 Hz, 3H). LC–MS: *m*/*z* = 392.3 [M + H].

##### Methyl 4-(5-(1-Ethyl-1*H*-pyrazol-4-yl)-1*H*-benzo[*d*]imidazol-1-yl)-2,6-dimethoxybenzoate (**11**)

In a sealed vial, under a nitrogen atmosphere, **31** (60 mg, 0.15 mmol, 1 equiv) was dissolved in 10 mL of a degassed mixture of dioxane/water 4:1. 1-Ethylpyrazole-4-boronic acid pinacol ester (41 mg, 0.18 mmol, 1.2 equiv), Pd(PPh_3_)_4_ (27 mg, 0.023 mmol, 0.15 equiv), and Cs_2_CO_3_ (100 mg, 0.31 mmol, 2.0 equiv) were added. The reaction mixture was stirred at 90 °C for 1.5 h. The reaction was cooled to rt, and aqueous NaHCO_3_ was added and then DCM. The aqueous layers were extracted two times with DCM, and combined organic layers were dried over Na_2_SO_4_, filtered, and concentrated under reduced pressure. The crude residue was purified by flash chromatography on silica gel (eluting with DCM/MeOH 100/0 to 97/3). **11** (62 mg, 100% isolated yield) was obtained as an off-white solid. ^1^H NMR (400 MHz, chloroform-*d*): δ ppm 8.12 (s, 1H), 7.97 (d, *J* = 1.2 Hz, 1H), 7.85 (d, *J* = 0.8 Hz, 1H), 7.72 (s, 1H), 7.52 (d, *J* = 1.5 Hz, 2H), 6.72 (s, 2H), 4.26 (q, *J* = 7.3 Hz, 2H), 3.98 (s, 3H), 3.90 (s, 6H), 1.58 (t, *J* = 7.3 Hz, 3H). LC–MS: *m*/*z* = 407.5 [M + H].

##### (4-(5-(1-Ethyl-1*H*-pyrazol-4-yl)-1*H*-benzo[*d*]imidazol-1-yl)-2,6-dimethoxyphenyl)methanol (**12**)

A vial was charged with **11** (6.1 mg, 0.015 mmol, 1.0 equiv) in dry THF (2 mL) under a nitrogen atmosphere, and a solution of lithium aluminum hydride (1 M in THF, 0.15 mL, 0.15 mmol, 10 equiv) was added at rt. After 30 min, water (1 mL) was added, the mixture was poured in CHCl_3_ (50 mL), and then *n*-BuOH (5 mL) and water (50 mL) were added. The organic layer was collected, washed with brine (50 mL), and dried over MgSO_4_. After filtration, volatiles were removed from the filtrate via rotary evaporation. The residue was charged onto a column of silica gel and eluted with a gradient of DCM/i-PrOH (1:0 to 4:1) to give **12** (5.0 mg, 87% isolated yield) as a white solid. ^1^H NMR (400 MHz, DMSO-*d*_6_): δ ppm 12.70 (s, 1H), 8.58 (s, 1H), 8.24 (d, *J* = 0.8 Hz, 1H), 8.14 (s, 1H), 7.98 (d, *J* = 1.6 Hz, 1H), 7.93 (d, *J* = 0.8 Hz, 1H), 7.74–7.67 (m, 1H), 7.57 (dd, *J* = 8.4, 1.7 Hz, 1H), 6.93 (s, 2H), 4.51 (s, 2H), 4.16 (q, *J* = 7.3 Hz, 2H), 3.88 (s, 6H), 1.42 (t, *J* = 7.3 Hz, 3H). LC–MS: *m*/*z* = 379.2 [M + H].

##### 4-(5-(1-Ethyl-1*H*-pyrazol-4-yl)-1*H*-benzo[*d*]imidazol-1-yl)-2,6-dimethoxybenzoic acid (**13**)

In a sealed vial, **11** (48 mg, 0.118 mmol, 1.0 equiv) was dissolved in MeOH (8 mL). One pellet of NaOH (approximately 100 mg, 2.5 mmol, 21.0 equiv) was added, and the mixture was stirred at 95 °C for 24 h. A second pellet of NaOH (approximately 100 mg, 2.5 mmol, 21.0 equiv) was added, and the mixture was stirred at 95 °C until reaction completion. The reaction mixture was cooled to rt, and the solution was acidified with aqueous HCl 2N until a pH between 1 and 2 was reached. DCM was added, and the organic layer was washed with brine and dried over MgSO_4_. Solvents were removed in vacuo to give compound **13** (30 mg, 65% isolated yield) as a white solid. ^1^H NMR (400 MHz, DMSO-*d*_6_): δ ppm 13.00 (s, 1H), 8.83 (s, 1H), 8.28 (d, *J* = 0.8 Hz, 1H), 7.98 (dd, *J* = 15.9, 1.2 Hz, 2H), 7.78 (d, *J* = 8.5 Hz, 1H), 7.64 (dd, *J* = 8.5, 1.5 Hz, 1H), 7.05 (s, 2H), 4.16 (q, *J* = 7.3 Hz, 2H), 3.88 (s, 6H), 1.43 (t, *J* = 7.3 Hz, 3H). LC–MS: *m*/*z* = 393.3 [M + H].

##### 4-(5-Bromo-1*H*-benzo[*d*]imidazol-1-yl)-2,6-dimethoxy-*N*-methylbenzamide (**33a**)

Carboxylic acid **32** was treated with methylamine hydrochloride according to general procedure A to afford the desired product, **33a** (51 mg, 50% isolated yield), as a pale pink solid. ^1^H NMR (400 MHz, DMSO-*d*_6_): δ ppm 8.67 (s, 1H), 8.08 (d, *J* = 4.8 Hz, 1H), 8.01 (d, *J* = 1.9 Hz, 1H), 7.70 (d, *J* = 8.6 Hz, 1H), 7.50 (dd, *J* = 8.7, 1.9 Hz, 1H), 6.98 (s, 2H), 3.83 (s, 6H), 2.71 (d, *J* = 4.6 Hz, 3H). LC–MS: *m*/*z* = 390.0, 391.9 [M + H].

##### 4-(5-Bromo-1*H*-benzo[*d*]imidazol-1-yl)-*N*-ethyl-2,6-dimethoxybenzamide (**33b**)

Carboxylic acid **32** was treated with ethylamine according to general procedure A to afford the desired product, **33b** (80 mg, 75% isolated yield), as a white solid. ^1^H NMR (400 MHz, DMSO-*d*_6_): δ ppm 8.67 (s, 1H), 8.11 (t, J = 5.6 Hz, 1H), 8.00 (dd, *J* = 1.9, 0.5 Hz, 1H), 7.69 (dd, *J* = 8.6, 0.5 Hz, 1H), 7.50 (dd, *J* = 8.7, 1.9 Hz, 1H), 6.97 (s, 2H), 3.82 (s, 6H), 3.20 (qd, *J* = 7.2, 5.5 Hz, 2H), 1.08 (t, *J* = 7.2 Hz, 3H). LC–MS: *m*/*z* = 404.1, 406.1 [M + H].

##### 4-(5-Bromo-1*H*-benzo[*d*]imidazol-1-yl)-2,6-dimethoxy-*N*-(2,2,2-trifluoroethyl)benzamide (**33c**)

Carboxylic acid **32** was treated with 2,2,2-trifluoroethylamine hydrochloride according to general procedure A to afford the desired product, **33c** (45 mg, 76% isolated yield). ^1^H NMR (400 MHz, DMSO-*d*_6_): δ ppm 8.88 (t, *J* = 6.4 Hz, 1H), 8.69 (s, 1H), 8.01 (d, *J* = 1.9 Hz, 1H), 7.71 (d, *J* = 8.7 Hz, 1H), 7.50 (dd, *J* = 8.7, 1.9 Hz, 1H), 7.01 (s, 2H), 4.00 (td, *J* = 9.9, 6.4 Hz, 2H), 3.83 (s, 6H). LC–MS: *m*/*z* = 458.1, 460.1 [M + H].

##### 4-(5-Bromo-1*H*-benzo[*d*]imidazol-1-yl)-*N*-cyclopropyl-2,6-dimethoxybenzamide (**33d**)

Carboxylic acid **32** was treated with cyclopropylamine according to general procedure A to afford the desired product, **33d** (52 mg, 96% isolated yield). ^1^H NMR (400 MHz, chloroform-*d*): δ ppm 8.09 (s, 1H), 8.03 (dd, *J* = 4.9, 1.8 Hz, 1H), 7.51–7.44 (m, 1H), 7.44–7.37 (m, 1H), 6.64 (s, 2H), 5.94 (d, *J* = 3.2 Hz, 1H), 3.89 (d, *J* = 17.7 Hz, 6H), 2.97 (tq, *J* = 7.2, 3.7 Hz, 1H), 0.96–0.83 (m, 2H), 0.74–0.62 (m, 2H). LC–MS: *m*/*z* = 416.1, 417.9 [M + H].

##### 4-(5-Bromo-1*H*-benzo[*d*]imidazol-1-yl)-*N*-(*tert*-butyl)-2,6-dimethoxybenzamide (**33e**)

Carboxylic acid **32** was treated with *tert*-butylamine according to general procedure A to afford the desired product, **33e** (46 mg, 90% isolated yield). ^1^H NMR (400 MHz, chloroform-*d*): δ ppm 8.09 (s, 1H), 8.03 (d, *J* = 1.7 Hz, 1H), 7.47 (dd, *J* = 8.6, 1.6 Hz, 1H), 7.40 (d, *J* = 8.7 Hz, 1H), 6.64 (s, 2H), 5.58 (s, 1H), 3.88 (s, 6H), 1.50 (s, 9H). LC–MS: *m*/*z* = 432.3, 434.3 [M + H].

##### 4-(5-Bromo-1*H*-benzo[*d*]imidazol-1-yl)-*N*-cyclopropyl-2,6-dimethoxy-*N*-methylbenzamide (**33f**)

Carboxylic acid **32** was treated with *N*-methylcyclopropanamine according to general procedure A to afford the desired product, **33f** (50 mg, 89% isolated yield). ^1^H NMR (400 MHz, chloroform-*d*): δ ppm 8.14 (d, *J* = 9.2 Hz, 1H), 8.05 (d, *J* = 1.8 Hz, 1H), 7.48 (dd, *J* = 8.6, 1.8 Hz, 1H), 7.41 (d, *J* = 8.6 Hz, 1H), 6.67 (d, *J* = 7.0 Hz, 2H), 3.87 (d, *J* = 8.2 Hz, 6H), 3.14 (s, 3H), 2.70 (tt, *J* = 7.3, 3.9 Hz, 1H), 0.65 (m, 2H), 0.57–0.44 (m, 2H). LC–MS: *m*/*z* = 430.1–432.1 [M + H].

##### 4-(5-(1-Ethyl-1*H*-pyrazol-4-yl)-1*H*-benzo[*d*]imidazol-1-yl)-2,6-dimethoxy-*N*-methylbenzamide (**14**)

Compound **33a** was reacted with 1-ethylpyrazole-4-boronic acid pinacol ester according to general procedure B to give **14** (20 mg, 38% isolated yield). ^1^H NMR (400 MHz, DMSO-*d*_6_): δ ppm 8.59 (s, 1H), 8.24 (s, 1H), 8.08 (q, *J* = 4.6 Hz, 1H), 7.99 (d, *J* = 1.5 Hz, 1H), 7.93 (d, *J* = 0.7 Hz, 1H), 7.70 (d, *J* = 8.5 Hz, 1H), 7.59 (dd, *J* = 8.4, 1.7 Hz, 1H), 6.99 (s, 2H), 4.17 (q, *J* = 7.3 Hz, 2H), 3.84 (s, 6H), 2.72 (d, *J* = 4.6 Hz, 3H), 1.43 (t, *J* = 7.3 Hz, 3H). LC–MS: *m*/*z* = 406.5 [M + H].

##### *N*-Ethyl-4-(5-(1-ethyl-1*H*-pyrazol-4-yl)-1*H*-benzo[*d*]imidazol-1-yl)-2,6-dimethoxybenzamide (**15**)

Compound **33b** was reacted with 1-ethylpyrazole-4-boronic acid pinacol ester according to general procedure B to give **15** (600 mg, 71% isolated yield) as a white solid. ^1^H NMR (400 MHz, DMSO-*d*_6_): δ ppm 8.58 (s, 1H), 8.24 (d, *J* = 0.9 Hz, 1H), 8.11 (t, *J* = 5.5 Hz, 1H), 8.01–7.96 (m, 1H), 7.93 (d, *J* = 0.8 Hz, 1H), 7.69 (dd, *J* = 8.5, 0.7 Hz, 1H), 7.59 (dd, *J* = 8.5, 1.7 Hz, 1H), 6.98 (s, 2H), 4.16 (q, *J* = 7.3 Hz, 2H), 3.84 (s, 6H), 3.26–3.15 (m, 2H), 1.43 (t, *J* = 7.3 Hz, 3H), 1.08 (t, *J* = 7.2 Hz, 3H). LC–MS: *m*/*z* = 420.5 [M + H].

##### 4-(5-(1-Ethyl-1*H*-pyrazol-4-yl)-1*H*-benzo[*d*]imidazol-1-yl)-2,6-dimethoxy-*N*-(2,2,2-trifluoroethyl)benzamide (**16**)

Compound **33c** was reacted with 1-ethylpyrazole-4-boronic acid pinacol ester according to general procedure B to give **16** (12 mg, 29% isolated yield). ^1^H NMR (400 MHz, DMSO-*d*_6_): δ ppm 8.86 (t, *J* = 6.4 Hz, 1H), 8.60 (s, 1H), 8.24 (s, 1H), 7.99 (d, *J* = 1.6 Hz, 1H), 7.93 (s, 1H), 7.71 (d, *J* = 8.5 Hz, 1H), 7.59 (dd, *J* = 8.4, 1.6 Hz, 1H), 7.01 (s, 2H), 4.16 (q, *J* = 7.3 Hz, 2H), 4.09–3.95 (m, 2H), 3.85 (s, 6H), 1.43 (t, *J* = 7.3 Hz, 3H). LC–MS: *m*/*z* = 474.5 [M + H].

##### *N*-Cyclopropyl-4-(5-(1-ethyl-1*H*-pyrazol-4-yl)-1*H*-benzo[*d*]imidazol-1-yl)-2,6-dimethoxybenzamide (**17**)

Compound **33d** was reacted with 1-ethylpyrazole-4-boronic acid pinacol ester according to general procedure B to give **17** (35 mg, 85% isolated yield). ^1^H NMR (400 MHz, chloroform-*d*): δ 8.23 (d, *J* = 22.0 Hz, 1H), 7.96 (d, *J* = 1.4 Hz, 1H), 7.82 (d, *J* = 0.8 Hz, 1H), 7.74–7.66 (m, 1H), 7.56–7.46 (m, 2H), 6.71 (d, *J* = 23.9 Hz, 2H), 6.12–5.90 (m, 1H), 4.24 (qd, *J* = 7.4, 1.6 Hz, 2H), 3.89 (d, *J* = 17.3 Hz, 7H), 2.97 (tq, *J* = 7.2, 3.7 Hz, 1H), 1.56 (td, *J* = 7.3, 1.4 Hz, 3H), 0.93–0.84 (m, 2H), 0.70–0.62 (m, 2H). LC–MS: *m*/*z* = 432.4 [M + H].

##### *N*-(tert-Butyl)-4-(5-(1-ethyl-1*H*-pyrazol-4-yl)-1*H*-benzo[*d*]imidazol-1-yl)-2,6-dimethoxybenzamide (**18**)

Compound **33e** was reacted with 1-ethylpyrazole-4-boronic acid pinacol ester according to general procedure B to give **18** (30 mg, 79% isolated yield). ^1^H NMR (400 MHz, chloroform-*d*): δ ppm 8.12 (s, 1H), 8.02–7.97 (m, 1H), 7.87 (d, *J* = 1.0 Hz, 1H), 7.74 (s, 1H), 7.53 (d, *J* = 1.5 Hz, 2H), 6.71 (d, *J* = 1.1 Hz, 2H), 5.61 (s, 1H), 4.33–4.23 (m, 2H), 3.91 (d, *J* = 1.1 Hz, 6H), 1.60 (td, *J* = 7.3, 1.1 Hz, 3H), 1.53 (d, *J* = 1.1 Hz, 9H). LC–MS: *m*/*z* = 448.6 [M + H].

##### *N*-Cyclopropyl-4-(5-(1-ethyl-1*H*-pyrazol-4-yl)-1*H*-benzo[*d*]imidazol-1-yl)-2,6-dimethoxy-*N*-methylbenzamide (**19**)

Compound **33f** was reacted with 1-ethylpyrazole-4-boronic acid pinacol ester according to general procedure B to give **19** (39 mg, 98% isolated yield). ^1^H NMR (400 MHz, chloroform-*d*): δ ppm 8.11 (d, *J* = 8.7 Hz, 1H), 7.96 (q, *J* = 1.3 Hz, 1H), 7.83 (d, *J* = 0.9 Hz, 1H), 7.70 (d, *J* = 0.8 Hz, 1H), 7.56–7.46 (m, 2H), 6.69 (d, *J* = 6.8 Hz, 2H), 4.24 (q, *J* = 7.3 Hz, 2H), 3.87 (d, *J* = 8.2 Hz, 6H), 3.13 (s, 3H), 2.69 (tt, *J* = 7.3, 3.9 Hz, 1H), 1.56 (t, *J* = 7.3 Hz, 3H), 0.68–0.60 (m, 2H), 0.56–0.45 (m, 2H). LC–MS: *m*/*z* = 446.6 [M + H].

##### *N*-Ethyl-2,6-dimethoxy-4-(5-(1-methyl-1*H*-pyrazol-4-yl)-1*H*-benzo[*d*]imidazol-1-yl)benzamide (**21**)

Compound **33b** was reacted with 1-methyl-4-(4,4,5,5-tetramethyl-1,3,2-dioxaborolan-2-yl)pyrazole according to general procedure B to give **21** (20 mg, 41% isolated yield) as a white solid. ^1^H NMR (400 MHz, DMSO-*d*_6_): δ ppm 8.59 (s, 1H), 8.18 (d, *J* = 0.8 Hz, 1H), 8.13 (t, *J* = 5.5 Hz, 1H), 8.00–7.95 (m, 1H), 7.93 (d, *J* = 0.8 Hz, 1H), 7.73–7.66 (m, 1H), 7.58 (dd, *J* = 8.5, 1.7 Hz, 1H), 6.98 (s, 2H), 3.88 (s, 3H), 3.84 (s, 6H), 3.26–3.15 (m, 2H), 1.08 (t, *J* = 7.2 Hz, 3H). LC–MS: *m*/*z* = 406.4 [M + H].

##### *N*-Ethyl-4-(5-(1-(2-hydroxyethyl)-1*H*-pyrazol-4-yl)-1*H*-benzo[*d*]imidazol-1-yl)-2,6-dimethoxybenzamide (**22**)

Compound **33b** was reacted with 2-[4-(4,4,5,5-tetramethyl-1,3,2-dioxaborolan-2-yl)pyrazol-1-yl]ethanol pyrazole according to general procedure B to give **22** (16 mg, 36% isolated yield) as a white solid. ^1^H NMR (400 MHz, chloroform-*d*): δ ppm 8.16 (s, 1H), 7.95 (s, 1H), 7.84 (s, 1H), 7.75 (s, 1H), 7.49 (s, 2H), 6.69 (s, 2H), 5.77 (t, *J* = 5.7 Hz, 1H), 4.35–4.28 (m, 2H), 4.07 (t, *J* = 4.8 Hz, 2H), 3.91–3.86 (m, 7H), 3.54 (qd, *J* = 7.3, 5.6 Hz, 2H), 1.27 (t, *J* = 7.3 Hz, 3H). LC–MS: *m*/*z* = 436.3 [M + H].

##### 4-(5-(1-(Cyanomethyl)-1*H*-pyrazol-4-yl)-1*H*-benzo[*d*]imidazol-1-yl)-*N*-ethyl-2,6-dimethoxybenzamide (**24**)

Compound **33b** was reacted with 2-[4-(4,4,5,5-tetramethyl-1,3,2-dioxaborolan-2-yl)pyrazol-1-yl]acetonitrile according to general procedure B to give **24** (24 mg, 55% isolated yield). ^1^H NMR (400 MHz, chloroform-*d*): δ ppm 8.54 (s, 1H), 8.03 (d, *J* = 1.2 Hz, 1H), 7.90 (dd, *J* = 18.2, 0.8 Hz, 2H), 7.56 (d, *J* = 1.1 Hz, 2H), 6.76 (s, 2H), 5.77 (t, *J* = 5.7 Hz, 1H), 5.18 (s, 2H), 3.91 (s, 6H), 3.56 (qd, *J* = 7.3, 5.7 Hz, 2H), 1.29 (t, *J* = 7.3 Hz, 3H). LC–MS: *m*/*z* = 431.2 [M + H].

##### 4-(5-(1-(2-Amino-2-oxoethyl)-1*H*-pyrazol-4-yl)-1*H*-benzo[*d*]imidazol-1-yl)-*N*-ethyl-2,6-dimethoxybenzamide (**23**)

Compound **23** was obtained as a side product in the preparation of compound **24** from **33b**. Following isolation as the second eluting compound during purification by flash chromatography, the side product was further triturated in DCM with 3-mercaptopropyl ethyl sulfide silica (SPM32, from PhosphonicS) and filtered to give **23** (6 mg, 14% isolated yield). ^1^H NMR (400 MHz, chloroform-*d*): δ ppm 8.13 (s, 1H), 7.98 (d, *J* = 6.5 Hz, 2H), 7.80 (s, 1H), 7.58–7.45 (m, 2H), 6.71 (s, 2H), 6.34 (s, 1H), 5.76 (t, *J* = 5.5 Hz, 1H), 5.58 (s, 1H), 4.89 (s, 2H), 3.90 (s, 6H), 3.56 (qd, *J* = 7.3, 5.6 Hz, 2H), 1.33–1.24 (m, 3H). LC–MS: *m*/*z* = 449.2 [M + H].

##### *N*-Ethyl-2,6-dimethoxy-4-(5-(1-(2-methoxyethyl)-1*H*-pyrazol-4-yl)-1*H*-benzo[*d*]imidazol-1-yl)benzamide (**25**)

Compound **33b** was reacted with 1-(2-methoxyethyl)-4-(4,4,5,5-tetramethyl-1,3,2-dioxaborolan-2-yl)pyrazole according to general procedure B to give **25** (29 mg, 63% isolated yield). ^1^H NMR (400 MHz, chloroform-*d*): δ 8.08 (s, 1H), 7.96–7.93 (m, 1H), 7.85–7.80 (m, 1H), 7.79–7.76 (m, 1H), 7.50–7.45 (m, 2H), 6.67 (s, 2H), 5.87 (t, *J* = 5.7 Hz, 1H), 4.33 (t, *J* = 5.2 Hz, 2H), 3.86 (s, 6H), 3.79 (t, *J* = 5.2 Hz, 2H), 3.56–3.48 (m, 2H), 3.36 (s, 3H), 1.26 (t, *J* = 7.3 Hz, 3H). LC–MS: *m*/*z* = 450.6 [M + H].

##### *N*-Ethyl-2,6-dimethoxy-4-(5-(1-(tetrahydro-2*H*-pyran-4-yl)-1*H*-pyrazol-4-yl)-1*H*-benzo[*d*]imidazol-1-yl)benzamide (**26**)

Compound **33b** was reacted with 1-tetrahydropyran-4-yl-4-(4,4,5,5-tetramethyl-1,3,2-dioxaborolan-2-yl)pyrazole according to general procedure B to give **26** (39 mg, 81% isolated yield). ^1^H NMR (400 MHz, chloroform-*d*): δ 8.21 (s, 1H), 7.98 (s, 1H), 7.85 (d, *J* = 0.8 Hz, 1H), 7.75 (d, *J* = 0.8 Hz, 1H), 7.57–7.49 (m, 2H), 6.70 (s, 2H), 5.76 (t, *J* = 5.7 Hz, 1H), 4.47–4.34 (m, 1H), 4.21–4.10 (m, 2H), 3.88 (s, 6H), 3.64–3.47 (m, 4H), 2.21–2.12 (m, 4H), 1.27 (t, *J* = 7.3 Hz, 3H). LC–MS: *m*/*z* = 476.3 [M + H].

##### Methyl 4-Amino-2-hydroxy-6-methoxybenzoate (**34**)

BCl_3_ 1 M in DCM (91 mL, 91 mmol, 2.2 equiv) was added dropwise to a solution of methyl 4-amino-2,6-dimethoxy-benzoate (8.75 g, 41 mmol, 1 equiv) in dry DCM (230 mL) under a nitrogen atmosphere at 0 °C. The reaction mixture was stirred at 0 °C for 45 min and then at rt overnight. The reaction was quenched with the addition of HCl 2N and ice water, and the mixture was extracted twice with DCM. The combined organic layers were washed with water and brine, dried over anhydrous Na_2_SO_4_, and evaporated in vacuo to afford **34** (4.72 g, 58% isolated yield), which was used in the next step without further purification. ^1^H NMR (400 MHz, DMSO-*d*_6_): δ ppm 11.59 (s, 1H), 6.02 (s, 2H), 5.73 (d, *J* = 2.0 Hz, 1H), 5.66 (d, *J* = 1.9 Hz, 1H), 3.73 (s, 3H), 3.68 (s, 3H). LC–MS: *m*/*z* = 198.2 [M + H].

##### Methyl 4-(2,5-Dimethyl-1*H*-pyrrol-1-yl)-2-hydroxy-6-methoxybenzoate (**35**)

To a solution of **34** (4.72 g, 24 mmol, 1 equiv) in AcOH (100 mL), 2.5-hexadione (5.62 mL, 48 mmol, 2 equiv) was added, and the reaction mixture was stirred at 110 °C for 15 min and then at rt for 1.5 h. The mixture was evaporated under reduced pressure, purified by silica gel column chromatography, and eluted with heptane/EtOAc (50/50) to afford **35** (6.12 g, 93% isolated yield). ^1^H NMR (300 MHz, DMSO-*d*_6_): δ ppm 6.41 (d, *J* = 1.7 Hz, 1H), 6.33 (d, *J* = 1.7 Hz, 1H), 5.78 (s, 2H), 3.76 (d, *J* = 6.6 Hz, 6H), 2.01 (s, 6H). LC–MS: *m*/*z* = 276.3 [M + H].

##### 4-(2,5-Dimethyl-1*H*-pyrrol-1-yl)-2-hydroxy-6-methoxybenzoic acid (**36**)

To a solution of **35** (6.10 g, 22 mmol, 1.0 equiv) in MeOH (100 mL), a solution of NaOH 2N (33 mL, 66 mmol, 3.0 equiv) was added, and the reaction mixture was stirred at reflux for 18 h. MeOH was evaporated; then, the aqueous layer was acidified with HCl 2N (140 mL) and extracted with DCM three times. The combined organic layers were dried over Na_2_SO_4_, filtered off, and concentrated in vacuo to afford the expected product, **36** (5.57 g, 96% isolated yield). ^1^H NMR (400 MHz, DMSO-*d*_6_): δ ppm 6.35–6.28 (m, 2H), 5.90 (h, *J* = 1.5 Hz, 1H), 5.74 (s, 1H), 3.75 (s, 3H), 2.02 (s, 6H). LC–MS: *m*/*z* = 262.2 [M + H].

##### 4-(2,5-Dimethyl-1*H*-pyrrol-1-yl)-*N*-ethyl-2-hydroxy-6-methoxybenzamide (**37a**)

Carboxylic acid **36** was reacted with ethylammonium chloride according to general procedure A to afford the desired product, **37a** (4.78 g, 78% isolated yield). ^1^H NMR (400 MHz, DMSO-*d*_6_): δ ppm 8.72 (t, *J* = 5.8 Hz, 1H), 6.41 (d, *J* = 1.9 Hz, 1H), 6.35 (d, *J* = 1.9 Hz, 1H), 5.80 (s, 2H), 3.91 (s, 3H), 3.43–3.30 (m, 3H), 2.03 (s, 6H), 1.14 (t, *J* = 7.1 Hz, 3H). LC–MS: *m*/*z* = 289.4 [M + H].

##### *N*-Cyclopropyl-4-(2,5-dimethyl-1*H*-pyrrol-1-yl)-2-hydroxy-6-methoxybenzamide (**37b**)

Carboxylic acid **36** was reacted with cyclopropylamine according to general procedure A to afford the desired product, **37b** (6.36 g, 55% isolated yield). ^1^H NMR (400 MHz, DMSO-*d*_6_): δ ppm 13.10 (s, 1H), 8.43 (s, 1H), 6.39 (d, *J* = 1.9 Hz, 1H), 6.34 (d, *J* = 1.9 Hz, 1H), 5.78 (s, 2H), 3.85 (s, 3H), 2.85 (tq, *J* = 7.8, 4.0 Hz, 1H), 2.01 (s, 6H), 1.24 (s, 1H), 0.73 (td, *J* = 7.1, 4.7 Hz, 2H), 0.69–0.56 (m, 2H). LC–MS: *m*/*z* = 301.3 [M + H].

##### 2-(Difluoromethoxy)-4-(2,5-dimethyl-1*H*-pyrrol-1-yl)-*N*-ethyl-6-methoxybenzamide (**38a**)

To a solution of **37a** (4.78 g, 16.6 mmol, 1.0 equiv) in acetonitrile (100 mL) cooled to −10 °C, KOH (18.60 g, 332 mmol, 20 equiv) in water (100 mL) was added. Then, bromodifluoromethyl diethylphosphonate (5.89 mL, 33 mmol, 2.0 equiv) solubilized in acetonitrile was slowly added (caution: exothermicity controlled by the addition rate), and the reaction mixture was stirred at −10 °C for 45 min. The reaction mixture was quenched with a saturated aqueous solution of NaHCO_3_ and ice water, and was extracted twice with DCM. The combined organic layers were dried over Na_2_SO_4_ and evaporated under reduced pressure to afford the expected product, **38a** (5.2 g, 92% isolated yield). ^1^H NMR (400 MHz, DMSO-*d*_6_): δ ppm 8.35 (t, *J* = 5.6 Hz, 1H), 7.22 (t, *J* = 73.9 Hz, 1H), 6.88 (d, *J* = 1.6 Hz, 1H), 6.74–6.69 (m, 1H), 5.82 (s, 2H), 3.81 (s, 3H), 3.22 (qd, *J* = 7.2, 5.4 Hz, 2H), 2.03 (s, 6H), 1.08 (t, *J* = 7.2 Hz, 3H). LC–MS: *m*/*z* = 339.4 [M + H].

##### *N*-Cyclopropyl-2-(difluoromethoxy)-4-(2,5-dimethyl-1*H*-pyrrol-1-yl)-6-methoxybenzamide (**38b**)

To a stirred solution of **37b** (6.33 g, 21.07 mmol, 1.0 equiv) in acetonitrile (100 mL) at −10 °C, KOH (23.65 g, 421.40 mmol, 20 equiv) in water (100 mL) was added dropwise. The resulting mixture was stirred at −10 °C for 25 min, and bromodifluoromethyl diethylphosphonate (7.49 mL, 42.14 mmol, 2 equiv) in acetonitrile (15 mL) was added dropwise (caution: exothermicity controlled by the addition rate). LC–MS analysis showed full conversion once the addition was completed. The mixture was quenched with ice water and extracted twice with DCM. The organic layers were dried over Na_2_SO_4_, filtered, and concentrated under reduced pressure. The residue was purified on a 2 × 100 g HP column (Biotage) and eluted with 0–2% MeOH in DCM. The product fractions were combined and evaporated until dry to afford the title compound, **38b**, as a light brown solid (6.78 g, 92% isolated yield). ^1^H NMR (400 MHz, DMSO-*d*_6_): δ ppm 8.41 (d, *J* = 4.6 Hz, 1H), 7.21 (t, *J* = 73.8 Hz, 1H), 6.87 (d, *J* = 1.6 Hz, 1H), 6.73–6.68 (m, 1H), 5.82 (s, 2H), 3.80 (s, 3H), 2.79 (tt, *J* = 7.7, 3.8 Hz, 1H), 2.02 (s, 6H), 0.67 (td, *J* = 7.0, 4.7 Hz, 2H), 0.50–0.42 (m, 2H). LC–MS: *m*/*z* = 351.5 [M + H].

##### 4-Amino-2-(difluoromethoxy)-*N*-ethyl-6-methoxybenzamide (**39a**)

To a stirred solution of **38a** (5.35 g, 15.81 mmol, 1.0 equiv) in EtOH (60 mL) at rt, hydroxylamine hydrochloride (10.9 g, 151.8 mmol, 10.0 equiv) in water (30 mL) and triethylamine (4.37 mL, 31.62 mmol, 2.0 equiv) were added. The reaction mixture was refluxed overnight. EtOH was evaporated. The aqueous phase was brought to pH 10 with a Na_2_CO_3_ saturated aqueous solution and extracted with DCM. The organic layers were dried over MgSO_4_, filtered, and concentrated under reduced pressure. The residue was purified by flash chromatography on silica gel and eluted with 0–5% MeOH in DCM. The product fractions were combined and evaporated until dry to afford the title compound as a beige solid, which was further purified by flash chromatography on a KP-NH column (Biotage) and eluted with 0–2% MeOH in DCM. The product fractions were combined and evaporated until dry to afford the title intermediate, **39a**, as a white solid (3.08 g, 75% isolated yield). ^1^H NMR (400 MHz, DMSO-*d*_6_): δ ppm 7.86 (t, *J* = 5.7 Hz, 1H), 6.88 (t, *J* = 75.1 Hz, 1H), 6.09 (d, *J* = 1.8 Hz, 1H), 5.95 (d, *J* = 1.6 Hz, 1H), 5.55 (s, 2H), 3.65 (s, 3H), 3.20–3.08 (m, 2H), 1.03 (t, *J* = 7.2 Hz, 3H). LC–MS: *m*/*z* = 261.5 [M + H].

##### 4-Amino-*N*-cyclopropyl-2-(difluoromethoxy)-6-methoxybenzamide (**39b**)

To a stirred solution of **38b** (6.78 g, 19.35 mmol, 1.0 equiv) in EtOH (100 mL) at rt, hydroxylamine hydrochloride (13.45 g, 193.51 mmol, 10.0 equiv) in water (50 mL) was added. The reaction mixture was refluxed overnight. Hydroxylamine hydrochloride (6.72 g, 96.7 mmol, 5.0 equiv) and triethylamine (5.35 mL, 38.7 mmol, 2.0 equiv) were added. The reaction mixture was refluxed for 3.5 h. EtOH was evaporated. The aqueous phase was brought to pH 9 with NaOH 2N and was extracted twice with EtOAc. The organic layers were dried over Na_2_SO_4_, filtered, and concentrated under reduced pressure. The residue was purified by chromatography on silica gel and eluted with 0–5% MeOH in DCM. The product fractions were combined and evaporated until dry. The solid was triturated with Et_2_O and filtered to afford the title intermediate, **39b** (3.61 g, 68% isolated yield), as a white solid. ^1^H NMR (400 MHz, DMSO-*d*_6_): δ ppm 7.94 (d, *J* = 4.5 Hz, 1H), 6.86 (t, *J* = 75.0 Hz, 1H), 6.08 (d, *J* = 1.8 Hz, 1H), 5.94–5.92 (m, 1H), 5.56 (s, 2H), 3.65 (s, 3H), 2.70 (tt, *J* = 7.4, 3.8 Hz, 1H), 0.61 (td, *J* = 7.0, 4.6 Hz, 2H), 0.46–0.25 (m, 2H). LC–MS: *m*/*z* = 273.4 [M + H].

##### 4-((4-Bromo-2-nitrophenyl)amino)-2-(difluoromethoxy)-*N*-ethyl-6-methoxybenzamide (**40a**)

To a solution of **39a** (1.645 g, 6.32 mmol, 1 equiv) and 1-bromo-4-fluoro-3-nitrobenzene (856 μL, 7 mmol, 1.1 equiv) in dry THF (30 mL) under an argon atmosphere at −15 °C, dropwise LHMDS 1 M solution in THF (13 mL, 13 mmol, 2 equiv) was added. The reaction mixture was stirred at −10 °C for 40 min. LHMDS 1 M solution in THF (3 mL, 3 mmol) was added dropwise, and the reaction mixture was stirred at −10 °C for 1.5 h. Cold water was carefully added (caution: exothermic), followed by HCl 2N, and the mixture was stirred for 18 h at rt. The reaction mixture was diluted with DCM and water. The organic layer was separated, dried over Na_2_SO_4_, filtered, and concentrated under reduced pressure. The crude residue was purified by flash chromatography on silica gel (eluting with heptane/EtOAc: 100/0 to 90/10) to afford the title compound, **40a**, as an orange solid (773 mg, 27% isolated yield). ^1^H NMR (400 MHz, chloroform-*d*): δ ppm 8.41 (d, *J* = 7.7 Hz, 1H), 6.90 (d, *J* = 13.2 Hz, 1H), 6.83–6.32 (m, 5H), 5.82 (s, 1H), 3.87 (s, 3H), 3.52 (qd, *J* = 7.2, 5.7 Hz, 3H), 1.27 (t, *J* = 7.3 Hz, 2H). LC–MS: *m*/*z* = 460.0, 461.9 [M + H].

##### 4-((4-Bromo-2-nitrophenyl)amino)-*N*-cyclopropyl-2-(difluoromethoxy)-6-methoxybenzamide (**40b**)

To a solution of **39b** (729 mg, 2.679 mmol, 1.1 equiv) and 4-bromo-1-fluoro-2-nitrobenzene (300 μL, 2.345 mmol, 1.0 equiv) in anhydrous THF (5 mL) cooled to 0 °C under an argon atmosphere, sodium hydride (60% in oil, 292 mg, 7.306 mmol, 3.0 equiv) was added. The reaction mixture was stirred at 0 °C for 10 min and then stirred at rt for 18 h. 4-Bromo-1-fluoro-2-nitrobenzene (100 μL, 0.781 mmol, 0.36 equiv) was added, and the mixture was stirred for 1.5 h. The reaction mixture was quenched with aqueous saturated NH_4_Cl solution and extracted twice with DCM. The combined organic layers were washed with brine, dried over Na_2_SO_4_, and evaporated under reduced pressure. The crude residue was purified by flash chromatography on silica gel (heptane/EtOAc: 100/0 to 60/40) to afford the title compound, **40b**, as an orange solid (313 mg, 27% isolated yield). LC–MS: *m*/*z* = 472.1, 474.0 [M + H].

##### 4-(5-Bromo-1*H*-benzo[*d*]imidazol-1-yl)-2-(difluoromethoxy)-*N*-ethyl-6-methoxybenzamide (**41a**)

A portion of Zn dust (780 mg, 11.928 mmol, 7.1 equiv) was added to a solution of **40a** (773 mg, 1.680 mmol, 1 equiv) in glacial acetic acid (12 mL). The mixture was heated slowly until it started to boil and was then stirred at rt until full conversion of the starting material was observed by LC–MS. The reaction mixture was filtered and concentrated under reduced pressure. The crude residue was dissolved in MeOH (20 mL). *p*-Toluenesulfonic acid (PTSA) (74 mg, 0.390 mmol, 0.2 equiv) and trimethyl orthoformate (641 μL, 5.843 mmol, 3.0 equiv) were added. The reaction mixture was refluxed for 40 min and then cooled to rt and stirred for 18 h. The reaction mixture was concentrated in vacuo. The crude residue was purified by flash chromatography on silica gel (eluting with heptane/EtOAc: 100/0 to 0/100) to afford the title compound, **41a** (612 mg, 71% isolated yield). ^1^H NMR (400 MHz, chloroform-*d*): δ ppm 8.10 (s, 1H), 8.04 (d, *J* = 1.8 Hz, 1H), 7.50 (dd, *J* = 8.7, 1.8 Hz, 1H), 7.39 (d, *J* = 8.6 Hz, 1H), 7.01 (dt, *J* = 1.9, 1.1 Hz, 1H), 6.91 (d, *J* = 1.8 Hz, 1H), 6.65 (t, *J* = 73.5 Hz, 1H), 5.89 (d, *J* = 6.1 Hz, 1H), 3.93 (s, 3H), 3.55 (qd, *J* = 7.3, 5.7 Hz, 2H), 1.29 (t, *J* = 7.3 Hz, 3H). LC–MS: *m*/*z* = 441.3 [M + H].

##### 4-(5-Bromo-1*H*-benzo[*d*]imidazol-1-yl)-*N*-cyclopropyl-2-(difluoromethoxy)-6-methoxybenzamide (**41b**)

A portion of Zn dust (330 mg, 5.047 mmol, 7.6 equiv) was added to a solution of **40b** (313 mg, 0.663 mmol, 1.0 equiv) in glacial acetic acid (1 mL). The mixture was heated slowly until it started to boil and was then stirred at rt until full conversion of the starting material was observed by LC–MS. The reaction mixture was filtered and concentrated under reduced pressure. The crude residue was dissolved in MeOH (8 mL). PTSA (25 mg, 0.133 mmol, 0.2 equiv) and trimethyl orthoformate (218 μL, 1.988 mmol, 3.0 equiv) were added. The reaction mixture was refluxed for 30 min, then cooled to rt, and stirred for 18 h. The reaction mixture was concentrated in vacuo. The crude residue was purified by flash chromatography on silica gel (eluting with DCM/MeOH 100/0 to 90/10 and then EtOAc) to afford the title compound, **41b** (219 mg, 73% isolated yield). ^1^H NMR (400 MHz, chloroform-*d*): δ ppm 8.06 (s, 1H), 8.05–8.00 (m, 1H), 7.51–7.44 (m, 1H), 7.36 (d, *J* = 8.8 Hz, 1H), 6.98 (dt, *J* = 1.9, 1.1 Hz, 1H), 6.89 (d, *J* = 1.8 Hz, 1H), 6.63 (t, *J* = 73.5 Hz, 1H), 5.99 (s, 1H), 3.93 (d, *J* = 15.8 Hz, 3H), 2.94 (tq, *J* = 7.1, 3.6 Hz, 1H), 0.97–0.84 (m, 2H), 0.70–0.62 (m, 2H). LC–MS: *m*/*z* = 453.3 [M + H].

##### 2-(Difluoromethoxy)-*N*-ethyl-4-(5-(1-ethyl-1*H*-pyrazol-4-yl)-1H-benzo[*d*]imidazol-1-yl)-6-methoxybenzamide (**20**)

Compound **41a** was reacted with 1-ethylpyrazole-4-boronic acid pinacol ester according to general procedure B to give **20** as an off-white solid (312 mg, 75% isolated yield). ^1^H NMR (400 MHz, chloroform-*d*): δ ppm 8.09 (s, 1H), 7.97 (s, 1H), 7.87–7.81 (m, 1H), 7.72 (s, 1H), 7.56–7.46 (m, 2H), 7.11–7.02 (m, 1H), 6.95 (d, *J* = 1.8 Hz, 1H), 5.94 (t, *J* = 5.5 Hz, 1H), 4.25 (q, *J* = 7.3 Hz, 2H), 3.93 (s, 3H), 3.56 (qd, *J* = 7.2, 5.7 Hz, 2H), 1.57 (t, *J* = 7.3 Hz, 3H), 1.29 (t, *J* = 7.3 Hz, 3H). LC–MS: *m*/*z* = 456.5 [M + H].

##### 2-(Difluoromethoxy)-*N*-ethyl-6-methoxy-4-(5-(1-methyl-1*H*-pyrazol-4-yl)-1*H*-benzo[*d*]imidazol-1-yl)benzamide (**27**)

Compound **41a** was reacted with 1-methylpyrazole-4-boronic acid pinacol ester according to general procedure B to give the title compound, **27** (54 mg, 87% isolated yield). ^1^H NMR (400 MHz, chloroform-*d*): δ 8.08 (s, 1H), 7.94 (d, *J* = 1.2 Hz, 1H), 7.81 (d, *J* = 0.8 Hz, 1H), 7.66 (s, 1H), 7.49 (t, *J* = 1.2 Hz, 2H), 7.03 (q, *J* = 1.3 Hz, 1H), 6.94 (d, *J* = 1.7 Hz, 1H), 6.65 (t, *J* = 73.5 Hz, 1H), 5.91 (t, *J* = 5.7 Hz, 1H), 3.97 (s, 3H), 3.92 (s, 3H), 3.54 (qd, *J* = 7.3, 5.7 Hz, 2H), 1.28 (t, J = 7.2 Hz, 3H). LC–MS: *m*/*z* = 442.5 [M + H].

##### *N*-Cyclopropyl-2-(difluoromethoxy)-6-methoxy-4-(5-(1-methyl-1*H*-pyrazol-4-yl)-1*H*-benzo[*d*]imidazol-1-yl)benzamide (**28**)

Compound **41b** was reacted with 1-methylpyrazole-4-boronic acid pinacol ester according to general procedure B to give the title compound, **28** (43 mg, 84% isolated yield). ^1^H NMR (400 MHz, CDCl_3_): δ 8.20–8.01 (m, 1H), 8.00–7.92 (m, 1H), 7.83–7.80 (m, 1H), 7.68–7.65 (m, 1H), 7.54–7.44 (m, 2H), 7.07–7.01 (m, 1H), 6.99–6.91 (m, 1H), 6.64 (t, *J* = 73.6 Hz, 1H), 6.13–5.88 (m, 1H), 4.04–3.82 (m, 6H), 2.95 (dq, *J* = 7.1, 3.5 Hz, 1H), 0.97–0.87 (m, 2H), 0.73–0.62 (m, 2H). LC–MS: *m*/*z* = 454.5 [M + H].

### *H. sapiens* SIK3 Preparation

The coding sequence from amino acids 59–1321 of the *H. sapiens* SIK3 protein (ref seq: NM_025164.6 and UniProtKB/Swiss-Prot Q9Y2K2-5) was cloned into pFastBac1 with *N*-terminal (GST) and *C*-terminal (6His) affinity tags with *N*-terminal (tobacco etch virus, TEV) and *C*-terminal (thrombin, Thr) protease sites, giving a pFastBac-GST-TEV-HsSIK3(59–1321)-Thr-6His construct. The expression cassette of pFastBac-GST-TEV-HsSIK3(59–1321)-Thr-6His was recombined with the parent bacmid in DH10Bac *E. coli* competent cells (Invitrogen, FR) and the parent bacmid in EmbacY_DH10Bac *E. coli* competent cells (Geneva Biotech, CH) to form expression bacmids. Sf9 insect cells (Life Technologies) were transfected by either DH10Bac or EmbacY_DH10Bac DNA using Cellfectin II reagent (Life Technologies) to get viral stocks. Sf9 insect cells were then infected with viral stocks (P2) and were harvested after 4–6 days. Pellets were stored at −20 °C until use.

Cells were resuspended (5 vol/g of cell paste) on ice with equilibration buffer (50 mM Tris pH 8.0, 250 mM NaCl; 2 mM DTT, 1 mM EDTA, 10% glycerol, and 0.05% Brij-35) and EDTA-free protease inhibitor cocktail (Roche, FR). After homogenization by sonication, Benzonase nuclease (Merck Millipore, FR) was added to remove DNA viscosity. The sample was clarified by ultracentrifugation and filtration on 22 μm (PES) low binding filters (Corning, FR). The clarified sample was applied on a GST-Trap 4B column (Cytiva, FR), pre-equilibrated with 50 mM Tris pH 8.0, 250 mM NaCl, 2 mM DTT, 1 mM EDTA, 10% glycerol, and 0.05% Brij-35, with recirculation of the sample though the column for 24 h to improve the binding efficiency. The column was washed with 10 column volumes of equilibration buffer. Elution was done stepwise, and the SIK3 protein was successfully eluted after two column volumes of 10 mM of reduced glutathione.

The protein was further purified by using immobilized metal affinity chromatography. The sample was applied to pre-equilibrated (equilibration buffer) HisTrap HP (Cytiva, FR), and the SIK3 protein was recovered in a 300 mM imidazole elution step. Elution fractions were pooled, centrifuged to remove potential insoluble aggregates, and subjected to size exclusion chromatography (HiLoad 16/600 Superdex 75 pg [Cytiva], FR) in 50 mM Tris pH 8.0, 250 mM NaCl, 1 mM DTT, 1 mM EDTA, 10% glycerol, and 0.05% Brij-35 running buffer. A unique peak at a roughly 48 mL retention volume was detected. Elution fractions were stored at −80 °C after being flash-frozen in liquid nitrogen.

The final yield was 0.15 mg/L cell culture with a purity level of 85%.

### ADP-Glo Kinase Assay with SIKs

1.11–2.23 nM SIK1, 0.11–0.48 nM SIK2, or 0.45 nM SIK3 was incubated with 45 μM AMARA peptide and 5 μM ATP in 25 mM Tris pH 7.5, 0.5 mM EGTA, 0.01% Triton X-100, 5 mM MgCl_2_, and 2.5 mM DTT at rt for 120 min in the presence or absence of the compound. To determine IC_50_ values, compounds were tested in a 10-point dose response with a 1/5 serial dilution starting from a top concentration of 20 μM. The kinase reaction was stopped after the addition of an equal volume of ADP-Glo reagent (ADP-Glo Kinase Assay, Promega, NL) and was incubated at rt for 40 min to remove all the remaining ATP. Afterward, a double volume of kinase detection reagent was added and incubated for a minimum of 30 min at rt before the luminescence signal was measured with an EnVision PerkinElmer plate reader.

### General Procedure for ^33^P Radioactive Kinase Assay

The basis for the radioactive kinase assay is the measurement of the incorporated ^33^P into the substrate when phosphorylated by the kinase of interest using [^33^P]-γ-ATP. Briefly, the kinase of interest was incubated with the substrate, ATP, and [^33^P]-γ-ATP (PerkinElmer NV, BE) in the reaction medium at 33 °C for 45–60 min in the presence or absence of compound. To determine IC_50_ values, compounds were tested in a 10-point dose response with a 1/5 serial dilution starting from a top concentration of 20 μM. The kinase reaction was stopped after the addition of an equal volume of 150 mM phosphoric acid. ^33^P that had not been incorporated was removed by loading the samples on a filter plate (UniFilter-96 GF/B, PerkinElmer NV, BE), followed by six subsequent washing steps with 75 mM phosphoric acid. Incorporated ^33^P in the substrate was measured on a TopCount reader after the addition of MicroScint-20 (PerkinElmer NV, BE) to the filter plates.

Specific conditions for each kinase are listed in Table 10.

**Table 10:** Conditions for Radioactive Kinase Assays

enzyme, concentration	substrate, concentration	ATP and [^33^P]-γ-ATP	reaction medium
ABL1, 0.044 ng/μL	poly(Glu, Tyr), 5 μg/mL	0.5 μM ATP and 0.25 μCi [^33^P]-γ-ATP	50 mM Tris pH 7.7, 0.03% Triton X-100, 25 mM MgCl_2_, and 1 mM DTT
ALK5, 0.08 ng/μL	casein, 25 to 100 μg/mL	0.5 μM ATP and 0.25 μCi [^33^P]-γ-ATP	50 mM Tris pH 7.2, 0.01% Triton X-100, 3 mM MnCl_2_, and 2.5 mM DTT
AMPKα1/β2/γ1, 0.008 ng/μL	SAMStide, 17.8 μg/mL	125 μM 5′-AMP, 5 μM ATP and 0.25 μCi [^33^P]-γ-ATP	25 mM Tris pH 7.5, 0.5 mM EGTA, 0.01% Triton X-100, 10 mM MgCl_2_, and 2.5 mM DTT
FMS, 0.064 ng/μL	poly(Glu, Tyr), 500 μg/mL	10 μM ATP and 0.25 μCi [^33^P]-γ-ATP	50 mM Tris pH 7.0, 0.01% Triton X-100, 10 mM MgCl_2_, and 5 mM DTT
hs-LynA, 0.08 to 0.04 ng/μL	poly(Glu, Tyr), 1000 μg/mL	5 μM ATP and 0.25 μCi [^33^P]-γ-ATP	50 mM Tris pH 7.5, 0.01% Triton X-100, 10 mM MgCl_2_, and 2.5 mM DTT
TGFβR2, 1.6 to 2.4 ng/μL	none (autophosphorylation)	0.25 μM ATP and 0.125 μCi [^33^P]-γ-ATP	25 mM Tris pH 7.5, 0.5 mM EGTA, 50 mM NaCl, 0.01% Triton X-100, 5 mM MgCl_2_, and 2.5 mM DTT

### Mouse Pharmacokinetics

A total of nine male CD1 mice were given compounds via either a single intravenous bolus at 1 mg/kg or oral administration at 5 or 15 mg/kg to assess absolute bioavailability. One group of six mice was dosed intravenously with a dose level of 1 mg/kg, and one group of three mice was dosed orally via a single gavage with a dose level of 5 or 15 mg/kg. The mice were fasted before oral administration. For the iv route, the compound was formulated as a solution in polyethylene glycol (PEG) 200 and water for injection (60/40; v/v). For the oral route, the compound was formulated as a homogeneous suspension in Solutol/methyl cellulose (MC) 0.5% (2/98; v/v). Blood was sampled under light gaseous anesthesia into polypropylene tubes containing lithium heparin, and plasma was prepared. The compound was quantified in plasma using LC–MS/MS. Pharmacokinetic parameters were calculated using Phoenix software (Certara, version 6.4.0.768).

### Rat Pharmacokinetics

A total of six male Sprague–Dawley rats were given **28** either as a single intravenous bolus at 1 mg/kg or as an oral administration at 5 mg/kg to assess absolute bioavailability. One group of three rats was dosed intravenously with **28** at a dose level of 1 mg/kg, and one group of three rats was dosed orally with **28** via a single gavage with a dose level of 5 mg/kg. The rats were fasted before oral administration. For the iv route, **28** was formulated in polyethylene glycol (PEG) 200 and water for injection (60/40; v/v). For the oral route, **28** was formulated in Solutol/MC 0.5% (2/98; v/v). Blood was sampled under light gaseous anesthesia into polypropylene tubes containing lithium heparin, and plasma was prepared. **28** was quantified in plasma using LC–MS/MS. Pharmacokinetic parameters were calculated using Phoenix software (Certara, version 6.4.0.768).

### Dog Pharmacokinetics

One group of three male Beagle dogs was dosed intravenously via a 10 min infusion of **28** with a dose level of 1 mg/kg. After a washout period of 3 days, the same three dogs were dosed orally with **28** via a single gavage with a dose level of 5 mg/kg and, after a washout period of 5 days, were dosed orally via a single gavage with a dose level of 30 mg/kg. For the iv route, **28** was formulated in PEG 200 and H_2_O for injection (60/40; v/v). For the oral route, **28** was formulated in Solutol/MC 0.5% (2/98; v/v). The dogs were fasted before intravenous and oral administration. Blood was sampled without anesthetic from a jugular vein into lithium heparin tubes. Plasma was prepared, and **28** was quantified using LC–MS/MS. Pharmacokinetic parameters were calculated using Phoenix software (Certara, version 6.4.0.768).

### *In Vitro* LPS-Triggered Human Primary Monocyte Assay

The activity of **28** was evaluated on LPS-stimulated cytokine production in monocytes. Peripheral blood mononuclear cells (PBMCs) were first isolated from blood using Lymphoprep-based separation, a method which is based on the lower buoyant density of mononuclear cells (monocytes and lymphocytes) compared with other blood cell types such as erythrocytes and polymorphonuclear leukocytes (granulocytes). From these PBMCs, CD14+ monocytes were selected using antibody-coated magnetic beads (Miltenyi Biotec, DE). CD14+ monocytes were seeded in 96-well plates and preincubated with a serial dilution of **28** for 1 h before LPS triggering (Sigma-Aldrich; 100 ng/mL final concentration). TNFα and IL-10 were measured in the supernatant after 4 h of LPS triggering using enzyme-linked immunosorbent assay (ELISA)-based readouts.

### *In Vitro* LPS-Triggered Human Primary MdM Assay

To evaluate **28** in MdM, CD14^+^ monocytes (isolated as described above) were further differentiated toward macrophages using macrophage-colony stimulating factor (M-CSF [ImmunoTools]); 100 ng/mL final concentration) over 10 days. Differentiated MdM were preincubated with a serial dilution of **28** for 1 h before LPS triggering (100 ng/mL final concentration). The supernatant was collected at 2 h for IL-10 and 20 h for TNFα after LPS triggering, and cytokine levels were measured by using ELISA-based readouts.

### *In Vivo* Mouse LPS Challenge

**28** was prepared in Solutol/MC 0.5% (2/98; v/v) the day before administration and gently mixed at rt in the dark overnight. Then, it was administered orally to Balb/c mice at 0.3, 1, and 3 mg/kg. Fifteen minutes later (corresponding to the *T*_max_ of the pharmacokinetics of **28**), 100 μg of LPS (in 0.2 mL of H_2_O) was injected intraperitoneally to mice. A control group was included with Solutol/MC 0.5% (2/98; v/v) p.o. without LPS challenge. Mice were sacrificed 1.5 h after LPS challenge, and blood was collected by carotid exsanguination in heparinized tubes. Plasma samples were obtained by centrifugation for 15 min and 2000*g* at +4 °C and frozen at −80 °C before cytokine quantifications. IL-10 and TNFα were quantified by AlphaLISA according to the manufacturer’s instructions. Optical densities were determined using EnVision (PerkinElmer).

Statistical analysis was performed on raw data or log-transformed data. The normality of residuals and the equality of variances for a parametric analysis were checked. Means were compared by one-way ANOVA and Dunnett’s post hoc test. Statistical analyses were done versus the LPS/vehicle group (*** *p* < 0.001; ** *p* < 0.01; and * *p* < 0.05).

### SIK3 Kinase–UBA Preparation, Crystallization, and Structure Determination

The coding sequence from amino acids 60–394 of the human SIK3 protein (ref seq: NM_025164.6 and UniProtKB/Swiss-Prot Q9Y2K2-5) was mutated on residue 221 (T221D) and cloned into pFastBac1 with *N*-ter 6 histidine (6His) affinity tag and thrombin (Thr) protease sites giving pFastBac-6His-Thr-hsSIK3[60–394]T221D. The choice to remove the first 59 residues was motivated by the fact that this disordered sequence is not found in mouse and rat SIK3 orthologues nor human SIK1 and SIK2 proteins. The protein was expressed in the same manner as for full-length SIK3. For cell lysis, the pellet was resuspended in 25 mM Tris-HCl pH 8.0, 250 mM NaCl, 2 mM MgCl_2_, 25 mM imidazole, 1 mM DTT, and 10% v/v glycerol and was supplemented with two EDTA-free protease inhibitor cocktail tablets. The whole cell lysate was incubated at 4 °C for 1 h. Cells were lysed by sonication, and the lysate was cleared by centrifugation. For IMAC affinity chromatography, the soluble fraction was added to Ni-NTA resin (2.4 mL resin/200 mL lysate) and batch-bound overnight at 4 °C with rotation. Protein was eluted from the resin with a 25 mM–1 M imidazole gradient. Eluate samples were applied to an S200 size exclusion column, equilibrated in 25 mM Tris-HCl pH 8.0, 300 mM NaCl, 2 mM MgCl_2,_ and 0.5 mM Tris(hydroxypropyl)phosphine (THP). Peak fractions were pooled and concentrated to an ∼3 mL volume. Compound **22** described above was added at a final concentration of 20 μM, and the solution was left on ice overnight. The protein complex was concentrated further to 5 mg/mL for crystallization.

Crystals were grown at 9 °C in 12% (w/v) PEG 3350 and 0.1 M sodium citrate pH 7.0. Crystals were transferred to a solution containing mother liquor supplemented with 20% (v/v) glycerol as a cryoprotectant prior to cryocooling in liquid nitrogen. Data sets were collected by using a Dectris Pilatus 6 M detector on beamline i03 at the DLS synchrotron (Table 11). Data were indexed, integrated, and scaled using MOSFLM and AIMLESS (CCP4). MARK2 coordinates were downloaded (PDB ID: 2R0I), and Chain A was prepared for molecular replacement in PHASER (CCP4). A single solution was found by PHASER containing two SIK3 molecules per asymmetric unit. The protein sequence was mutated to match that of SIK3 by using CHAINSAW (CCP4), and the model was improved iteratively through successive cycles of model building and refinement. The molecular structure of **22** and refinement library files were produced using JLigand (CCP4). The ligand was fitted into the difference electron density in the ATP pocket by using COOT and refined by using REFMAC5 (CCP4).

**Table 11:** X-ray Crystallographic Data for Compound **22**

**data collection and processing statistics**	
X-ray source	beamline i03^a^
wavelength [Å]	0.9763
detector	Dectris Pilatus 6M
temperature [K]	100
space group	*P*6_5_22
cell: a; b; c; [Å]	174.55, 174.55, 152.31
→α; β; γ; [◦]	90.0, 90.0, 120.0
resolution range [Å]^b^	107.29–3.10 (3.31–3.10)
unique reflections	25156 (4504)
multiplicity	9.6 (9.6)
completeness [ %]	99.3 (99.6)
*R*_sym_ [%]^c^	21.8 (288.7)
*R*_meas_ [%]^d^	24.1 (322.1)
mean(I)/sd^e^	7.2 (0.90)
*R*_work_	0.22
*R*_free_	0.26
mean overall *B* value [Å^2^]	96.0
Ramachandran favored [%]	97
Ramachandran allowed [%]	3
Ramachandran outlier [%]	0

a Diamond Light Source (Oxfordshire, United Kingdom).

b Values in parentheses refer to the highest resolution bin.

c where *I*_*h,i*_ is the intensity value of the *i*th measurement of *h*

d where *I*_*h,i*_ is the intensity value of the *i*th measurement of *h*

e Calculated from independent reflections.

### Molecular Modeling

All molecular modeling calculations were carried out using the Schrödinger software suite (Schrödinger Release 2018–1, Schrödinger, LLC, New York, USA).

### Ligand Docking

All docked compounds were built and protonated using LigPrep,^31^ whereas ionization states at pH between 5 and 9 were calculated with Epik.^32^ The crystal structure of AMPK1 was taken from the RCSB protein databank (PDB code: 4RER(33)).

For both AMPK1 and the internal SIK3 X-ray structure, hydrogen atoms were added to the protein through the Protein Preparation Wizard tool.^34^ In order to optimize the hydrogen bond network, the most putative protonation state of the residues was carefully selected by visual inspection, and hydrogen atoms were minimized using the OPLS3 force field.^35^ Before the docking procedure was run, all water molecules present in the PDB were removed.

Docking of the ligands was carried out with Glide.^36^ A docking grid was generated using AMPK1 or SIK3 prepared structures. The cocrystallized ligand was selected as the center of the grid, and a hydrogen bond constraint with the hinge hydrogen bond donor (Ala145 NH for SIK3 and Val98 NH for AMPK1) was created, whereas the rest of the settings were kept as default. These constraints were used as these two hydrogen bond interactions are the main ones fixing the scaffold close to the hinge. For the docking run, the flexible docking standard precision option was selected together with an enhanced sampling protocol (four times) of the ligands. The constraint was applied to all docking runs, whereas the number of poses to return was set as three for each ligand.

The binding modes were then selected based on spatial geometries of the ligand within the binding cavity and complementarity with the pocket (shape and electrostatic complementarity).

## Supporting Information Available

Individual percentage of kinase inhibition by **28** at 1 and 0.1 μM; crystal structure; and NMR spectra and HPLC traces of the synthesized compounds (PDF)
Molecular formula strings with associated data (CSV)

## Accession Codes

The crystal structure reported here has been deposited in the Protein Data Bank with the following accession code: 8OKU. Authors will release the atomic coordinates and experimental data upon article publication.

## Abbreviations

dd: doublet of doublets
ddd: doublet of doublet of doublets
dq: doublet of quartets
dt: doublet of triplets
dtd: doublet of triplet of doublets
dioxane: 1,4-dioxane
DIPEA: *N*,*N*-diisopropylethylamine
Et_2_O: diethyl ether
EtOAc: ethyl acetate
EtOH: ethanol
HATU: hexafluorophosphate azabenzotriazole tetramethyl uronium
HOBt: hydroxybenzotriazole
IBD: inflammatory bowel disease
iPrOH: isopropanol
LHMDS: lithium hexamethyldisilazane
MC: methyl cellulose
MeCN: acetonitrile
MeOH: methanol
*n*-BuOH: *n*-Butanol
PBMC: peripheral blood mononuclear cell
ND: not determined
Pd(PPh_3_)_4_: tetrakis(triphenylphosphine)palladium(0)
Pd(dppf)Cl_2_.DCM: [1,1′-bis(diphenylphosphino)ferrocene]dichloropalladium(II) (1:1)
PTSA: *para*-toluenesulfonic acid
RA: rheumatoid arthritis
THP: tris(hydroxypropyl)phosphine
