# Supplementary material for: Optimization of Selectivity and Pharmacokinetic Properties of Salt-Inducible Kinase Inhibitors that Led to the Discovery of Pan-SIK Inhibitor GLPG3312
Source: J Med Chem. 2023 Dec 26;67(1):380–401. doi: 10.1021/acs.jmedchem.3c01428 (PMC10788895; doi:10.1021/acs.jmedchem.3c01428)

Supporting Information

Optimization of Selectivity and Pharmacokinetic Properties Of Salt-Inducible Kinase Inhibitors that Led to the Discovery Of Pan-SIK Inhibitor GLPG3312

Taouès Temal-Laib,[1] Christophe Peixoto,[1]* Nicolas Desroy,[1]* Elsa De Lemos,[1] Florence Bonnaterre,[1] Natacha Bienvenu,[1] Olivier Picolet,[1] Eric Sartori,[1] Denis Bucher,[1] Miriam López-Ramos,[1] Carlos Roca Magadán,[1] Wendy Laenen,[2] Thomas Flower,[1] Patrick Mollat,[1] Olivier Bugaud,[1] Robert Touitou,[1] Anna Pereira Fernandes,[2] Stephanie Lavazais,[1] Alain Monjardet,[1] Monica Borgonovi,[1] Romain Gosmini,[1] Reginald Brys,[2] David Amantini,[1] Steve De Vos,[2] and Martin Andrews[2]

*Corresponding authors

[1]Galapagos SASU, 93230 Romainville, France

[2]Galapagos NV, 2800 Mechelen, Belgium

Christophe Peixoto – NovAliX, 102 Avenue Gaston Roussel, 93230 Romainville, France; Email: cpeixoto@novalix.com; ORCID: 0009-0007-9365-9275

Nicolas Desroy – Galapagos SASU, 102 Avenue Gaston Roussel, 93230 Romainville, France; Email: nicolas.desroy@glpg.com; ORCID: 0000-0002-4879-3439

Contents

Table S1. 2

Supplementary Figures 11

LCMS traces 13

NMR traces 56

Table S1. Percentage of Kinase Inhibition by 28 at 1 μM (380 Kinases) and 0.1 μM (360 Kinases), Assays Performed at Eurofins

Kinase name	Percentage of inhibition	
	1 μM	0.1 μM
AAK1	-21	-11
ABL1	74	37
ABL2	71	30
ACVR1	60	10
ACVR1B	49	-6
ACVR2A	62	11
ACVRL1	51	22
AKT1	-6	6
AKT2	-12	1
AKT3	-2	-6
ALK	4	30
ARAF	5	0
ATM	1	6
ATR	-3	-5
AURKA	4	11
AURKB	26	1
AURKC	6	38
AXL	21	-3
BLK	72	7
BMP2K	-10	18
BMPR1B	52	-3
BMPR2	14	-8
BMX	18	15
BRAF	18	4
BRSK1	-4	3
BRSK2	7	10
BTK	0	-3
CAMK1	-5	1
CAMK1D	0	3
CAMK1G	-5	5
CAMK2A	2	9
CAMK2B	2	7
CAMK2D	9	-5
CAMK2G	-10	2
CAMK4	-3	-7
CAMKK1	-1	-1
CAMKK2	-10	10
CDC42BPA	0	5
CDC42BPB	8	2
CDC42BPG	4	1
Cdc7/cyclinB1	-11	9
CDK1/CCNB1	-1	-1

CDK14/CCNY	1	-4
CDK16/CCNY	0	-6
CDK17/CCNY	12	-9
CDK18/CCNY	-10	12
CDK2/CCNA2	2	0
CDK2/CCNE1	-2	-2
CDK3/CCNE1	-5	-3
CDK4/CCND3	-3	14
CDK5/p25CDK5R1	9	2
CDK5/p35CDK5R1	-2	9
CDK6/CCND3	4	-38
CDK7/CCNH/MNAT1	4	-15
CDK9/CCNT1	2	1
CDKL1	0	27
CDKL2	-25	11
CDKL3	1	0
CDKL4	3	-4
CHEK1	-10	17
CHEK2	5	13
CHUK	4	5
CIT	-1	11
CLK1	22	5
CLK2	3	15
CLK3	4	19
CLK4	-2	15
CSF1R (FMS)	69	15
CSK	2	17
CSNK1A1	-7	4
CSNK1D	-9	-3
CSNK1E	-4	-7
CSNK1G1	8	4
CSNK1G2	-2	2
CSNK1G3	-2	14
CSNK2A1	-4	11
CSNK2A1/B	1	7
CSNK2A2	-14	0
DAPK1	13	2
DAPK2	-1	-1
DAPK3	-1	15
DCLK1	-9	11
DCLK2	10	-25
DCLK3	1	3
DDR1	96	71
DDR2	14	24
DMPK	2	10
DYRK1A	0	-4

DYRK1B	17	-9
DYRK2	-9	11
DYRK3	-13	-1
EEF2K	1	11
EGFR	-6	-7
EIF2AK1	-7	5
EIF2AK2	-1	4
EIF2AK3	-2	17
EIF2AK4	-3	10
EPHA1	29	9
EPHA2	-12	0
EPHA3	15	-13
EPHA4	5	14
EPHA5	10	-4
EPHA7	2	13
EPHA8	25	10
EPHB1	12	-4
EPHB2	0	-2
EPHB3	7	-7
EPHB4	4	-7
ERBB2	3	11
ERBB4	0	-2
ERN1	9	6
FER	3	-13
FES	5	-5
FGFR1	12	-4
FGFR2	2	6
FGFR3	8	16
FGFR4	-1	-3
FGR	46	17
FLT1	-3	-6
FLT3	-5	17
FLT3(D835Y)	-12	not tested
FLT4	3	-13
FRK	-3	5
FYN	59	22
GAK	50	not tested
GRK1	-3	6
GRK2	8	4
GRK3	5	2
GRK4	5	not tested
GRK5	-2	5
GRK6	7	-2
GRK7	1	16
GSG2	-7	-16
GSK3A	0	-2

GSK3B	3	10
HCK	53	21
HCK act	39	22
HIPK1	15	1
HIPK2	21	5
HIPK3	-9	0
HIPK4	9	-11
ICK	-8	-3
IGF1R	18	1
IGF1R act	2	-11
IKBKB	-7	-10
IKBKE	8	-7
INSR	-2	3
INSR act	-3	8
INSRR	-12	-5
IRAK1	1	-18
IRAK4	-5	8
ITK	-3	-11
JAK1	-2	-9
JAK2	18	14
JAK3	4	-5
KDR	-8	-5
KIT	55	-4
LATS1	-2	-1
LATS2	-10	-5
LCK	80	16
LCK activated	61	13
LIMK1	94	78
LIMK2	27	-2
LRRK2	11	-24
LTK	0	11
LYN	77	13
MAK	-8	21
MAP2K1	-1	-13
MAP2K2	7	5
MAP2K3	7	2
MAP2K6	7	-2
MAP3K10	6	22
MAP3K11	12	17
MAP3K2	5	8
MAP3K20	98	80
MAP3K21	1	9
MAP3K3	-1	-4
MAP3K5	-5	8
MAP3K7	2	1
MAP3K8	-6	not tested

MAP3K9	10	9
MAP4K1	19	3
MAP4K2	-2	7
MAP4K3	8	3
MAP4K4	-1	11
MAP4K5	18	0
MAPK1	11	5
MAPK10	2	5
MAPK11	30	19
MAPK12	-5	-10
MAPK13	7	16
MAPK14	9	10
MAPK3	27	15
MAPK8	0	15
MAPK9	7	2
MAPKAPK2	-5	-15
MAPKAPK3	-4	1
MAPKAPK5	-1	1
MARK1	43	-2
MARK2	23	17
MARK3	41	12
MARK4	30	14
MATK	-1	not tested
MELK	0	7
MERTK	7	-15
MET	-12	9
MET(D1246H)	1	not tested
MINK1	0	-16
MKNK1	0	not tested
MKNK2	-5	12
MOK	21	-8
MST1R	-3	15
MST4	5	-15
MTOR	1	-4
MTOR/FKBP1A	8	11
MUSK	8	11
MYLK	-3	8
MYLK2	-7	-3
MYO3B	3	8
NDRG1	3	-2
NEK1	1	-8
NEK11	1	1
NEK2	-6	-6
NEK3	-3	-11
NEK4	-2	-6
NEK5	-9	not tested

NEK6	8	15
NEK7	8	4
NEK9	0	-1
NIM1K	1	-5
NLK	66	26
NTRK1	7	-2
NTRK2	6	-10
NTRK3	10	-12
NUAK1	12	-20
NUAK2	2	22
OSR1	-1	11
PAK1	2	10
PAK2	0	-20
PAK3	1	-11
PAK4	-2	-3
PAK6	16	2
PAK7	-2	10
PASK	2	-5
PDGFRA	-4	2
PDGFRB	-1	-6
PDK2	-3	8
PDK4	-12	-4
PDPK1	11	-1
PHKG1	9	-28
PHKG2	2	9
PIK3C2A	-7	not tested
PIK3C2G	-1	not tested
PIK3CA/PIK3R1(p110a/p85a)	-4	not tested
PIK3CB/PIK3R1(p110b/p85a)	2	not tested
PIK3CD/PIK3R1(p110d/p85a)	0	not tested
PIK3CG (p110g)	-2	not tested
PIM1	1	15
PIM2	2	4
PIM3	-8	-8
PIP4K2A	0	not tested
PIP5K1A	-4	not tested
PIP5K1C	-3	not tested
PKN1	-7	2
PKN2	9	-9
PLK1	2	8
PLK2	-2	5
PLK3	-8	14
PLK4	1	2
PNCK	8	4
PRKAA1	17	11
PRKAA2	27	20

PRKACA	3	-7
PRKACB	-1	-1
PRKCA	10	-29
PRKCB1	9	-2
PRKCB2	2	5
PRKCD	5	5
PRKCE	2	9
PRKCG	4	1
PRKCH	-8	11
PRKCI	7	10
PRKCQ	17	-3
PRKCZ	5	2
PRKD1	7	9
PRKD2	7	-6
PRKD3	-4	-10
PRKDC	1	not tested
PRKG1a	-8	10
PRKG1b	8	-14
PRKG2	0	2
PRKX	-12	8
PRPF4B	1	8
PTK2	2	4
PTK2B	11	13
PTK6	8	-2
RAF1	39	7
RET	-13	-1
RIPK1	1	0
RIPK2	98	85
ROCK1	5	5
ROCK2	1	-11
ROS1	2	-1
RPS6KA1	-9	-21
RPS6KA2	-4	4
RPS6KA3	-3	8
RPS6KA4	2	1
RPS6KA5	3	1
RPS6KA6	-12	15
RPS6KB1	3	2
RPS6KB2	4	not tested
SBK1	-7	6
SGK1	-6	0
SGK2	3	10
SGKL	-2	5
SIK1	99	91
SIK2	105	104
SIK3	105	109

SLK	5	-10
SNRK	11	1
SRC	18	3
SRMS	14	-16
SRPK1	-3	-5
SRPK2	1	-12
SRPK3	2	0
STK10	12	10
STK11	3	3
STK16	-2	11
STK17A	-16	4
STK17B	-1	3
STK24	1	-22
STK25	2	13
STK3	-4	4
STK32A	-6	10
STK32B	-6	3
STK32C	4	-10
STK33	2	-4
STK35	-5	5
STK38L	-4	7
STK39	11	12
STK4	-6	-5
SYK	-6	-5
TAF1L	0	1
TAOK1	0	11
TAOK2	6	0
TAOK3	12	0
TBK1	7	-6
TEC	2	-8
TEK	5	-7
TGFBR1	29	1
TGFBR2	-3	-7
TLK1	-2	-15
TLK2	3	3
TNIK	26	-18
TNK2	47	15
TRIB2	-3	4
TRPM7	-8	8
TSSK1B	6	-17
TSSK2	11	13
TSSK3	-2	-4
TSSK4	3	-14
TTBK1	-1	-2
TTBK2	-2	-2
TTK	13	-12

TXK	13	7
TYK2	14	12
TYRO3	-5	-4
ULK1	5	6
ULK2	-5	14
ULK3	6	-2
VRK1	12	14
VRK2	-5	-7
WEE1	-3	-1
WEE2	3	4
WNK1	5	-4
WNK2	3	10
WNK3	-2	3
WNK4	-11	-6
YES1	76	27
ZAP70	-6	-5

Supplementary Figures

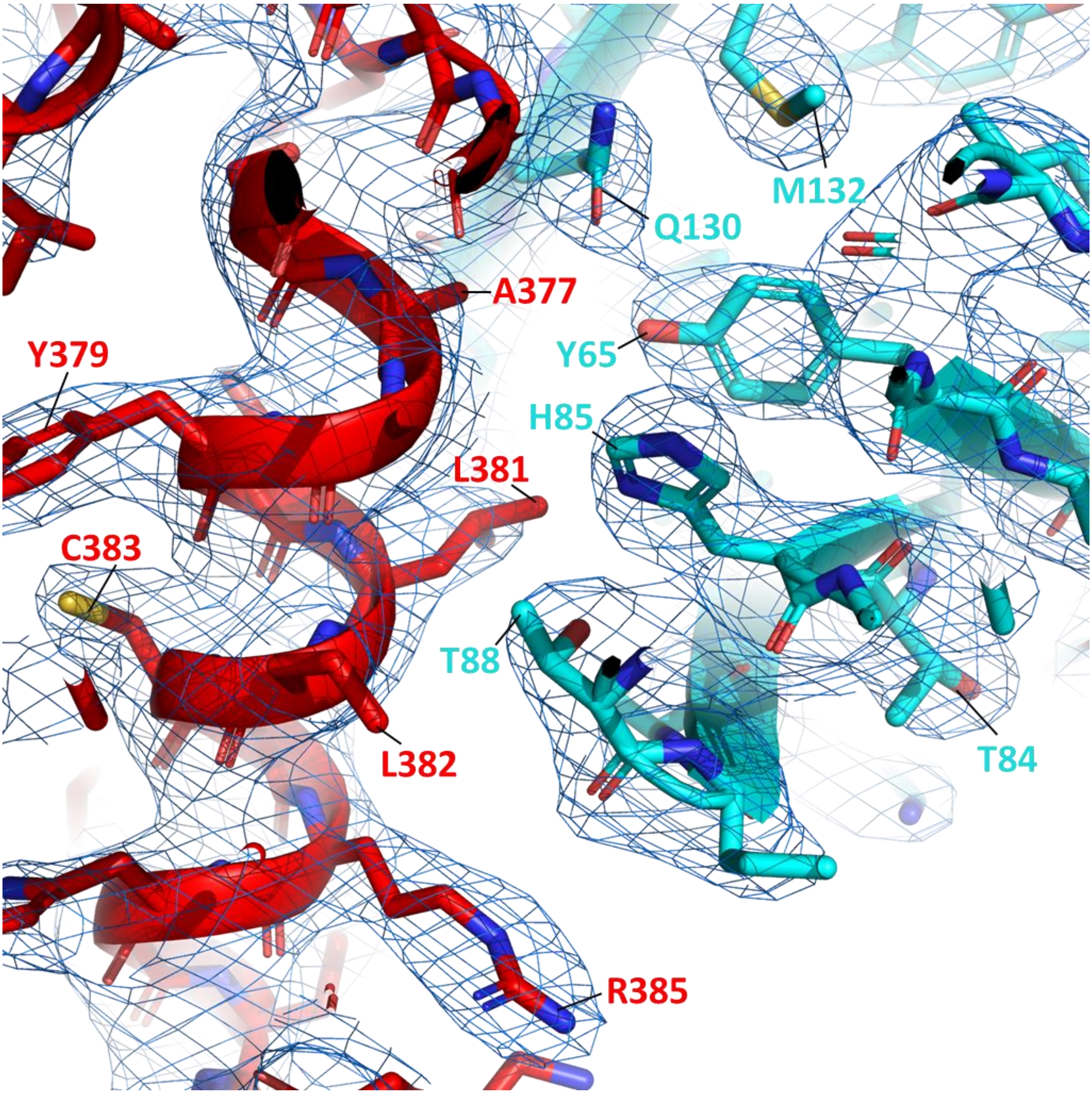

Figure S1. Interface between *N*-lobe of kinase domain and UBA domain, colored cyan and red respectively. Key residues are labeled. Electron density is shown as a blue mesh, contoured at 0.8*σ*.

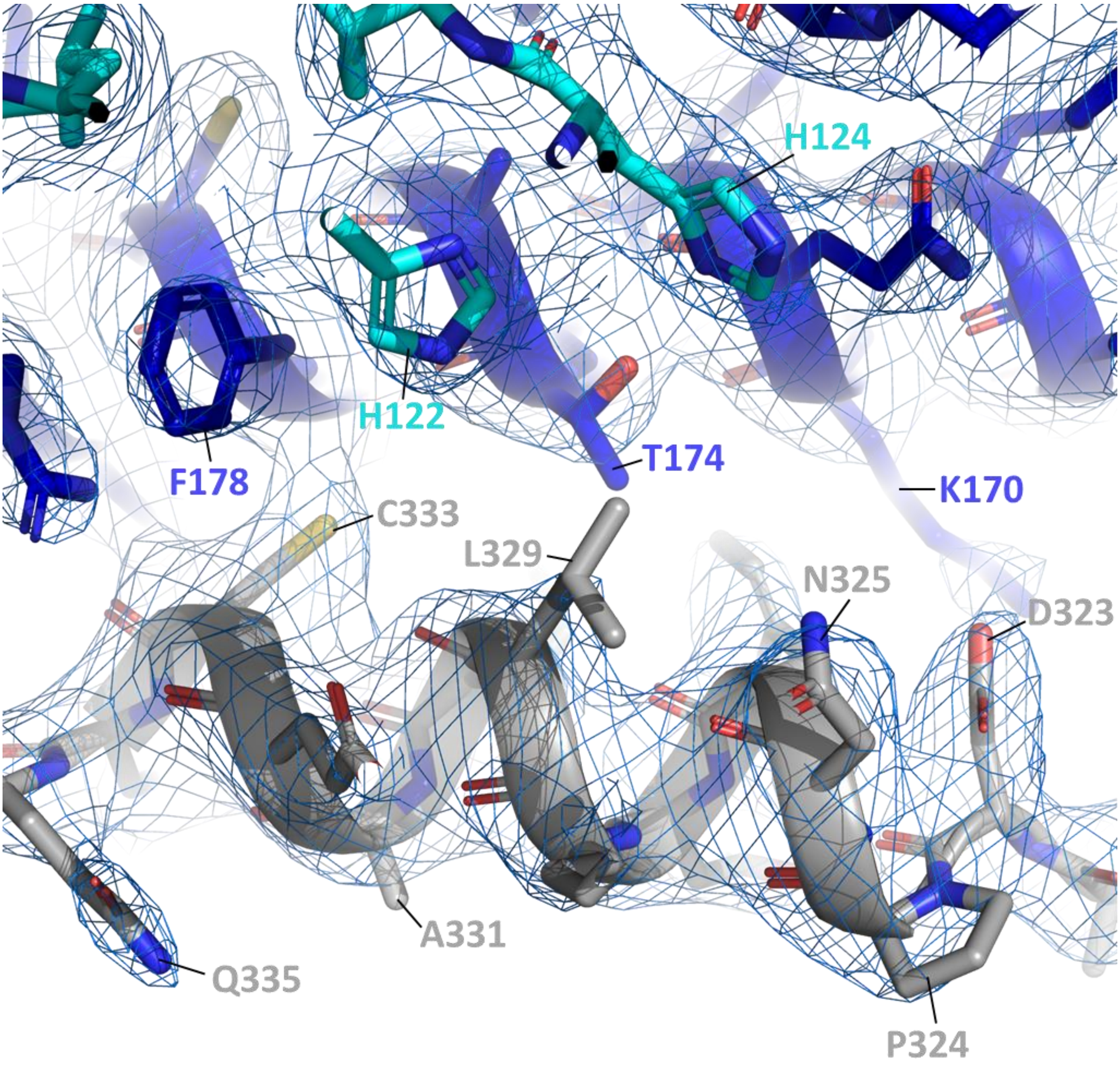

Figure S2. Interface between kinase domain and linker region connecting to the UBA domain. The kinase *N*-lobe, *C*-lobe, and linker regions are colored cyan, blue and gray respectively. Key residues are labelled. Electron density is shown as a blue mesh, contoured at 0.8*σ*.

LCMS traces

Analysis performed on Acquity UPLC systems from Waters, controlled by Masslynx software.

Compound **8**

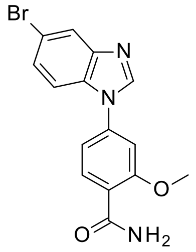

Exact Mass: 345.01
Molecular Formula: $C_{15}H_{12}BrN_3O_2$

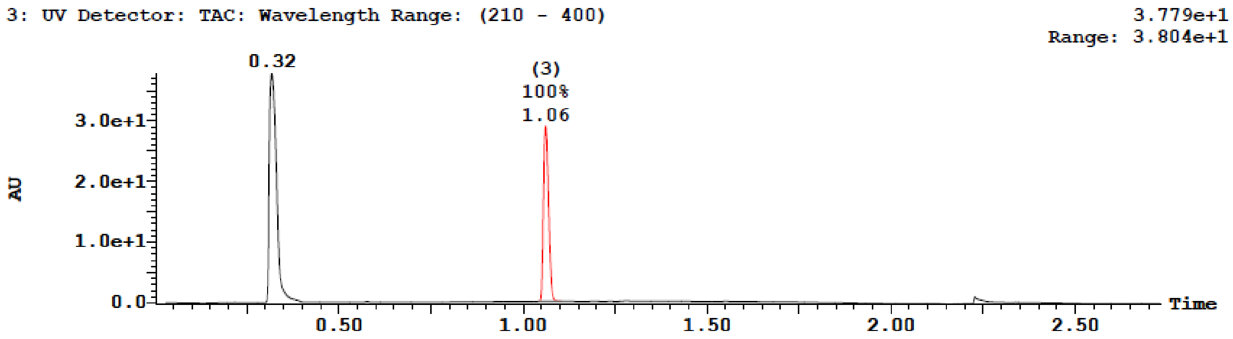

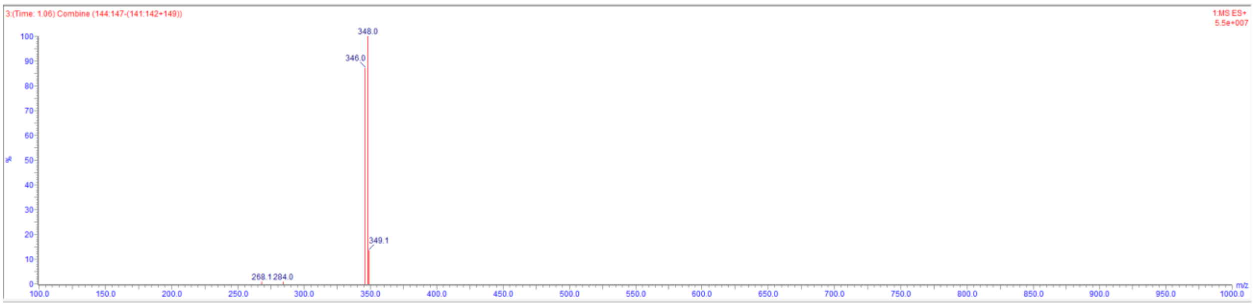

Compound **9**

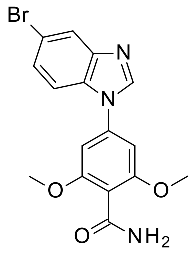

Exact Mass: 375.02
Molecular Formula: $C_{16}H_{14}BrN_3O_3$

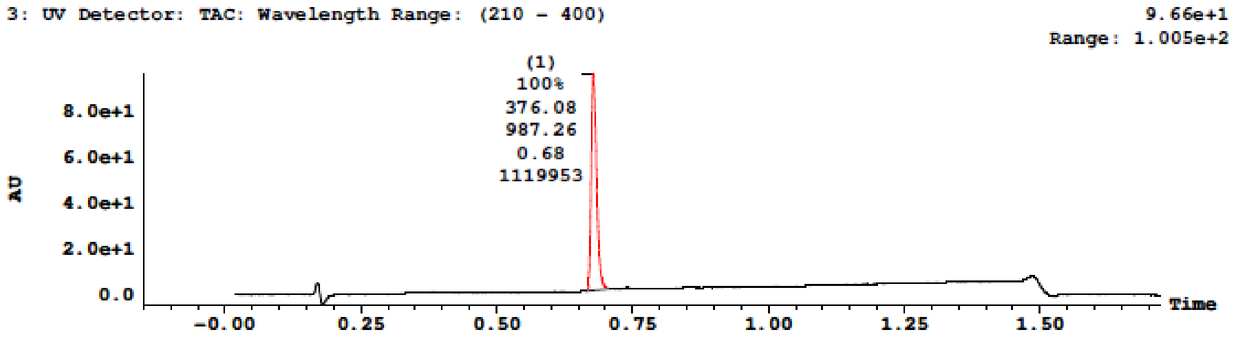

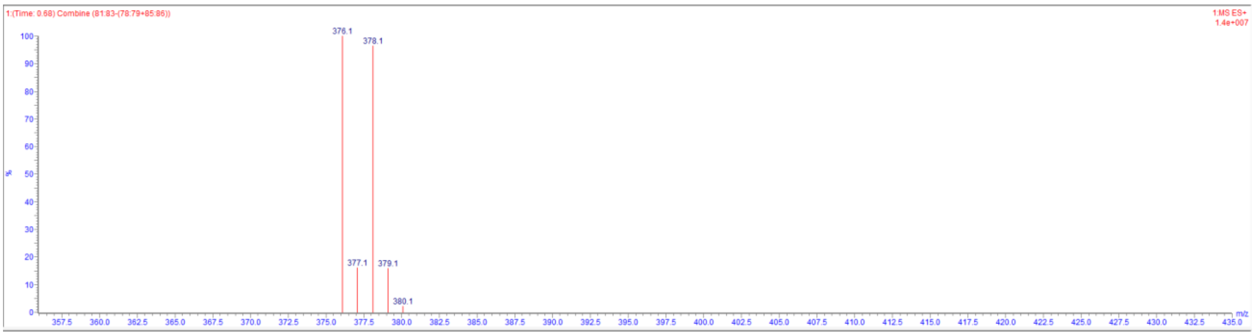

Compound **10**

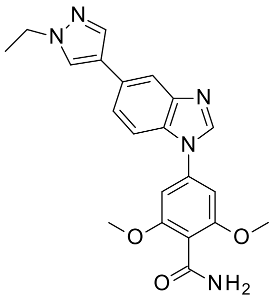

Exact Mass: 391.16
Molecular Formula: $C_{21}H_{21}N_5O_3$

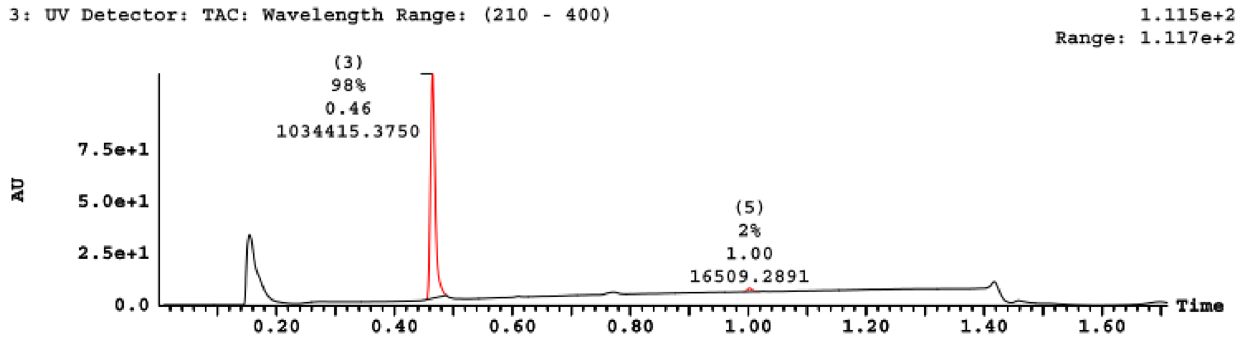

Peak ID	Compound	Time	Mass Found
3		0.46	Not Found

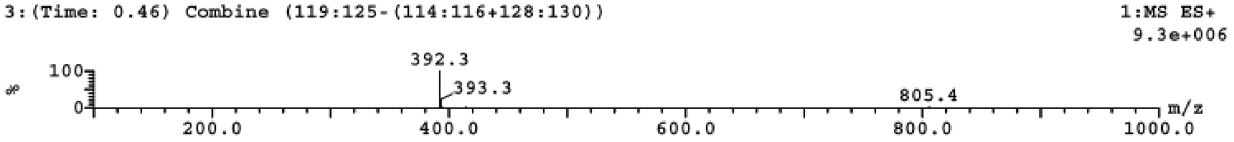

Compound **11**

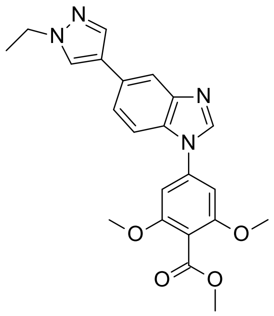

Exact Mass: 406.16
Molecular Formula: $C_{22}H_{22}N_4O_4$

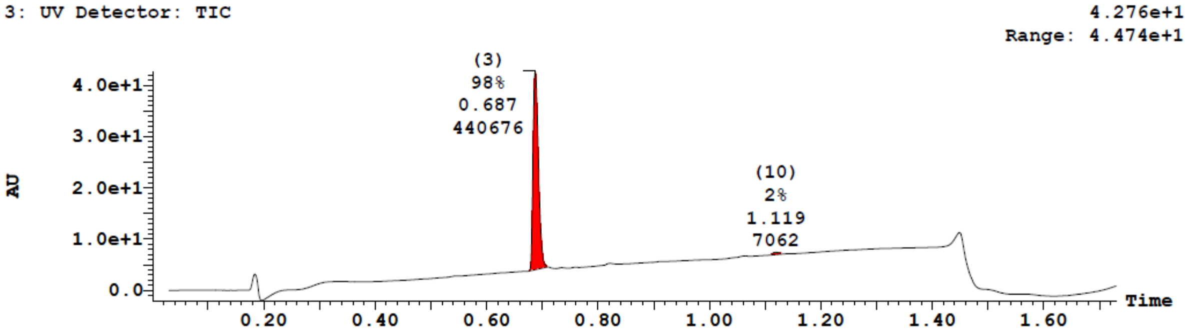

Peak Number	Time	Area %Total	Mass Found
3	0.69	98.42	
10	1.12	1.58	

Peak ID	Time	Mass Found
3	0.69	

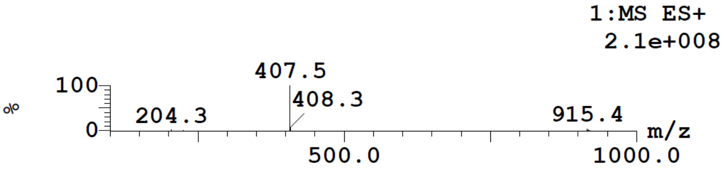

Compound **12**

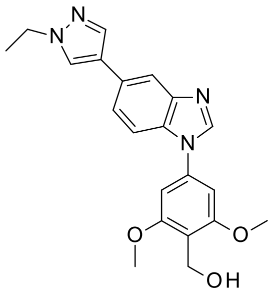

Exact Mass: 378.17
Molecular Formula: $C_{21}H_{22}N_4O_3$

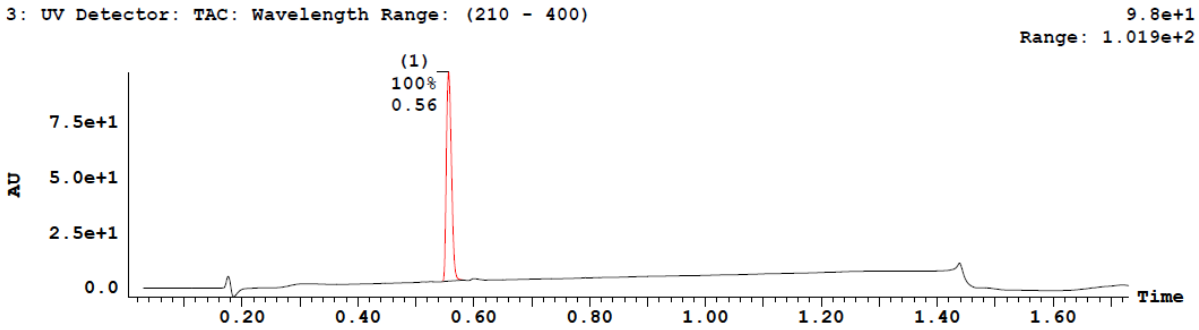

Peak ID	Mass Found	Time
1	Not Found	0.56

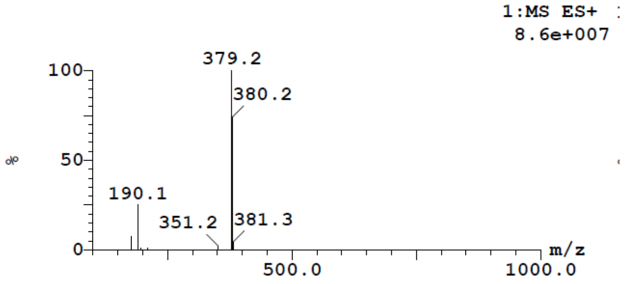

Compound **13**

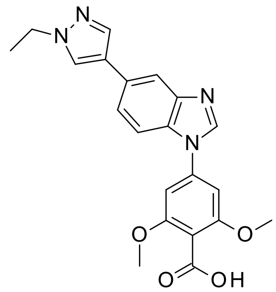

Exact Mass: 392.15
Molecular Formula: $C_{21}H_{20}N_4O_4$

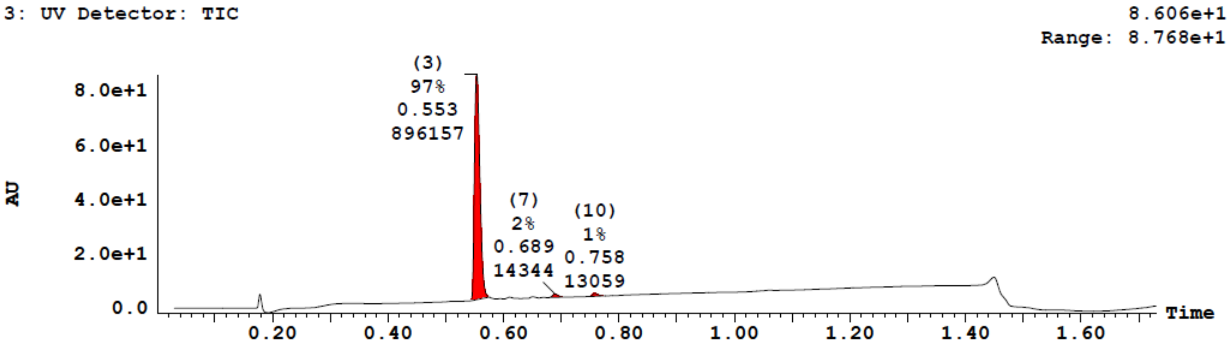

Peak ID	Time	Mass Found
3	0.55	

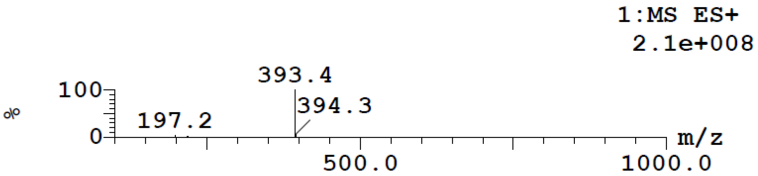

Compound **14**

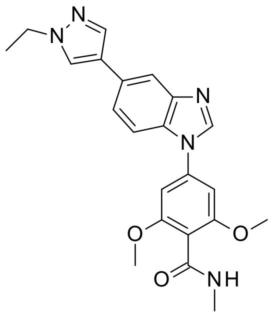

Exact Mass: 405.18
Molecular Formula: $C_{22}H_{23}N_5O_3$

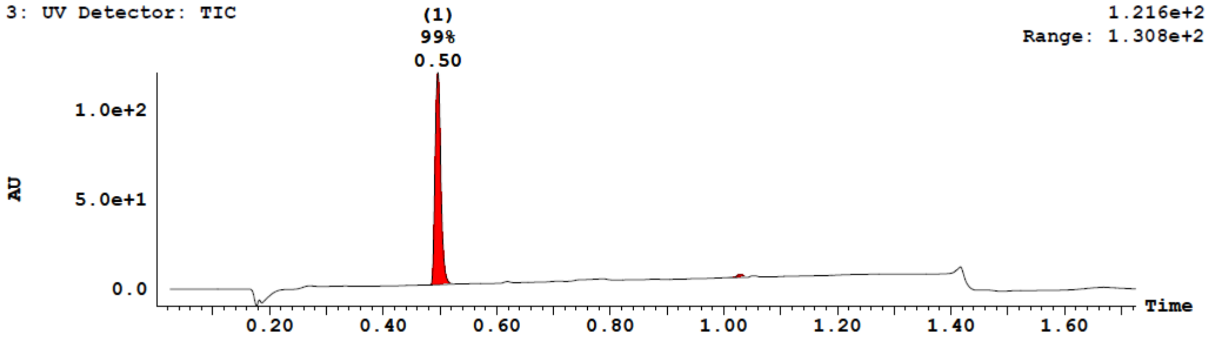

Peak ID	Time	Mass Found
1	0.50	

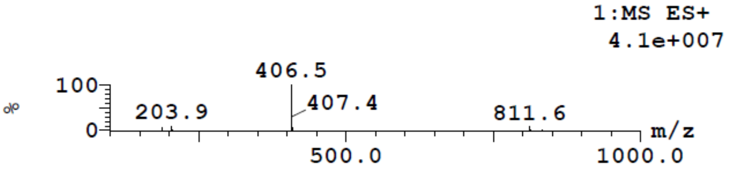

Compound **15**

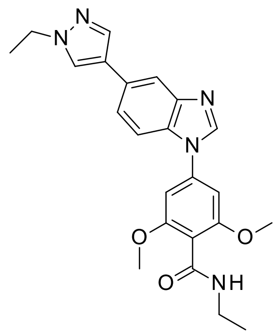

Exact Mass: 419.2
Molecular Formula: $C_{23}H_{25}N_5O_3$

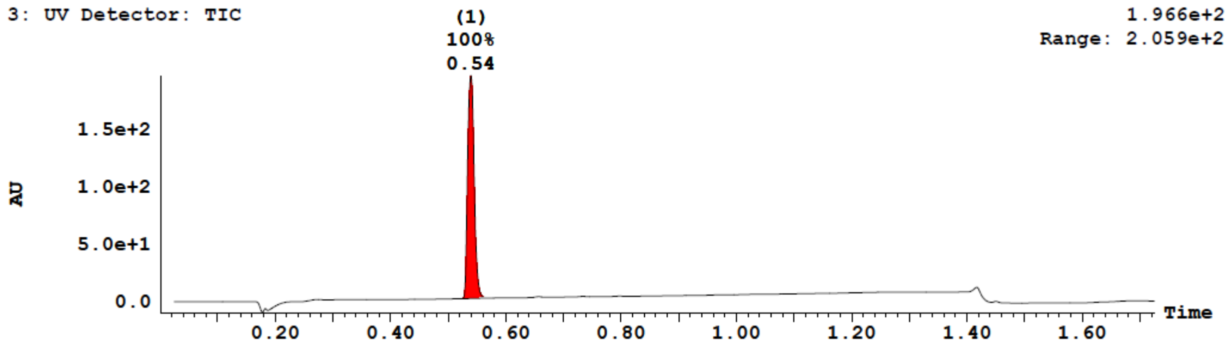

Peak ID	Time	Mass Found
1	0.54	

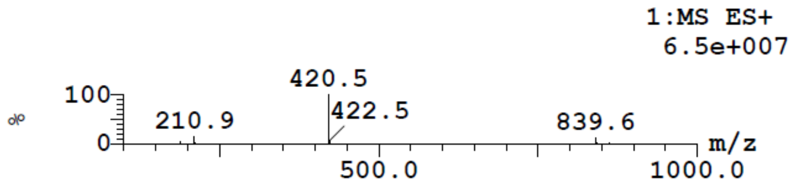

Compound **16**

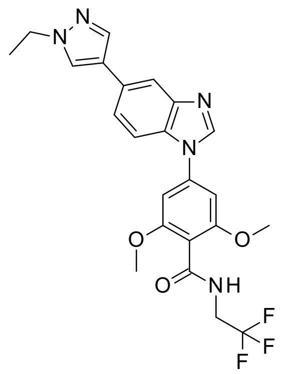

Exact Mass: 473.17
Molecular Formula: $C_{23}H_{22}F_3N_5O_3$

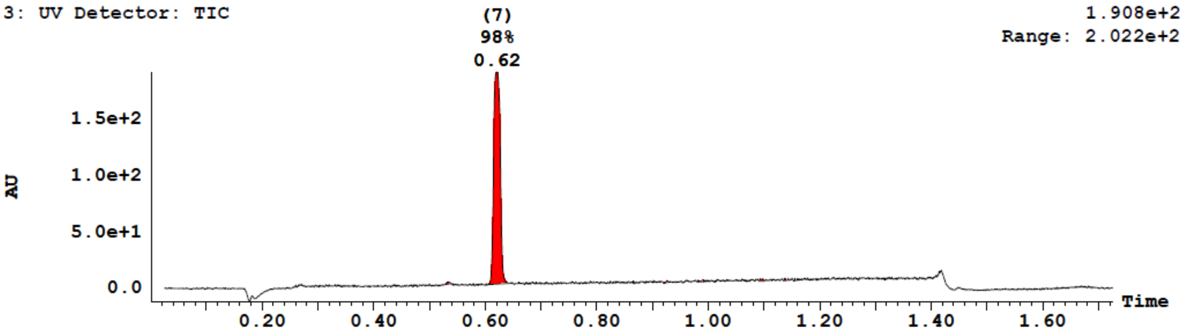

Peak ID	Time	Mass Found
7	0.62	

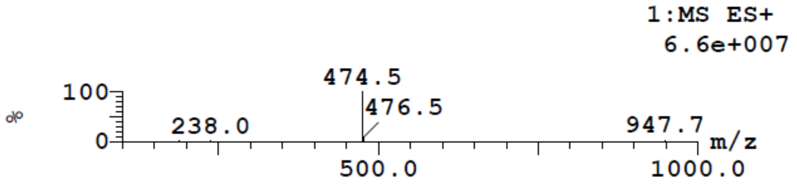

Compound **17**

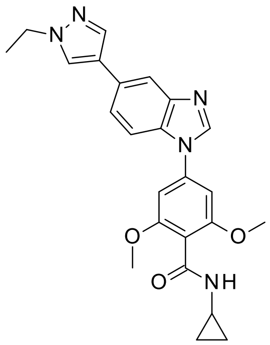

Exact Mass: 431.2
Molecular Formula: $C_{24}H_{25}N_5O_3$

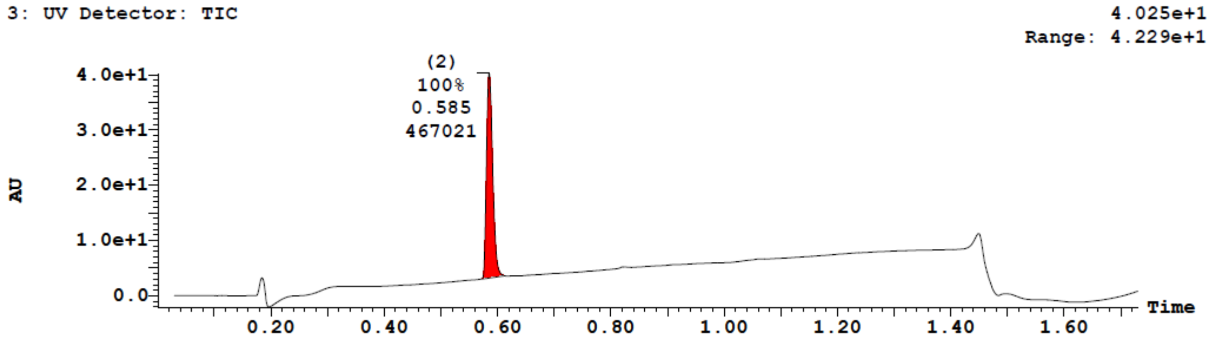

Peak ID	Time	Mass Found
2	0.58	

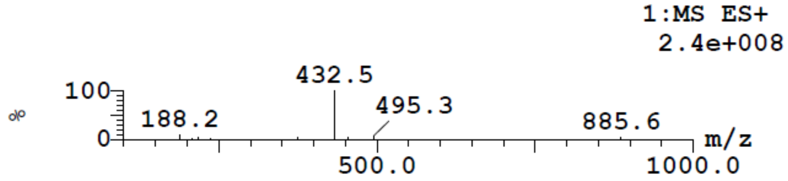

Compound **18**

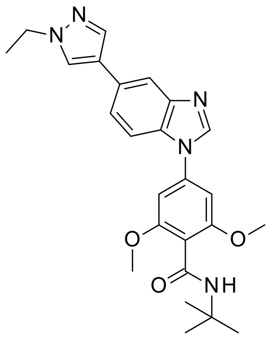

Exact Mass: 447.23
Molecular Formula: $C_{25}H_{29}N_5O_3$

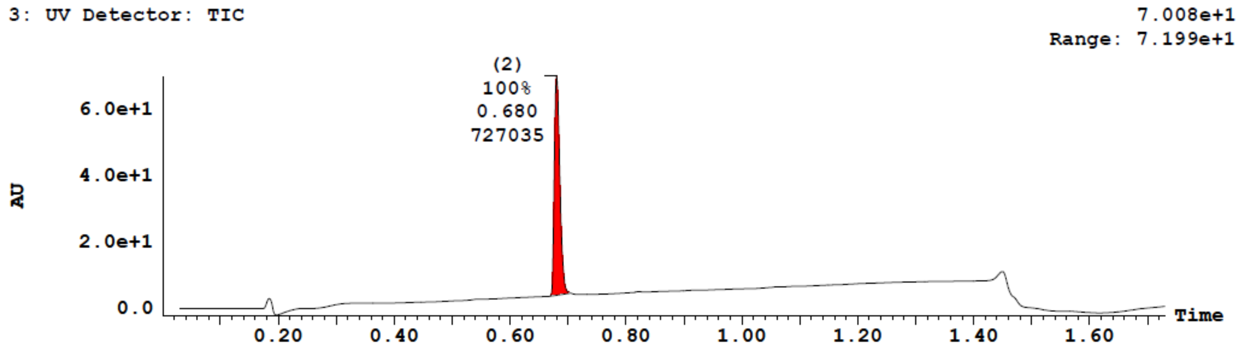

Peak ID	Time	Mass Found
2	0.68	

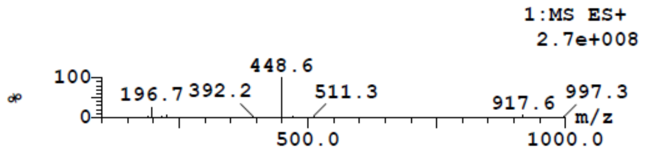

Compound **19**

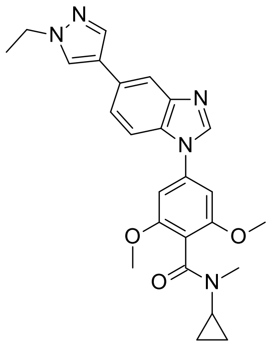

Exact Mass: 445.21
Molecular Formula: $C_{25}H_{27}N_5O_3$

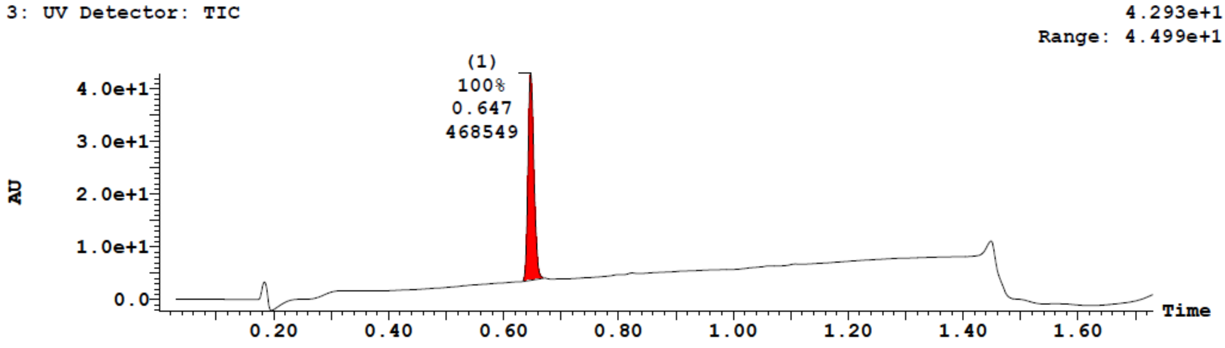

Peak ID	Time	Mass Found
1	0.64	

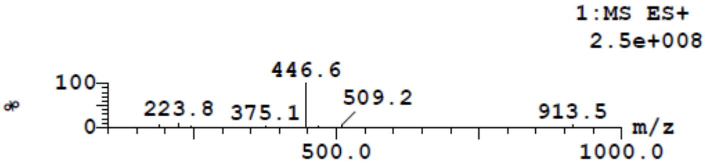

Compound **20**

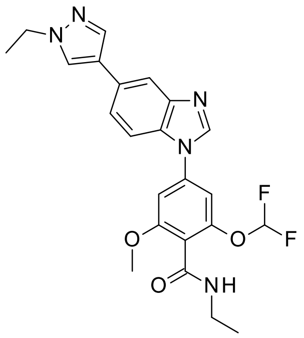

Exact Mass: 455.18
Molecular Formula: $C_{23}H_{23}F_2N_5O_3$

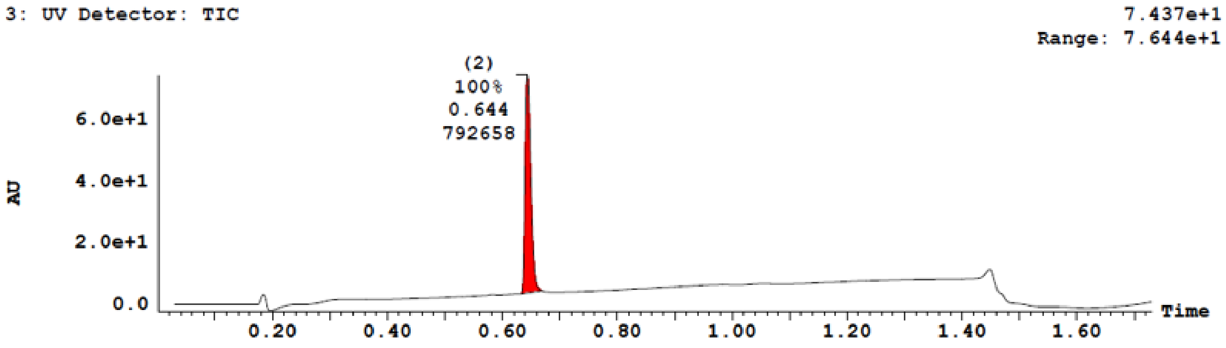

Peak ID	Time	Mass Found
2	0.64	

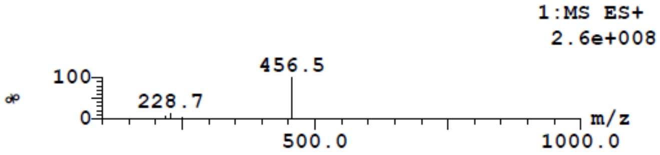

Compound **21**

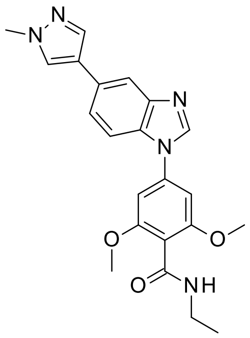

Exact Mass: 405.18
Molecular Formula: $C_{22}H_{23}N_5O_3$

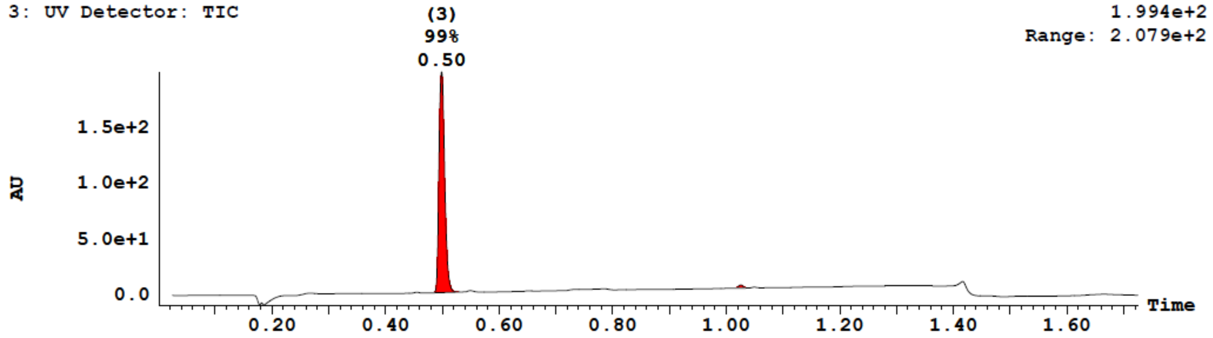

Peak ID	Time	Mass Found
3	0.50	

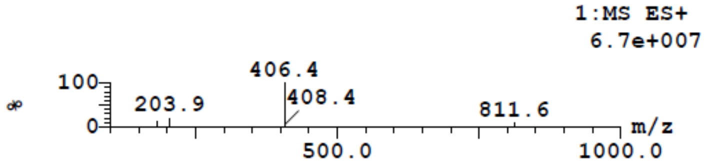

Compound **22**

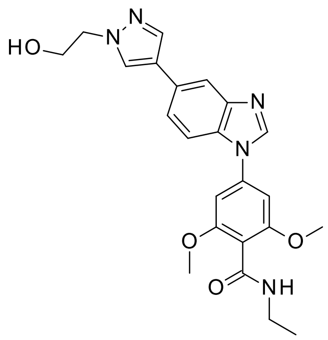

Exact Mass: 435.19
Molecular Formula: $C_{23}H_{25}N_5O_4$

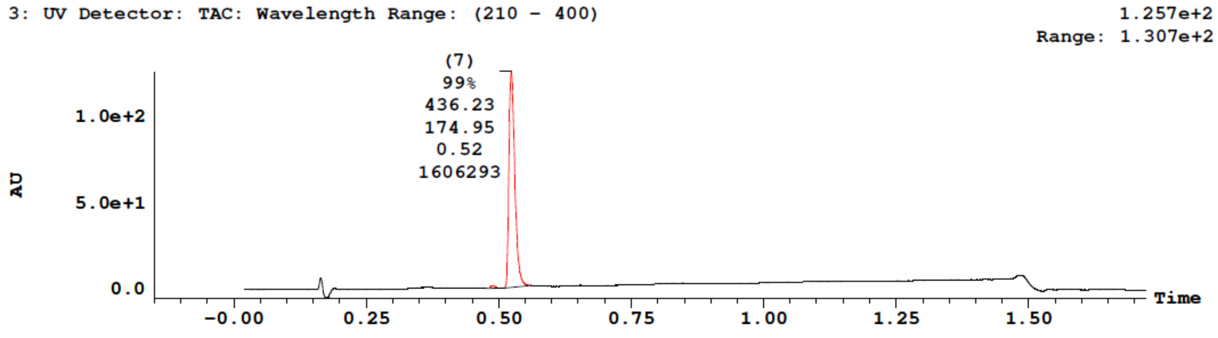

Peak ID	Mass Found	Time
7	Not Found	0.53

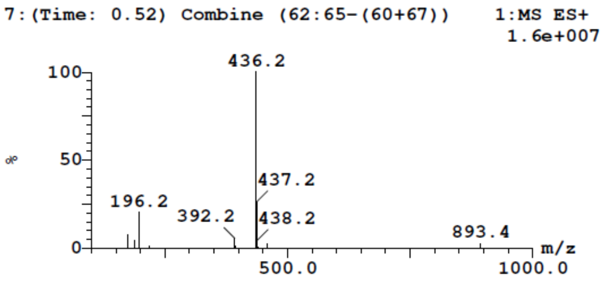

Compound **23**

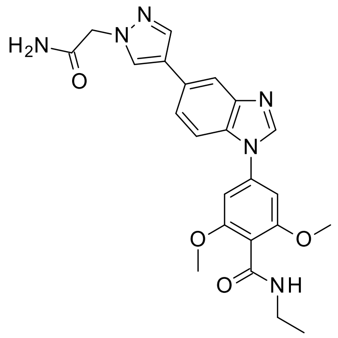

Exact Mass: 448.19
Molecular Formula: $C_{23}H_{24}N_6O_4$

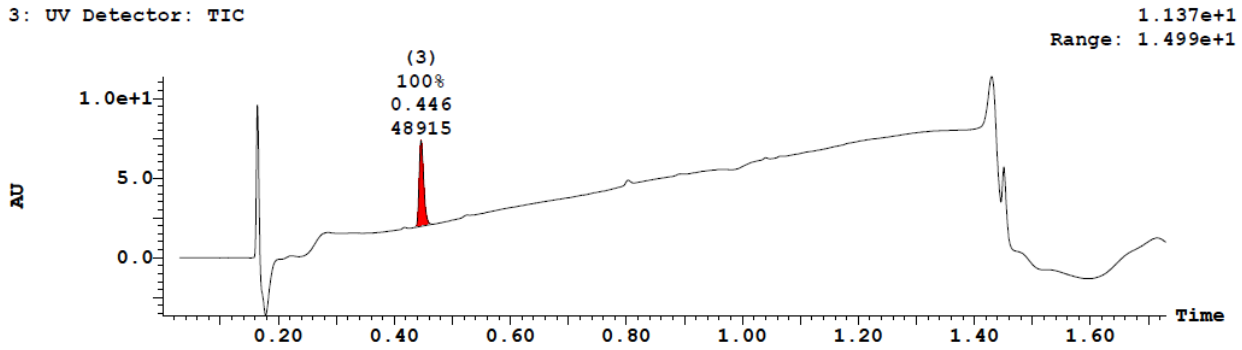

Peak ID	Time	Mass Found
3	0.45	

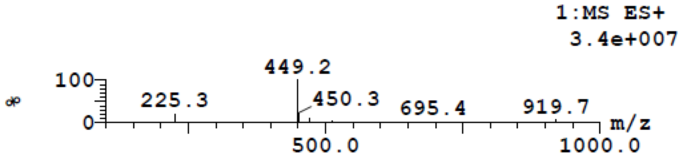

Compound **24**

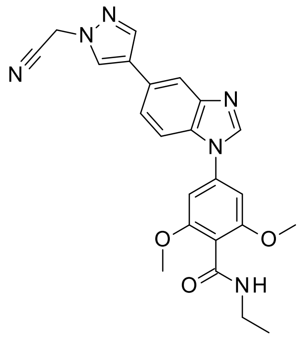

Exact Mass: 430.18
Molecular Formula: $C_{23}H_{22}N_6O_3$

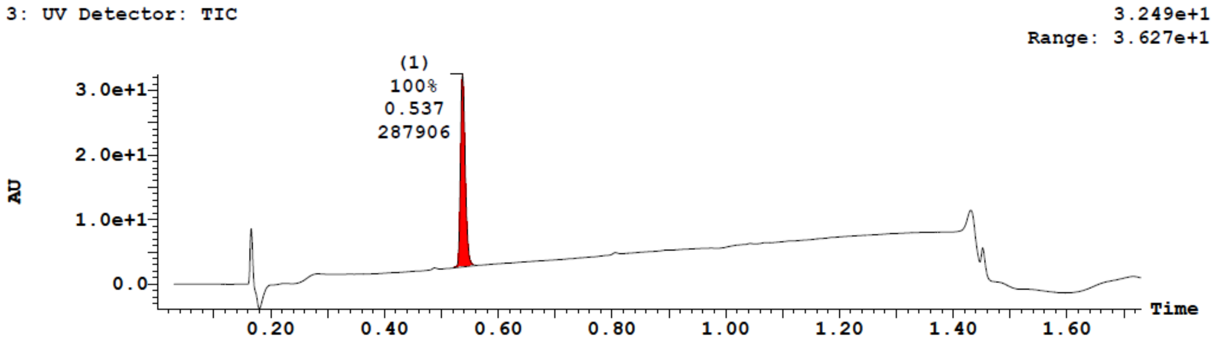

Peak ID	Time	Mass Found
1	0.54	

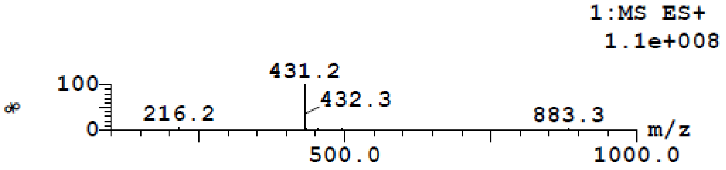

Compound **25**

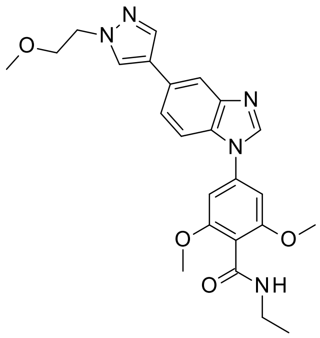

Exact Mass: 449.21
Molecular Formula: $C_{24}H_{27}N_5O_4$

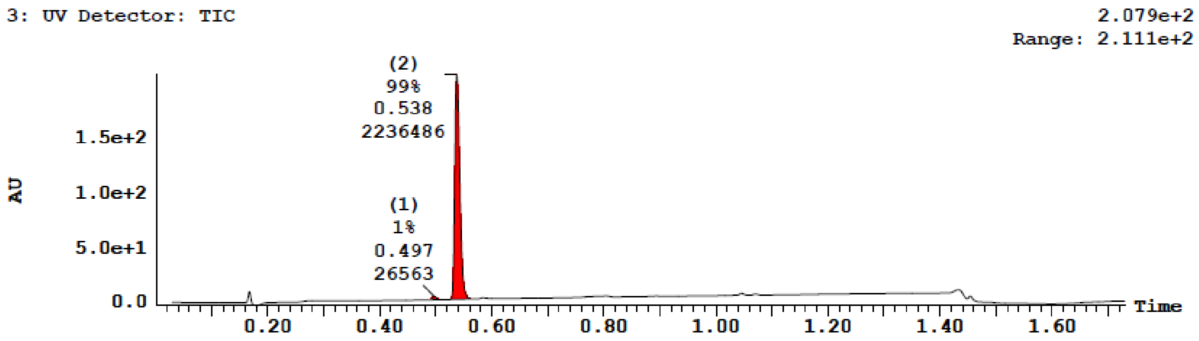

Peak ID	Time	Mass Found
2	0.54	

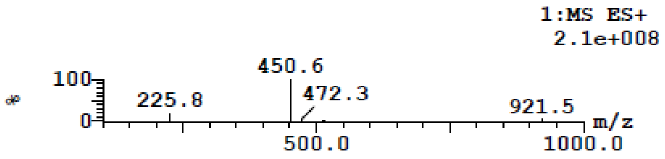

Compound **26**

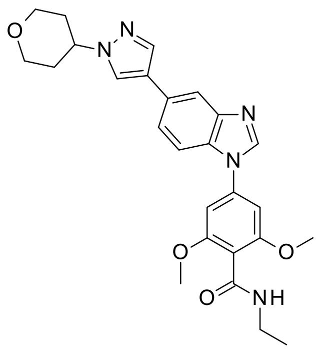

Exact Mass: 475.22
Molecular Formula: $C_{26}H_{29}N_5O_4$

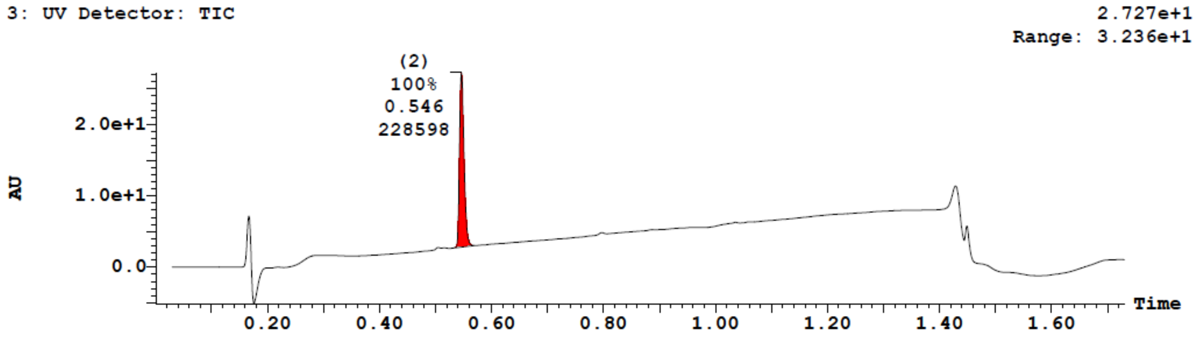

Peak ID	Time	Mass Found
2	0.55	

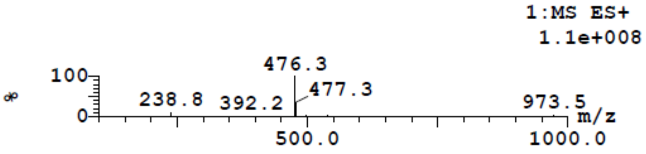

Compound **27**

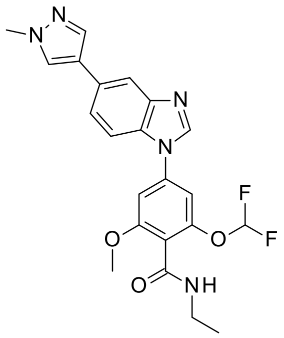

Exact Mass: 441.16
Molecular Formula: $C_{22}H_{21}F_2N_5O_3$

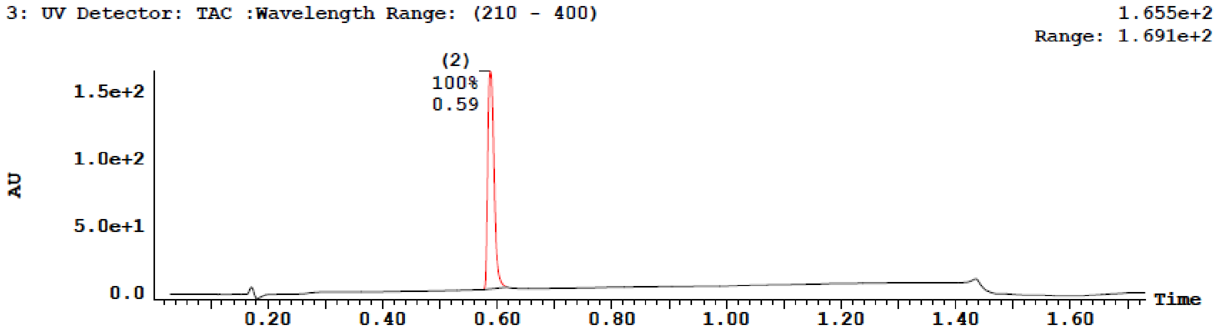

Peak ID	Time	Mass Found
2	0.59	Not Found

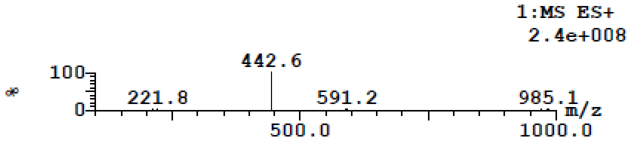

Compound **28**

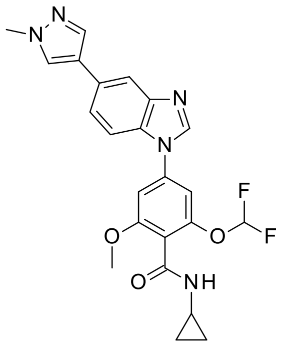

Exact Mass: 453.16
Molecular Formula: $C_{23}H_{21}F_2N_5O_3$

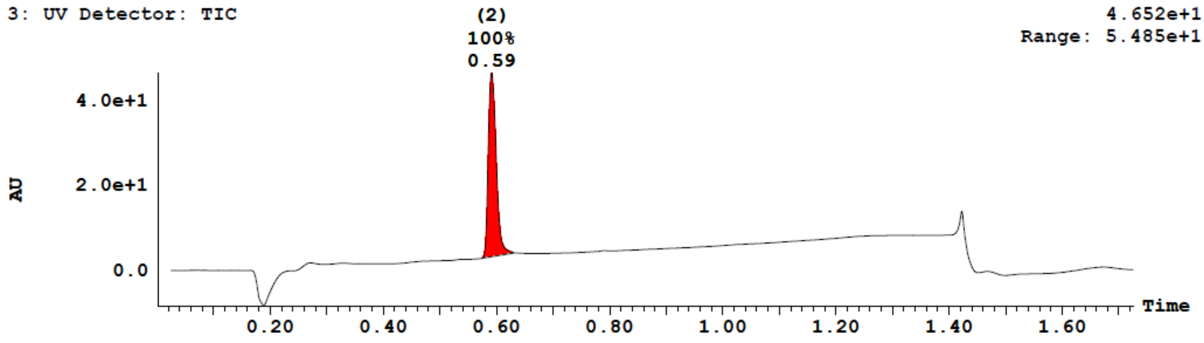

Peak ID	Time	Mass Found
2	0.59	

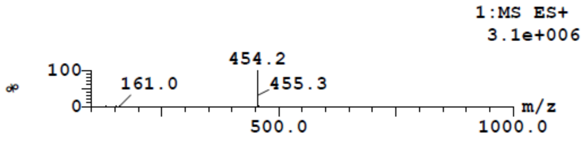

Compound **30**

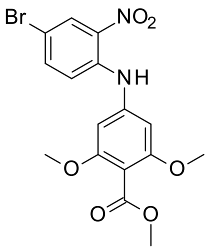

Exact Mass: 410.01

Molecular Formula: $C_{16}H_{15}BrN_2O_6$

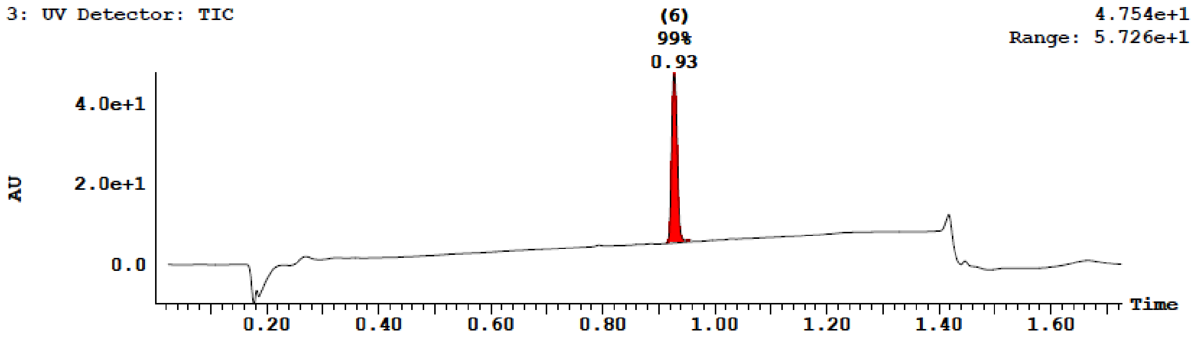

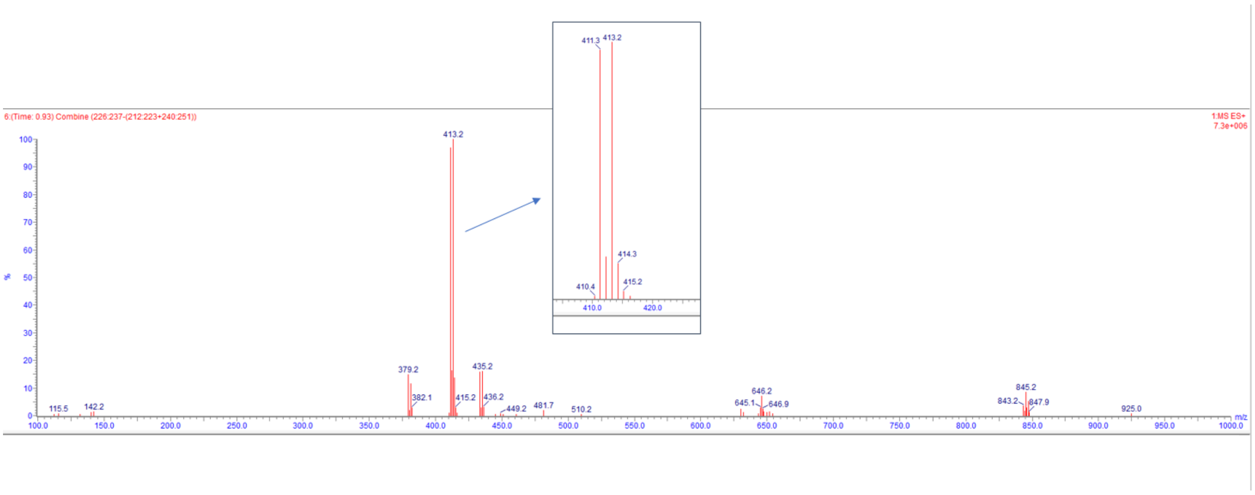

Compound **31**

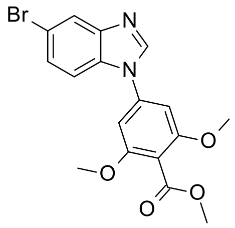

Exact Mass: 390.02
Molecular Formula: $C_{17}H_{15}BrN_2O_4$

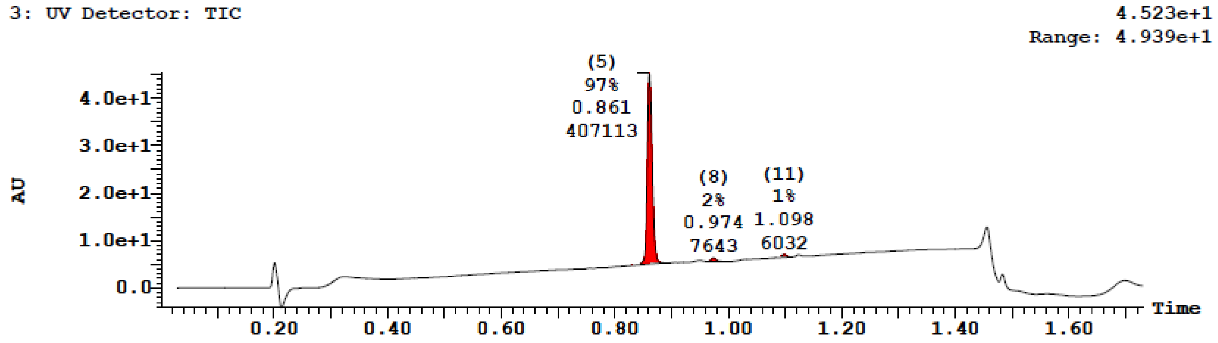

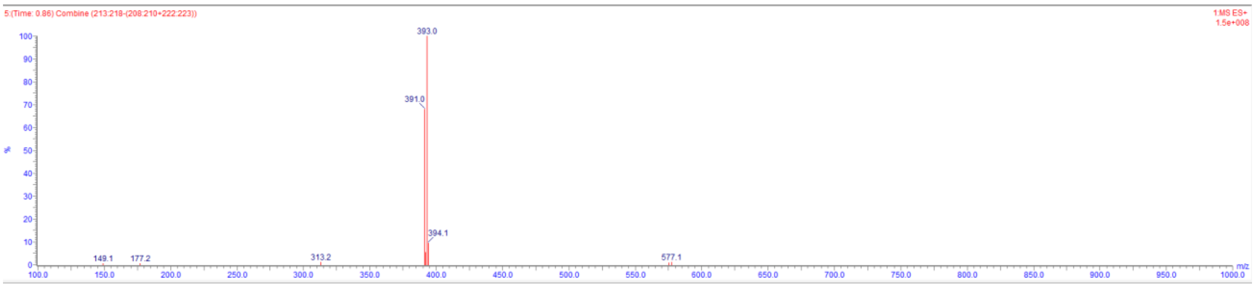

Compound **32**

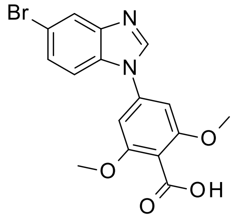

Exact Mass: 376.01
Molecular Formula: $C_{16}H_{13}BrN_2O_4$

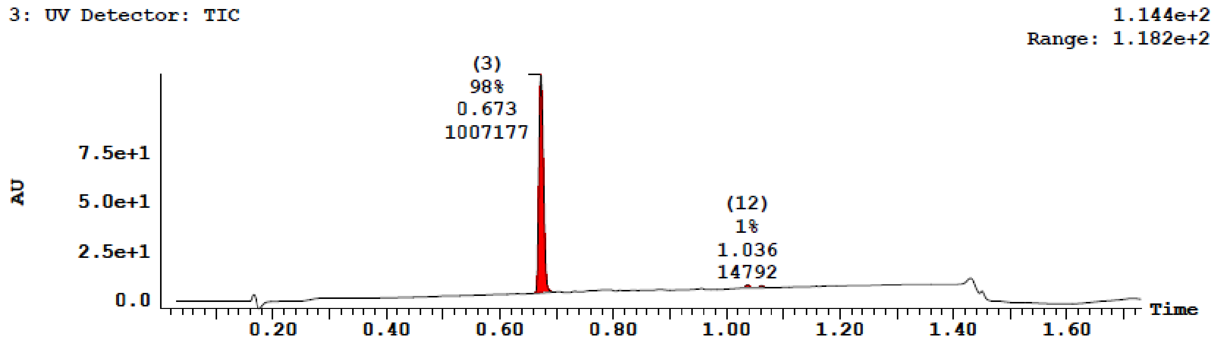

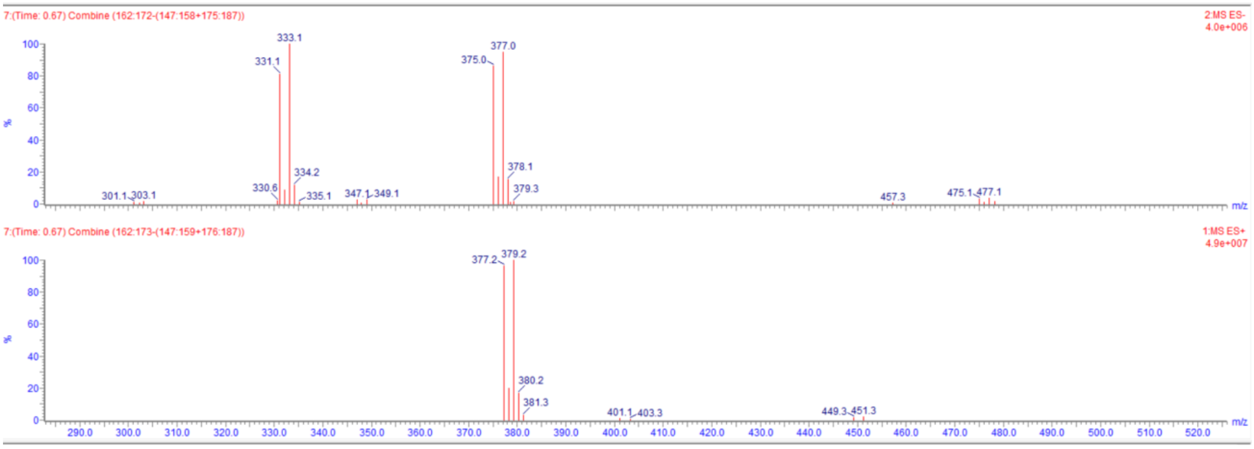

Compound **33a**

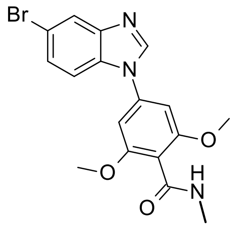

Exact Mass: 389.04
Molecular Formula: $C_{17}H_{16}BrN_3O_3$

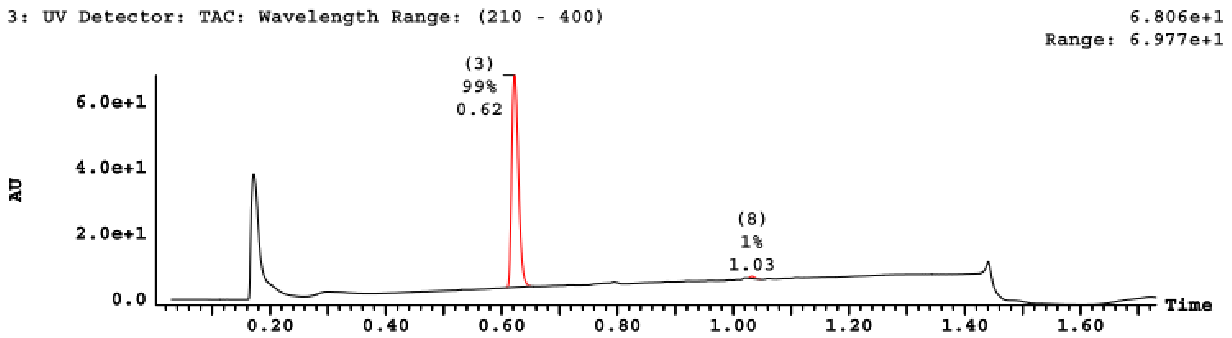

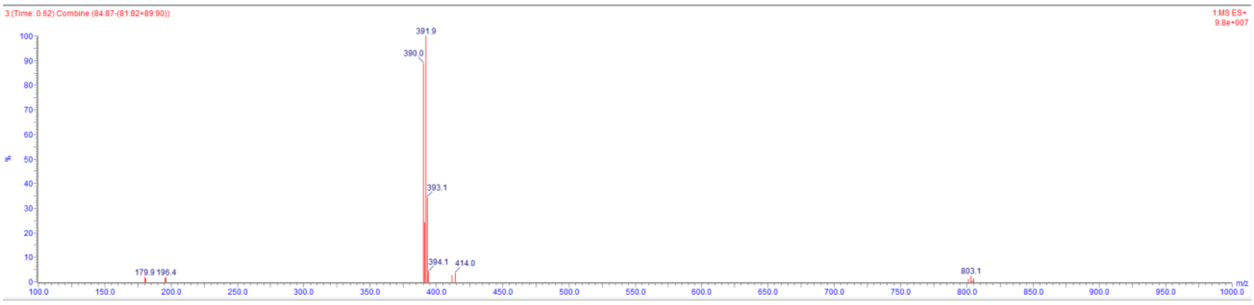

Compound **33b**

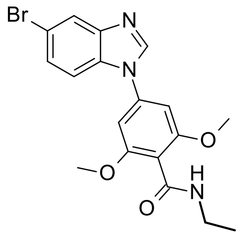

Exact Mass: 403.05
Molecular Formula: $C_{18}H_{18}BrN_3O_3$

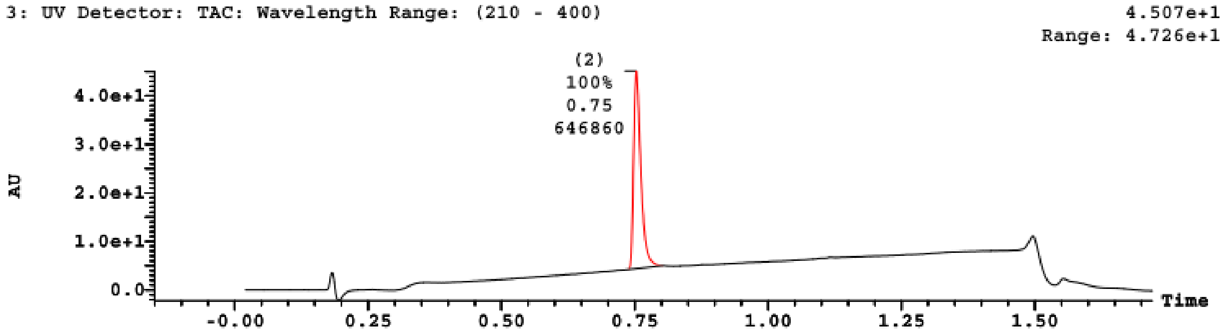

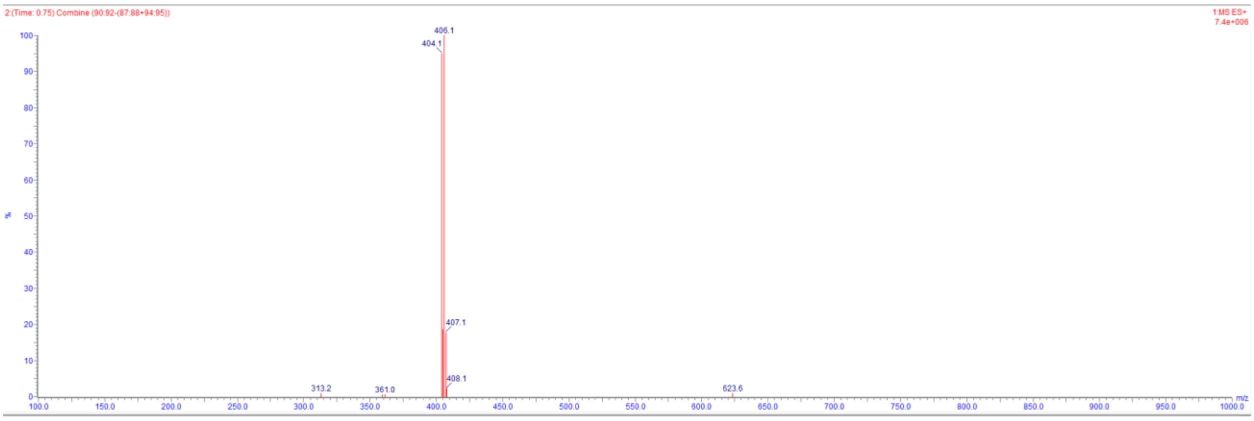

Compound **33c**

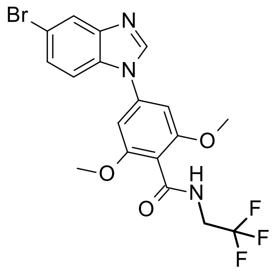

Exact Mass: 457.02
Molecular Formula: $C_{18}H_{15}BrF_3N_3O_3$

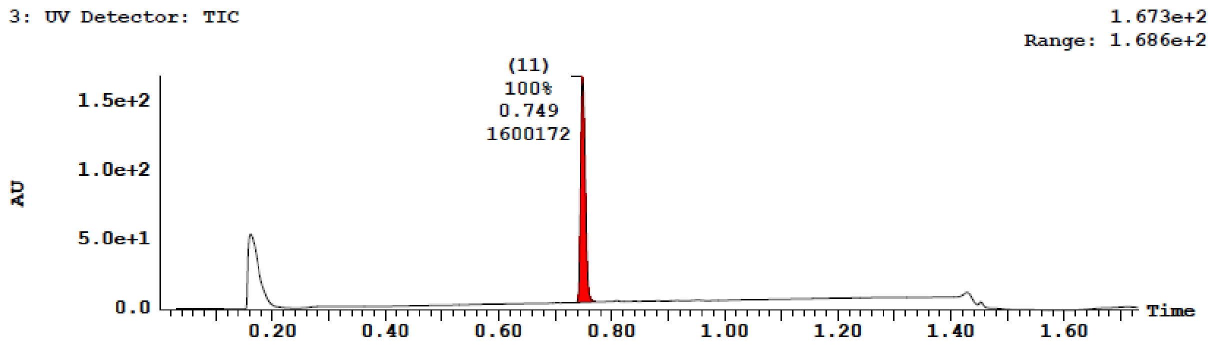

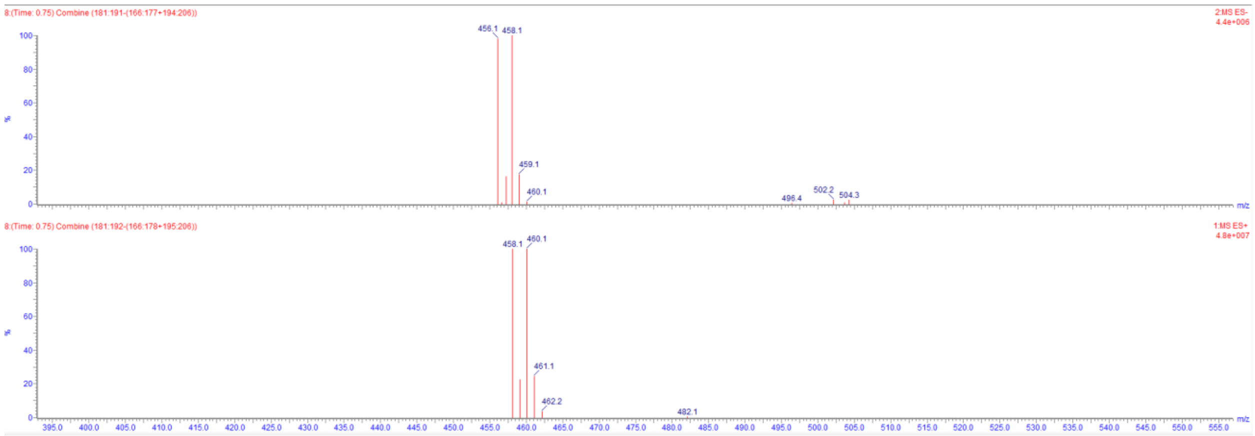

Compound **33d**

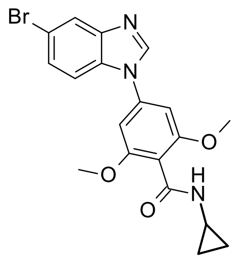

Exact Mass: 415.05
Molecular Formula: $C_{19}H_{18}BrN_3O_3$

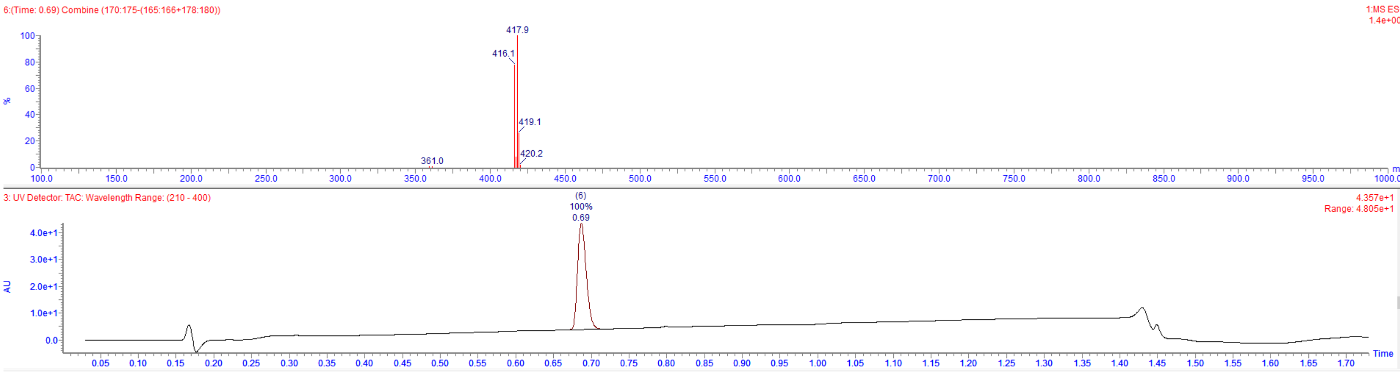

Compound **33e**

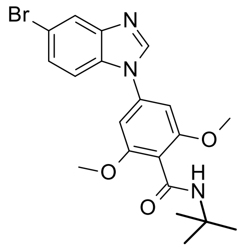

Exact Mass: 431.08
Molecular Formula: $C_{20}H_{22}BrN_3O_3$

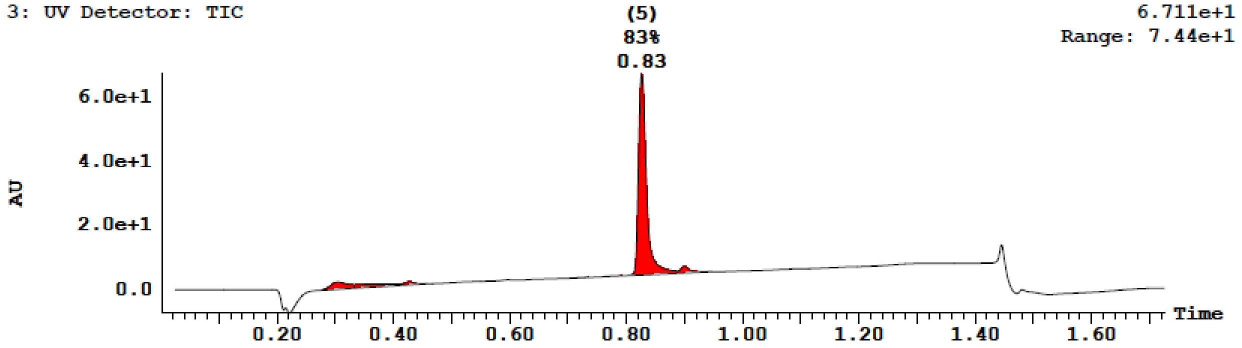

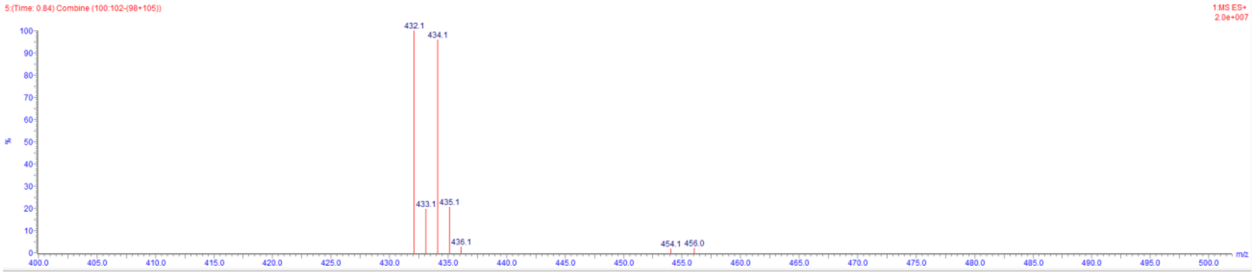

Compound **33f**

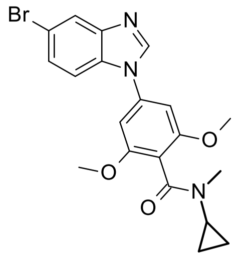

Exact Mass: 429.07
Molecular Formula: $C_{20}H_{20}BrN_3O_3$

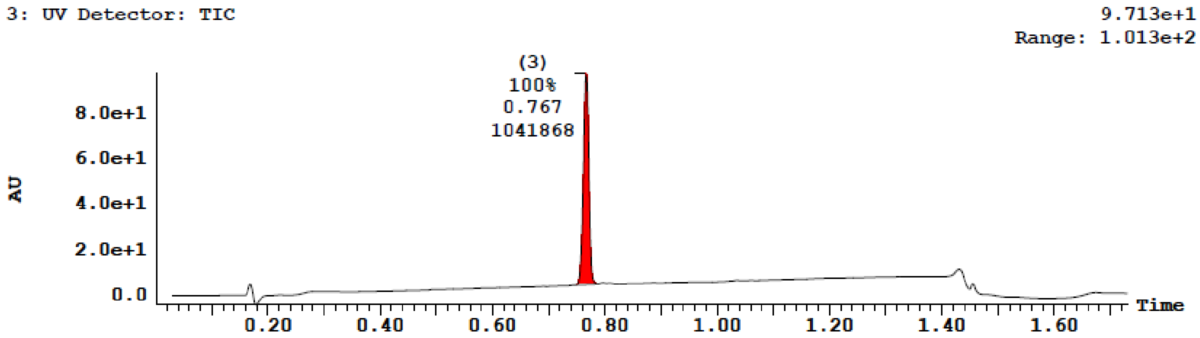

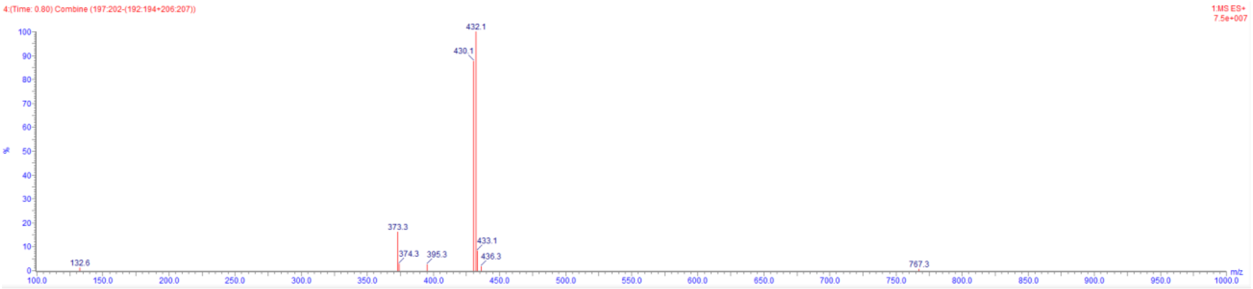

Compound **34**

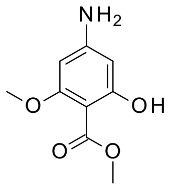

Formula Weight: 197.18794
Molecular Formula: $C_9H_{11}NO_4$

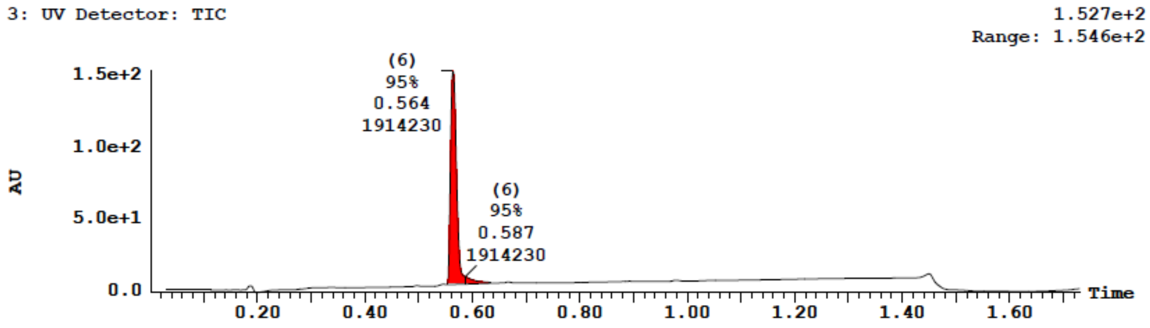

Peak ID	Time	Mass Found
6	0.57	

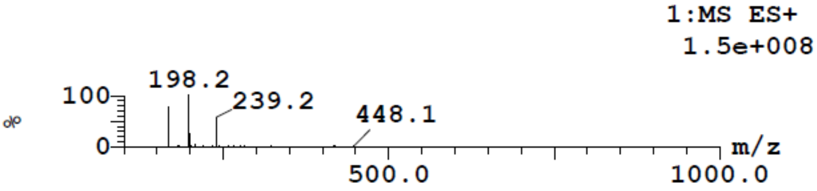

Compound **35**

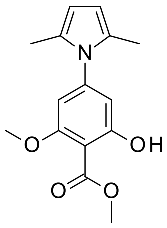

Exact Mass: 275.12
Molecular Formula: $C_{15}H_{17}NO_4$

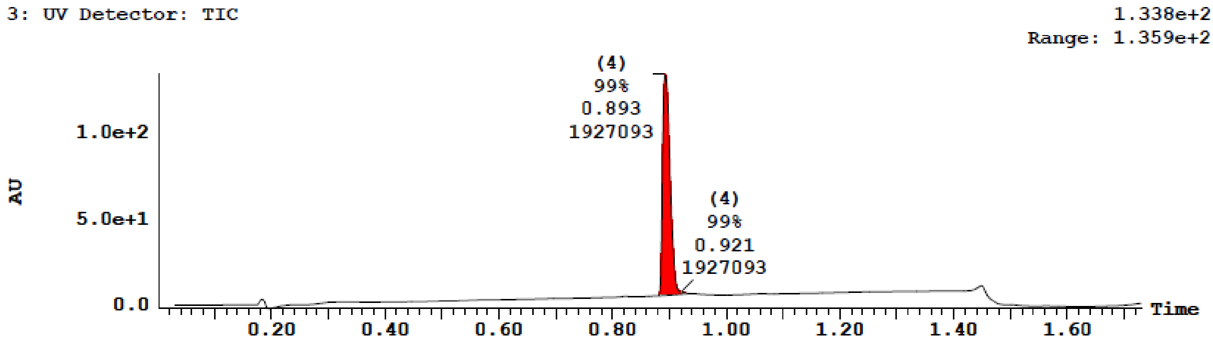

Peak ID	Time	Mass Found
4	0.89	

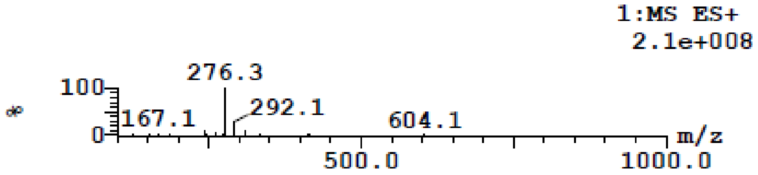

Compound **36**

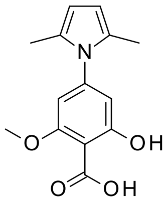

Exact Mass: 261.1
Molecular Formula: $C_{14}H_{15}NO_4$

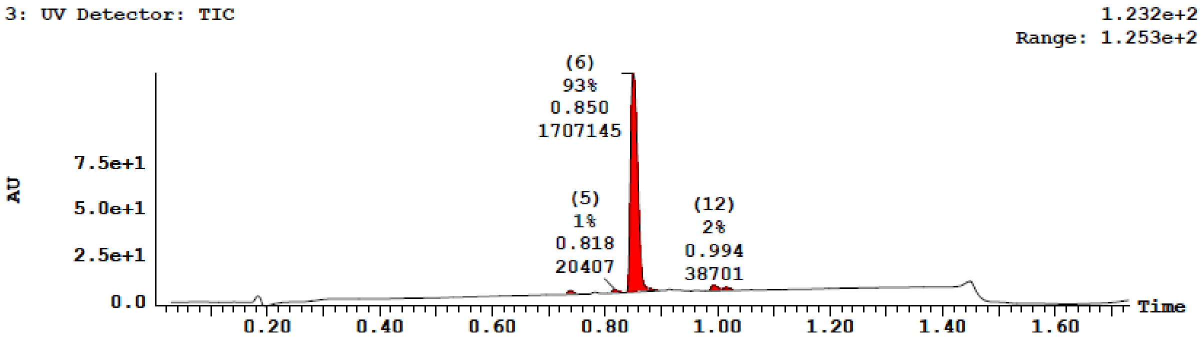

Peak ID	Time	Mass Found
6	0.85	

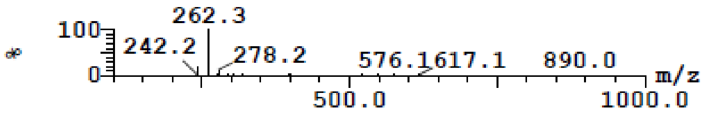

Compound **37a**

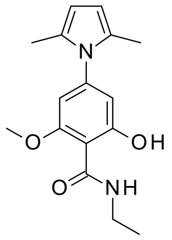

Exact Mass: 288.15
Molecular Formula: $C_{16}H_{20}N_2O_3$

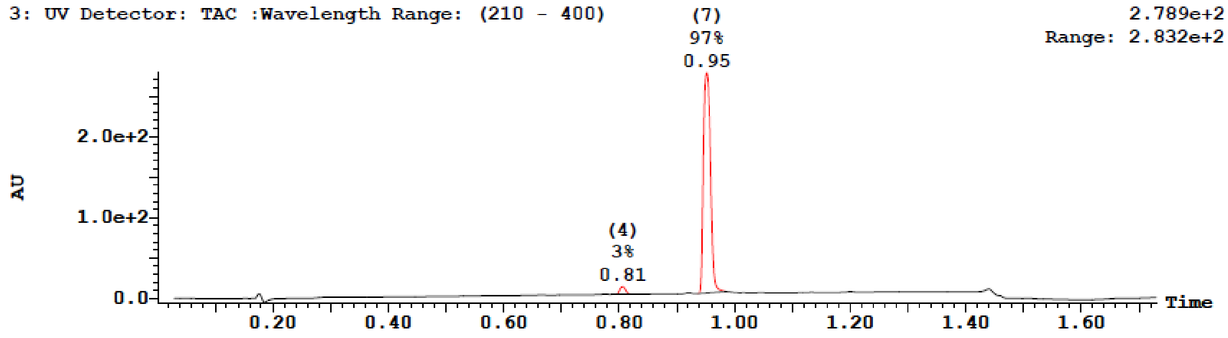

Peak ID	Time	Mass Found
7	0.95	Not Found

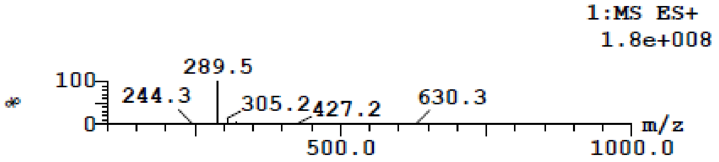

Compound **37b**

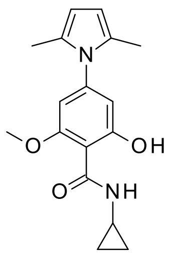

Exact Mass: 300.15
Molecular Formula: $C_{17}H_{20}N_2O_3$

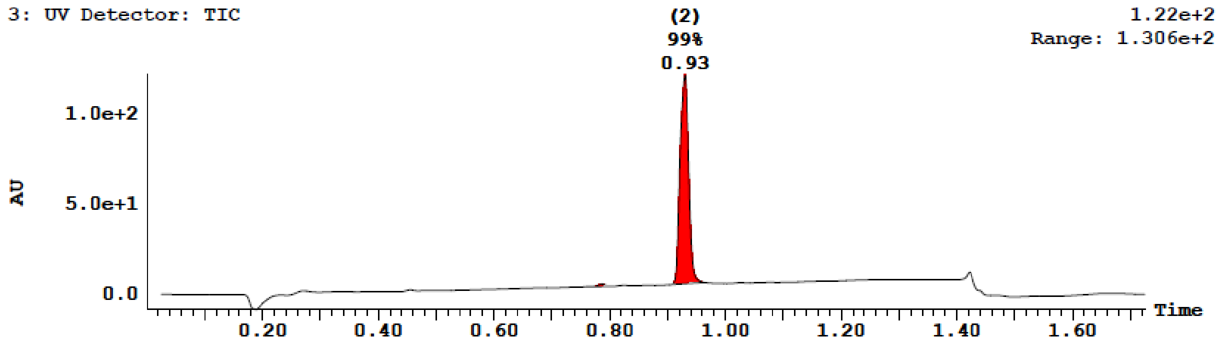

Peak ID	Time	Mass Found
2	0.94	

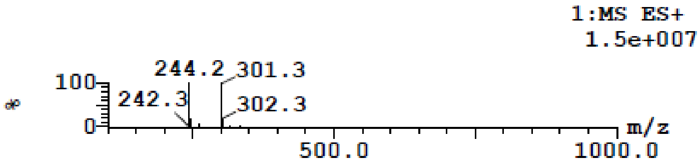

Compound **38a**

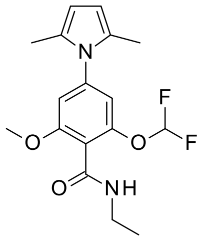

Exact Mass: 338.14
Molecular Formula: $C_{17}H_{20}F_2N_2O_3$

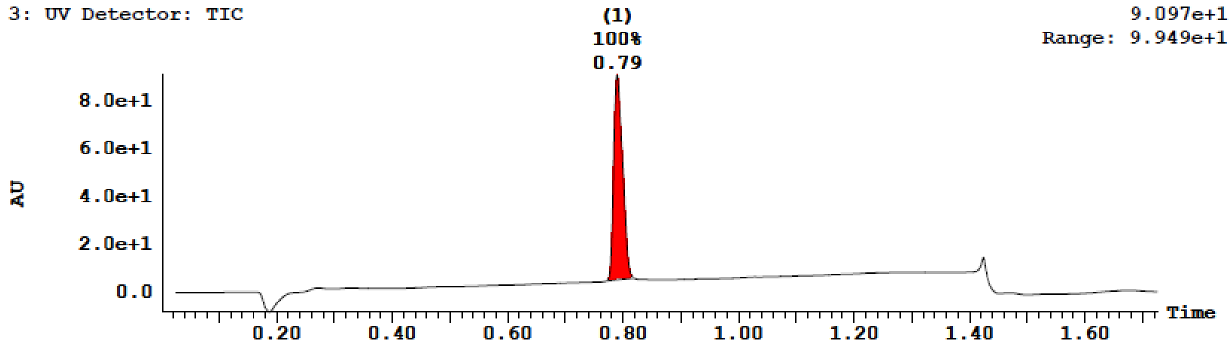

Peak ID	Time	Mass Found
1	0.79	

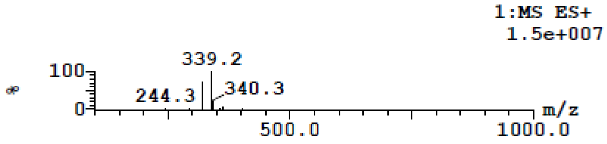

Compound **38b**

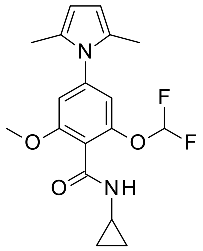

Exact Mass: 350.14
Molecular Formula: $C_{18}H_{20}F_2N_2O_3$

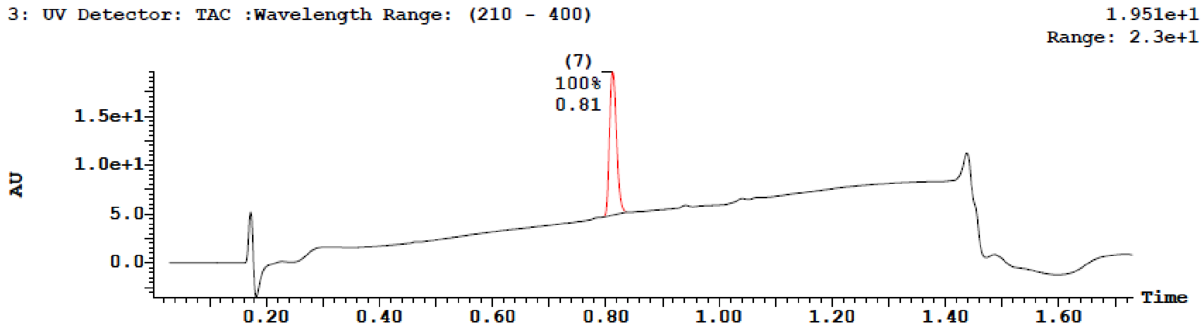

Peak ID	Time	Mass Found
7	0.81	Not Found

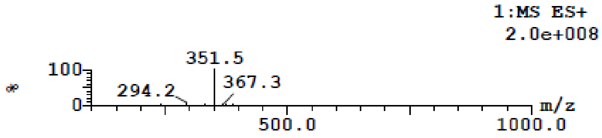

Compound **39a**

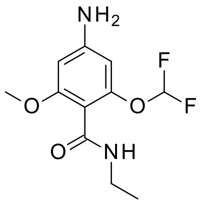

Exact Mass: 260.1
Molecular Formula: $C_{11}H_{14}F_2N_2O_3$

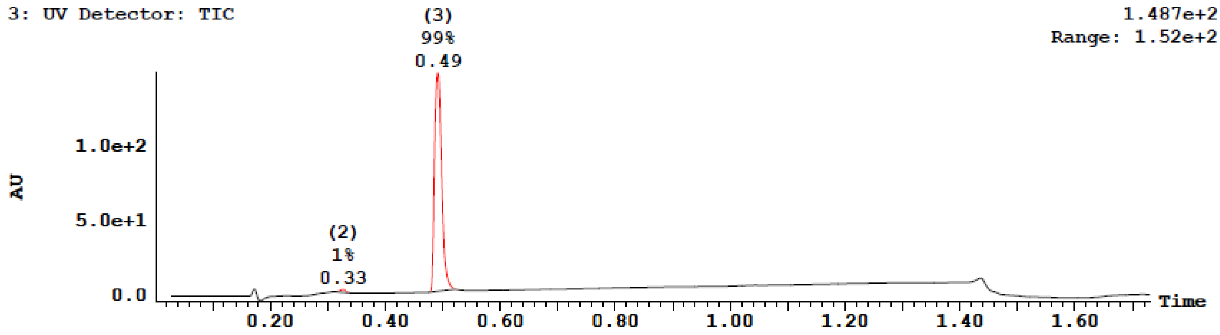

Peak ID	Time	Mass Found
3	0.49	

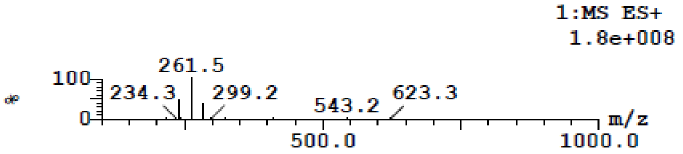

Compound **39b**

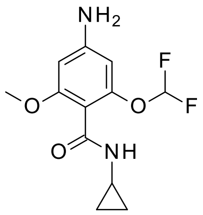

Exact Mass: 272.1
Molecular Formula: $C_{12}H_{14}F_2N_2O_3$

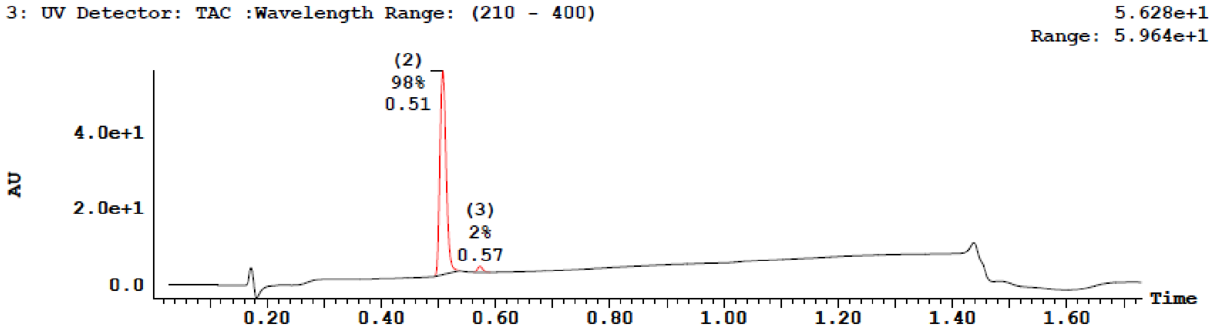

Peak ID	Time	Mass Found
2	0.51	Not Found

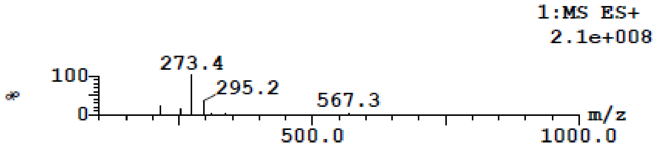

Compound **40a**

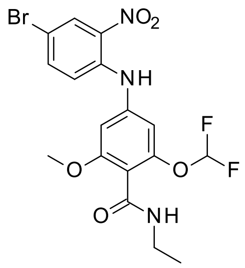

Exact Mass: 459.02
Molecular Formula: $C_{17}H_{16}BrF_2N_3O_5$

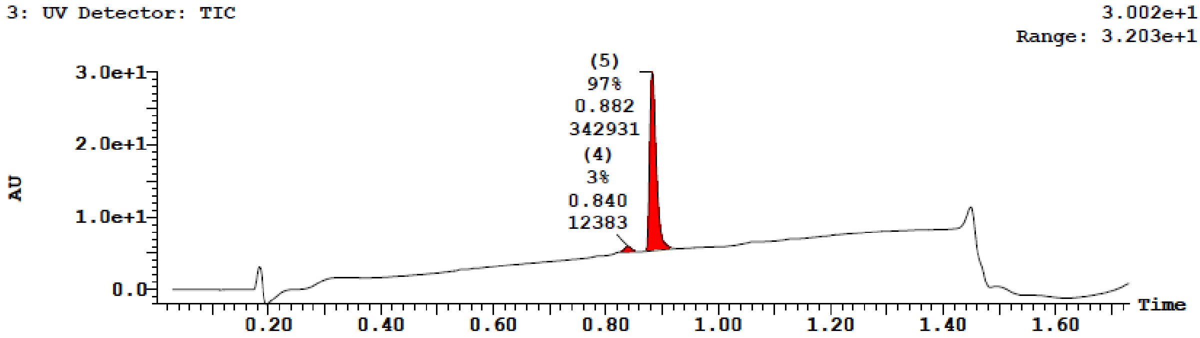

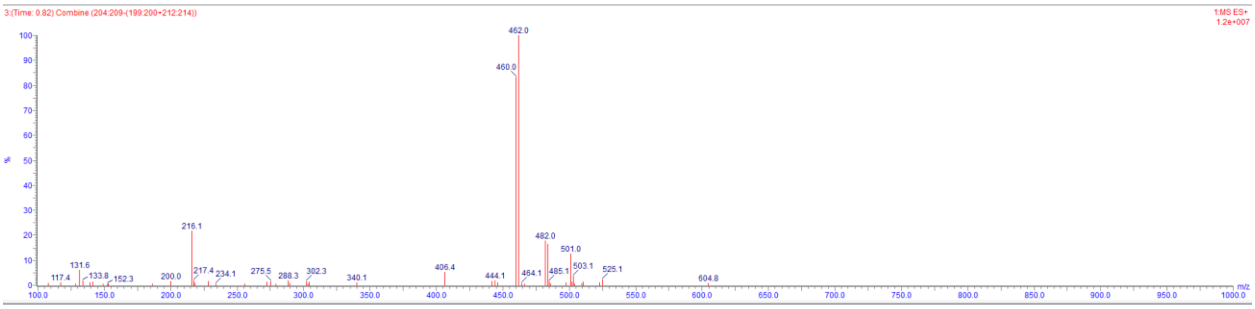

Compound **40b**

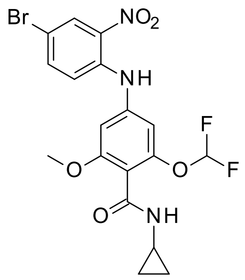

Exact Mass: 471.02
Molecular Formula: $C_{18}H_{16}BrF_2N_3O_5$

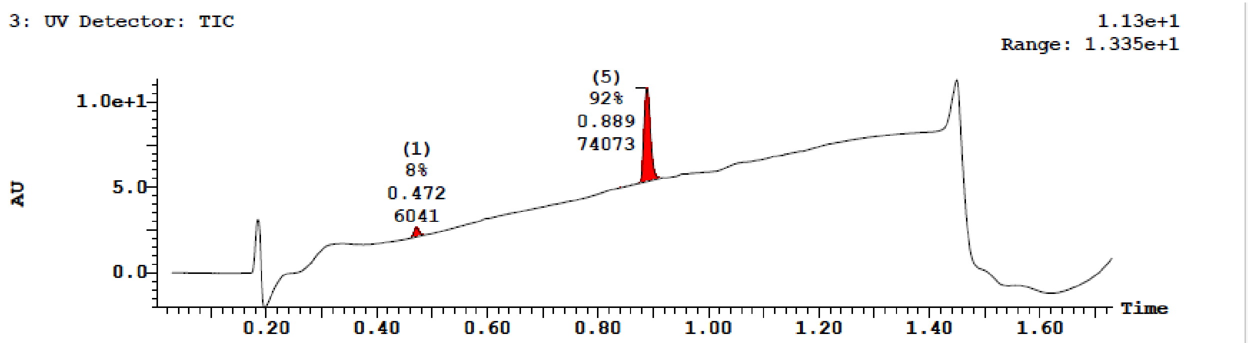

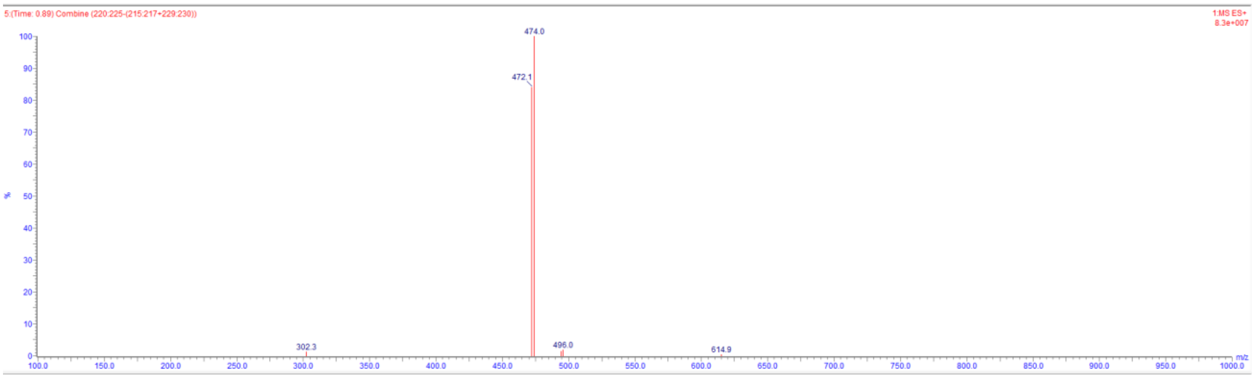

Compound **41a**

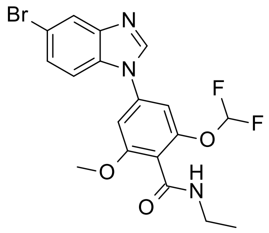

Exact Mass: 439.03

Molecular Formula: $C_{18}H_{16}BrF_2N_3O_3$

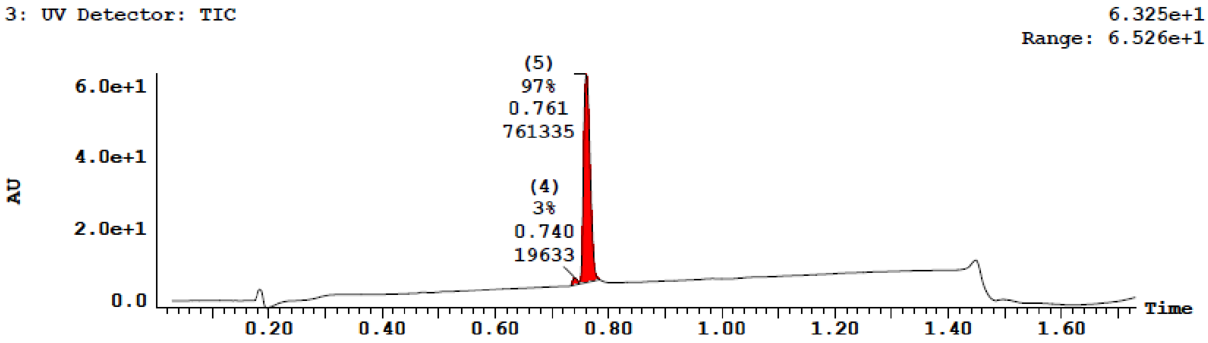

m/z = 484.2- 486.1 [M - H] + 46 (formic acid)

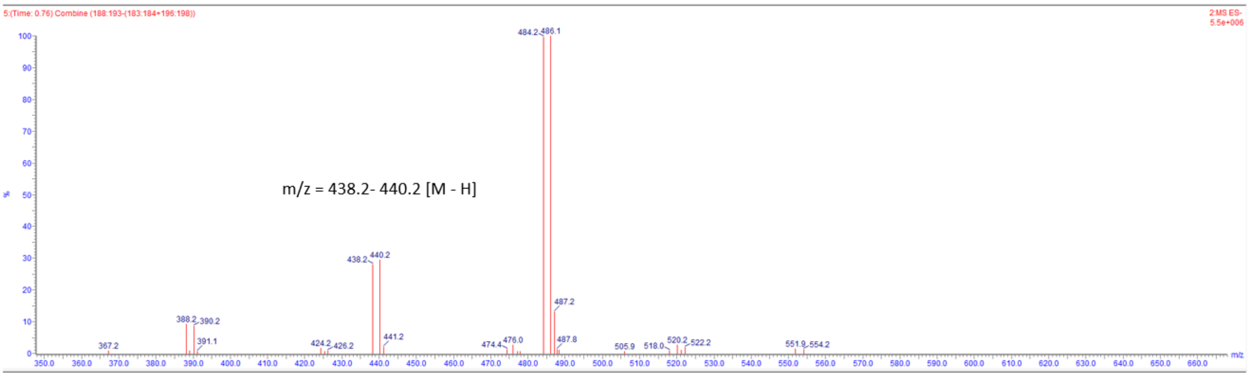

Compound **41b**

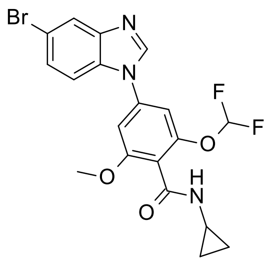

Exact Mass: 451.03
Molecular Formula: $C_{19}H_{16}BrF_2N_3O_3$

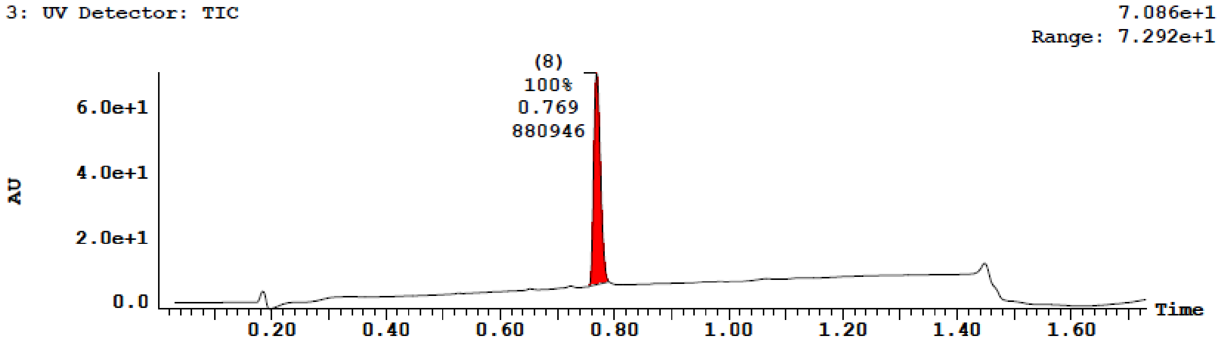

m/z = 450.1- 452.1 [M - H]

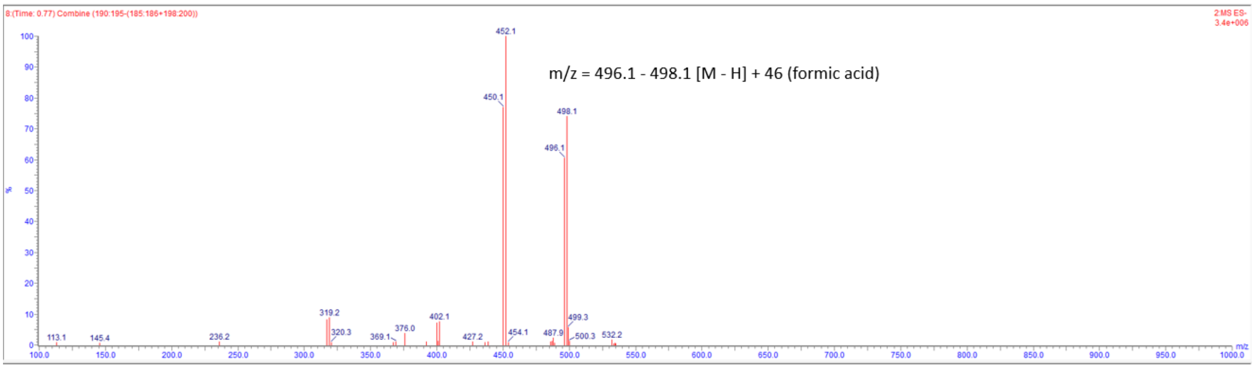

NMR traces

Compound **8**

Molecular Formula: $C_{15}H_{12}BrN_3O_2$

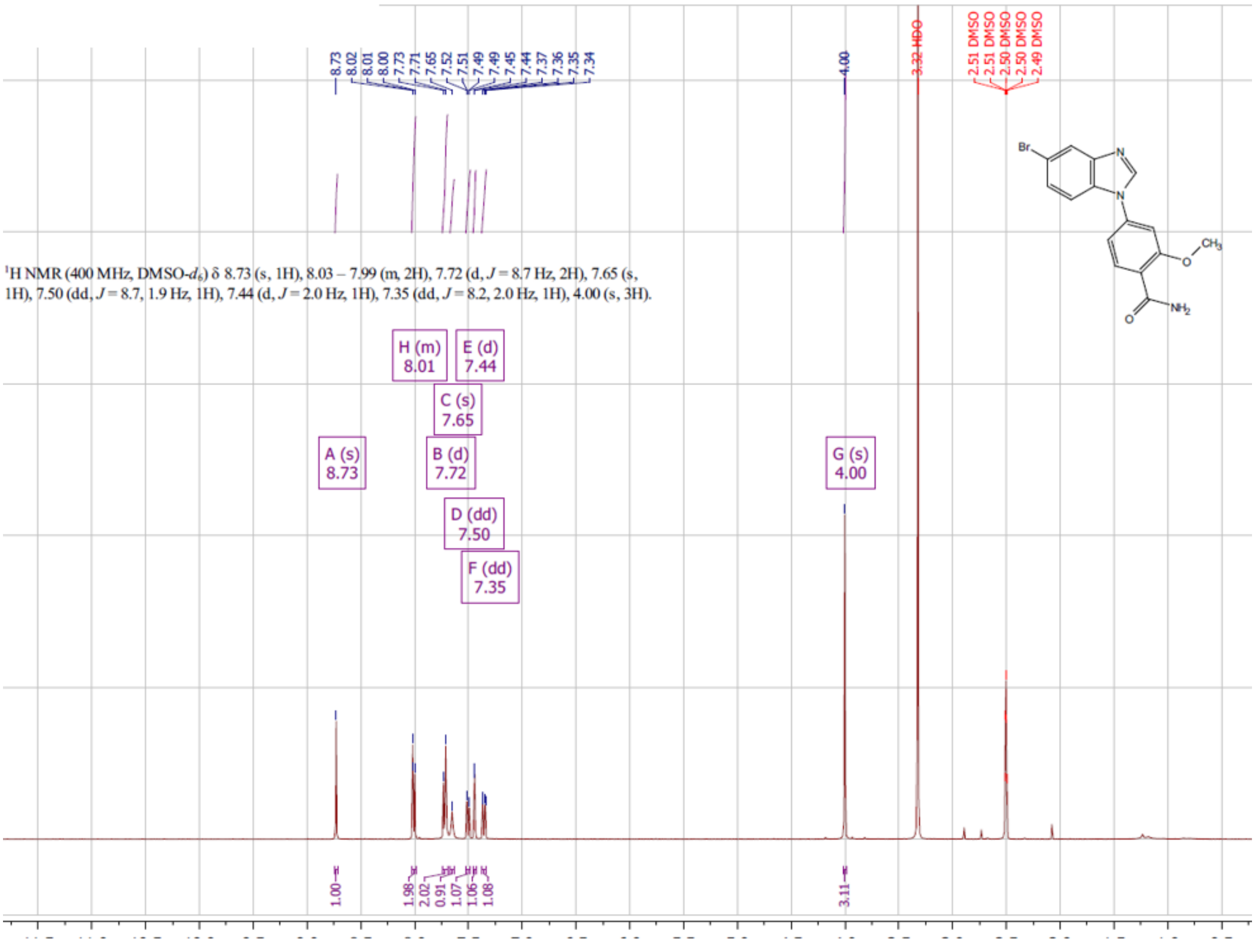

Compound **9**

Molecular Formula: $C_{16}H_{14}BrN_3O_3$

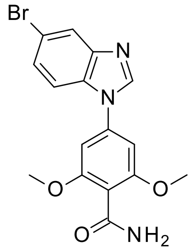

^{1}H NMR (400 MHz, DMSO-d_6)

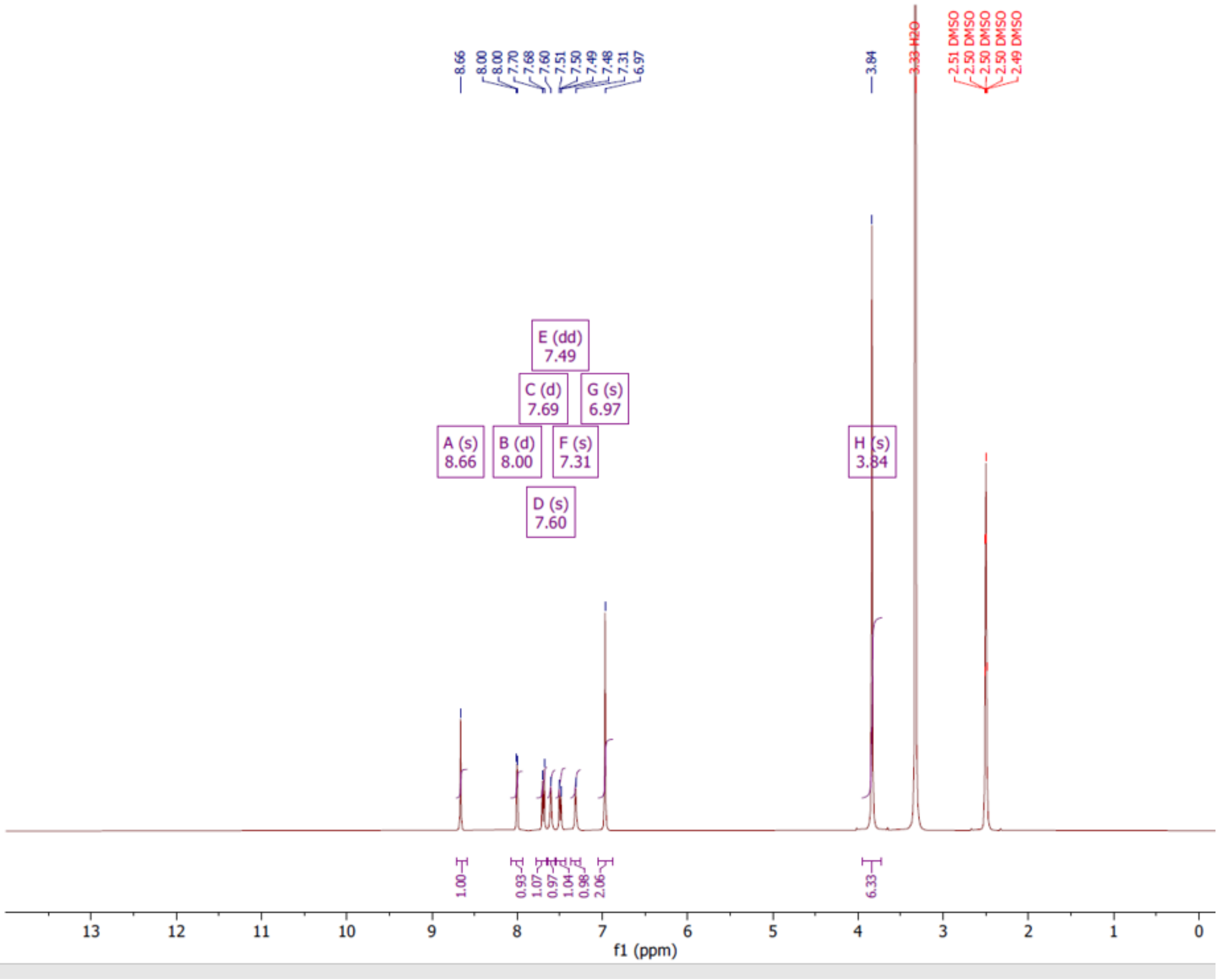

Compound **10**

Molecular Formula: $C_{21}H_{21}N_5O_3$

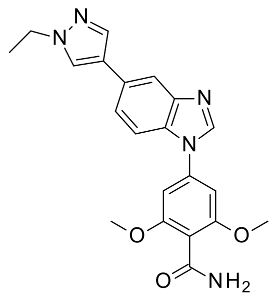

^{1}H NMR (400 MHz, DMSO-d_6)

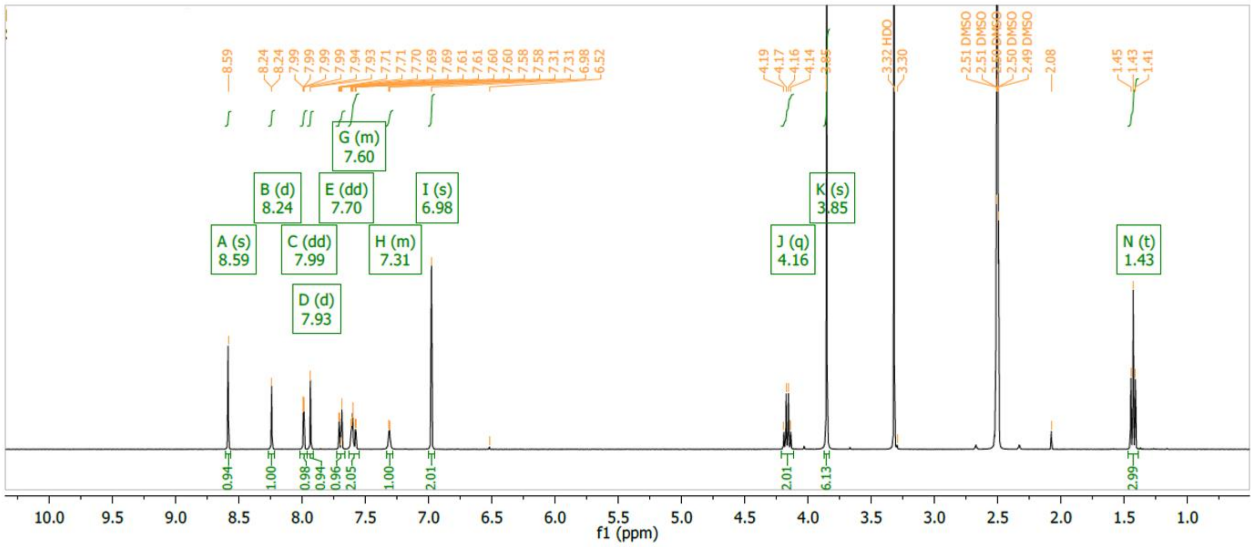

Compound **11**

Molecular Formula: $C_{22}H_{22}N_4O_4$

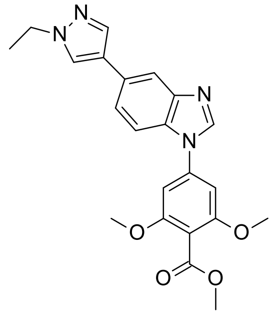

^{1}H NMR (400 MHz, Chloroform-d)

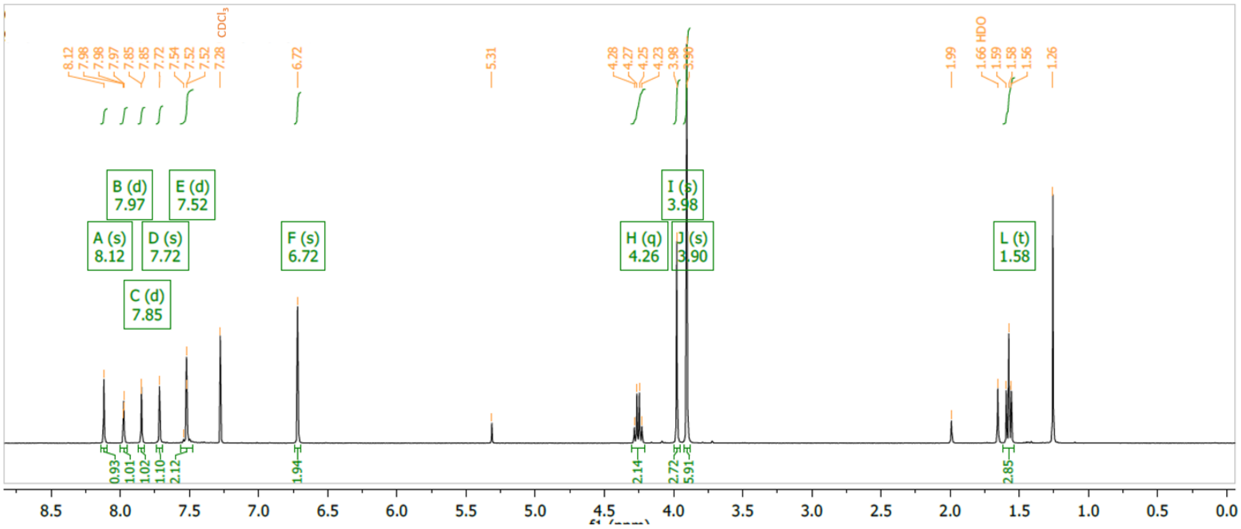

Compound **12**

Molecular Formula: $C_{21}H_{22}N_4O_3$

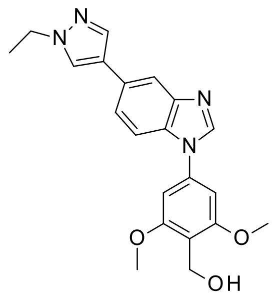

^{1}H NMR (400 MHz, Chloroform-*d*)

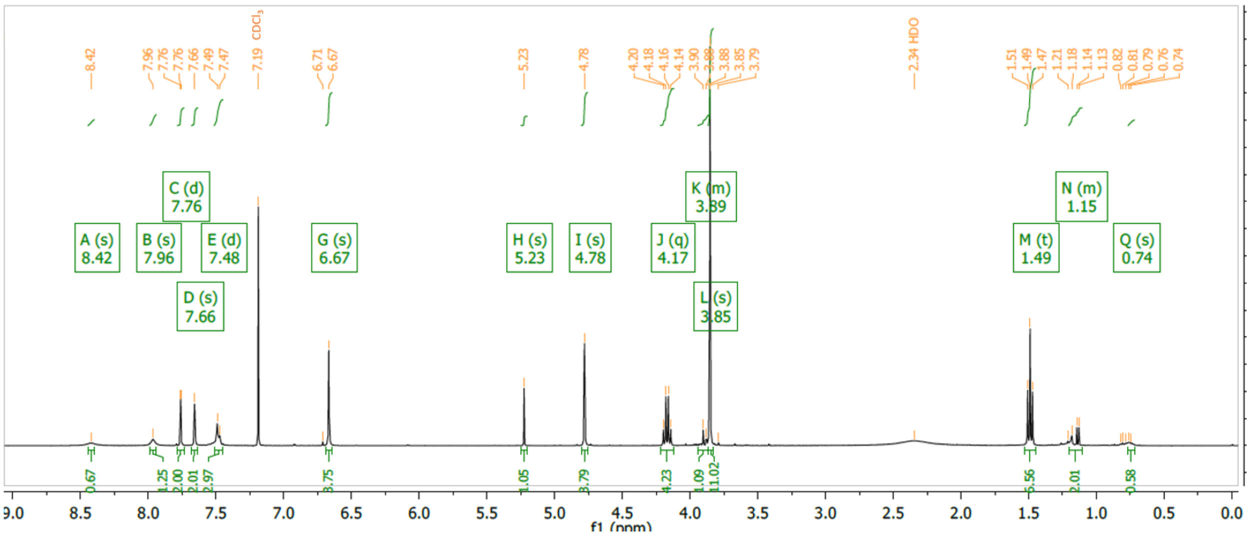

Compound **13**

Molecular Formula: $C_{21}H_{20}N_4O_4$

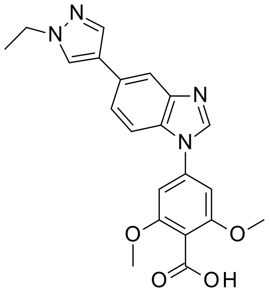

^{1}H NMR (400 MHz, DMSO-d_6)

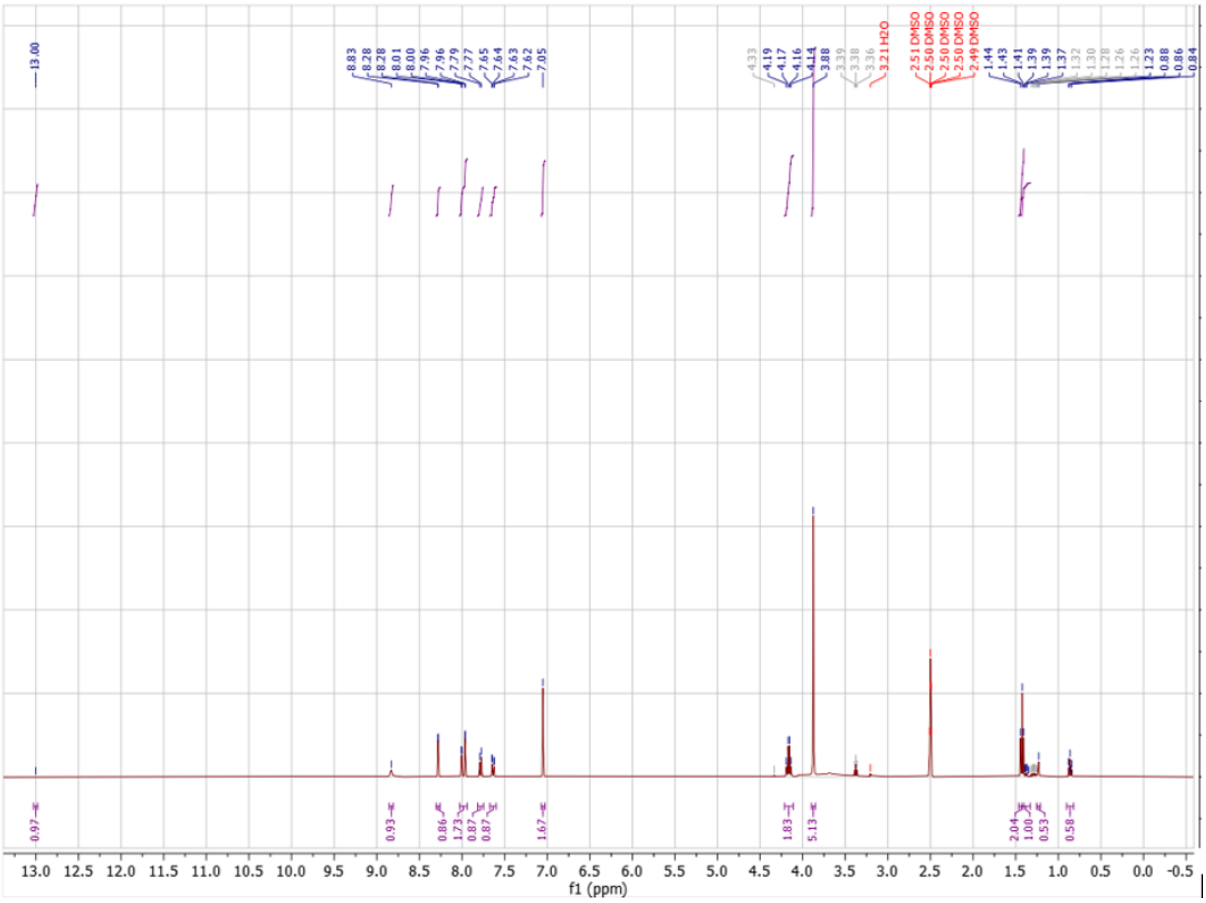

Compound **14**

Molecular Formula: $C_{22}H_{23}N_5O_3$

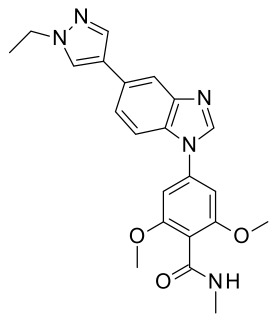

^{1}H NMR (400 MHz, DMSO-d_6)

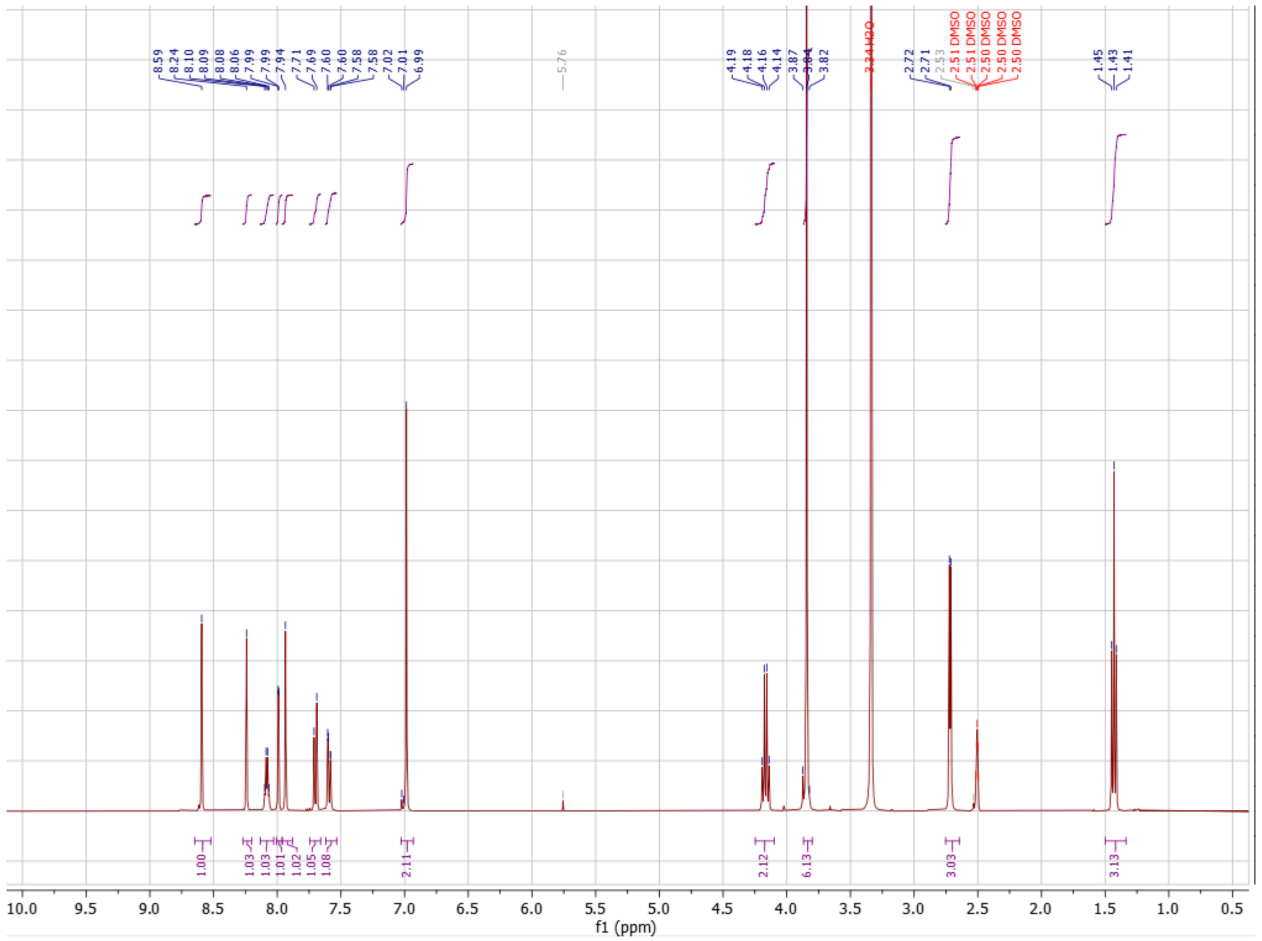

Compound **15**

Molecular Formula: $C_{23}H_{25}N_5O_3$

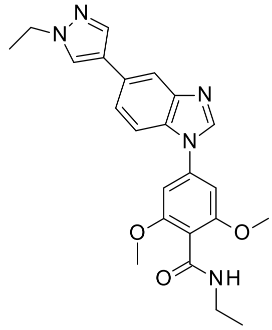

^{1}H NMR (400 MHz, DMSO-d_6)

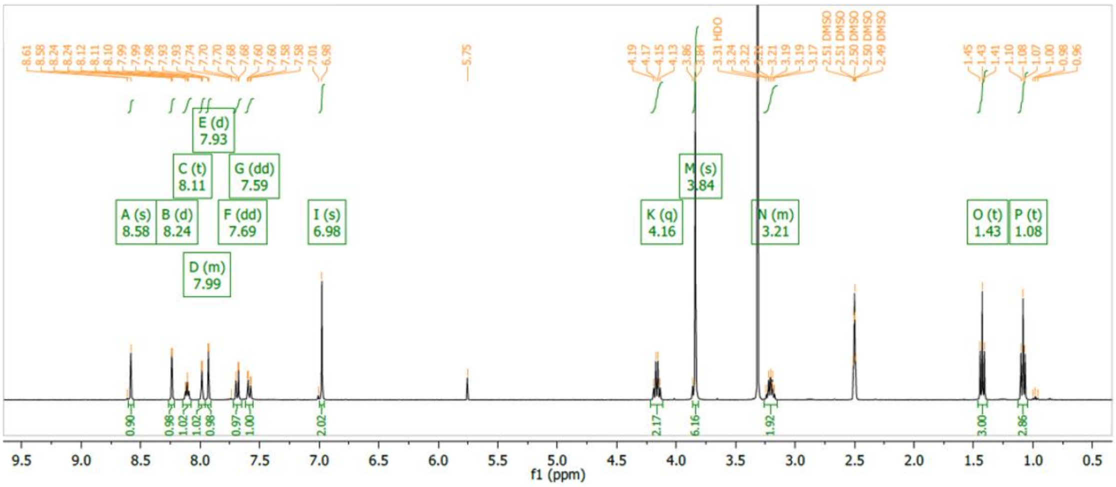

Compound **16**

Molecular Formula: $C_{23}H_{22}F_3N_5O_3$

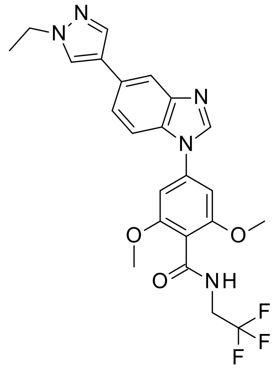

^1H NMR (400 MHz, DMSO-d_6)

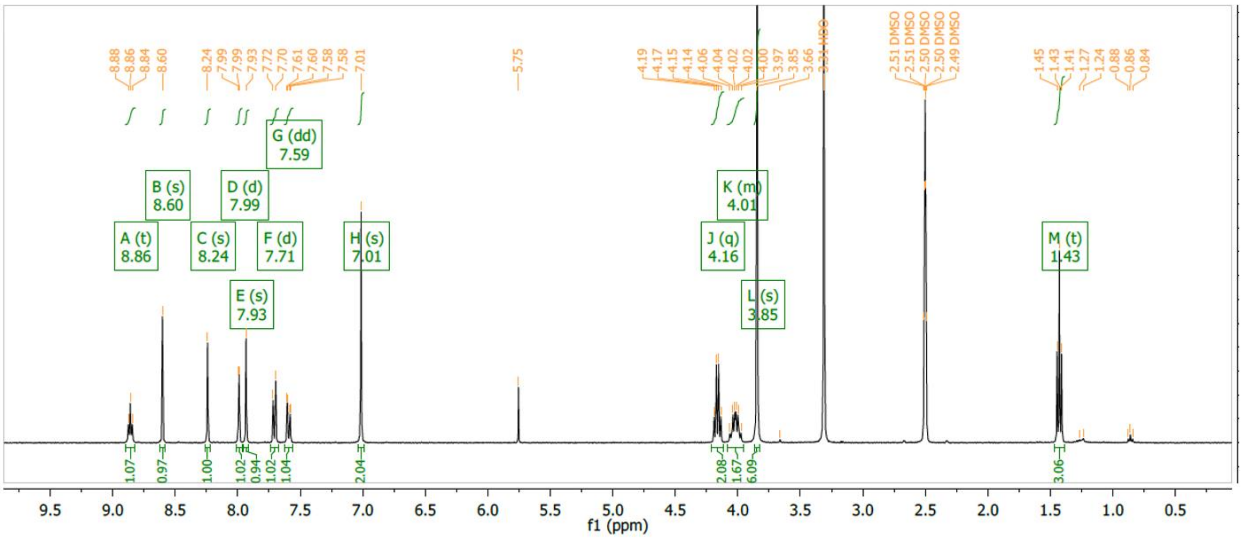

Compound **17**

Molecular Formula: $C_{24}H_{25}N_5O_3$

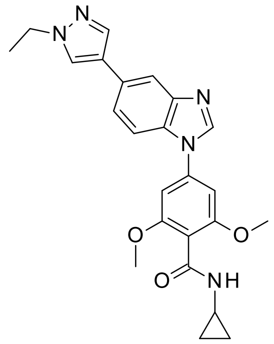

^{1}H NMR (400 MHz, Chloroform-*d*)

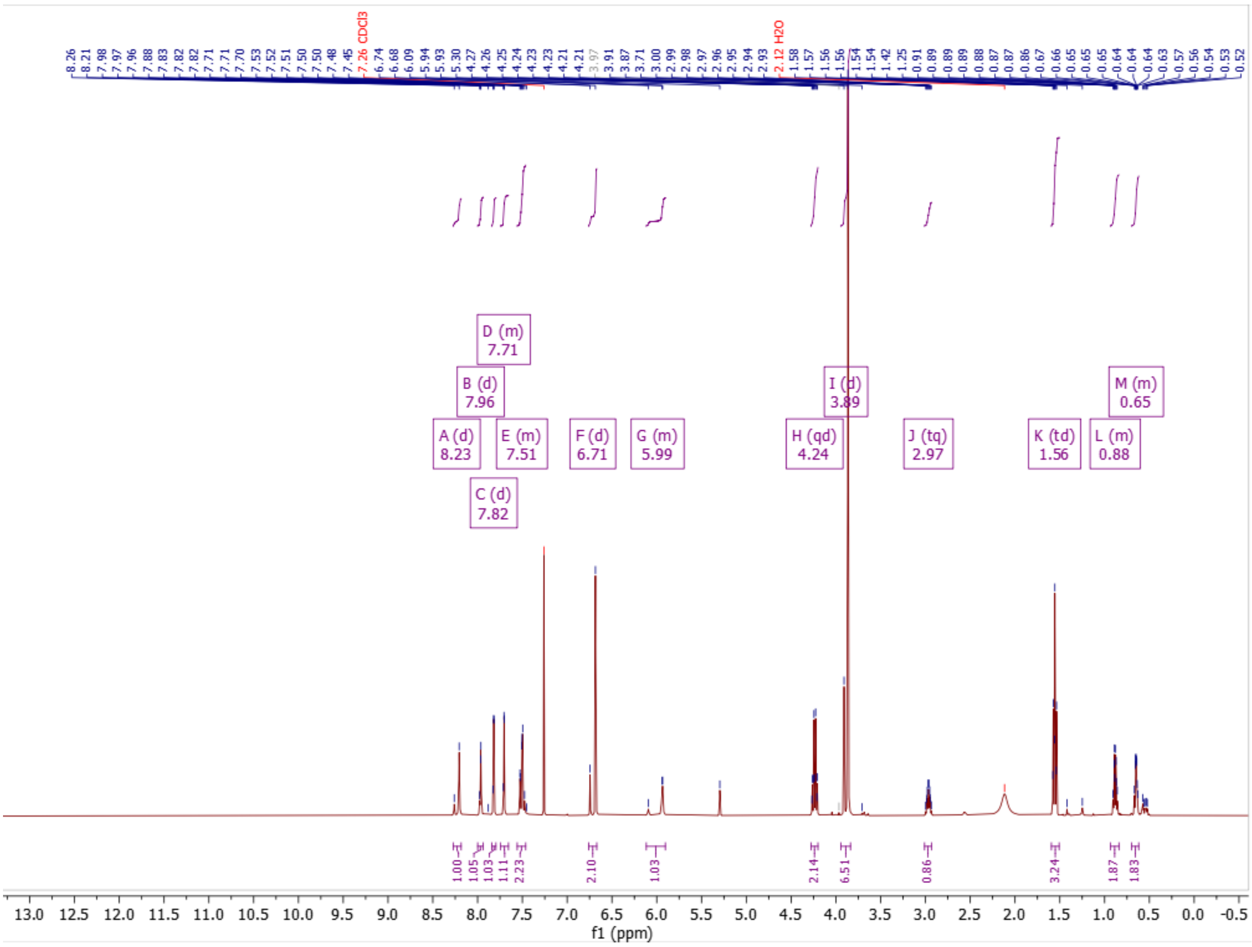

Compound **18**

Molecular Formula: $C_{25}H_{29}N_5O_3$

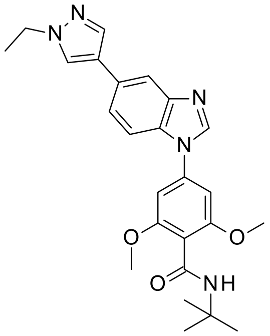

^{1}H NMR (400 MHz, Chloroform-*d*)

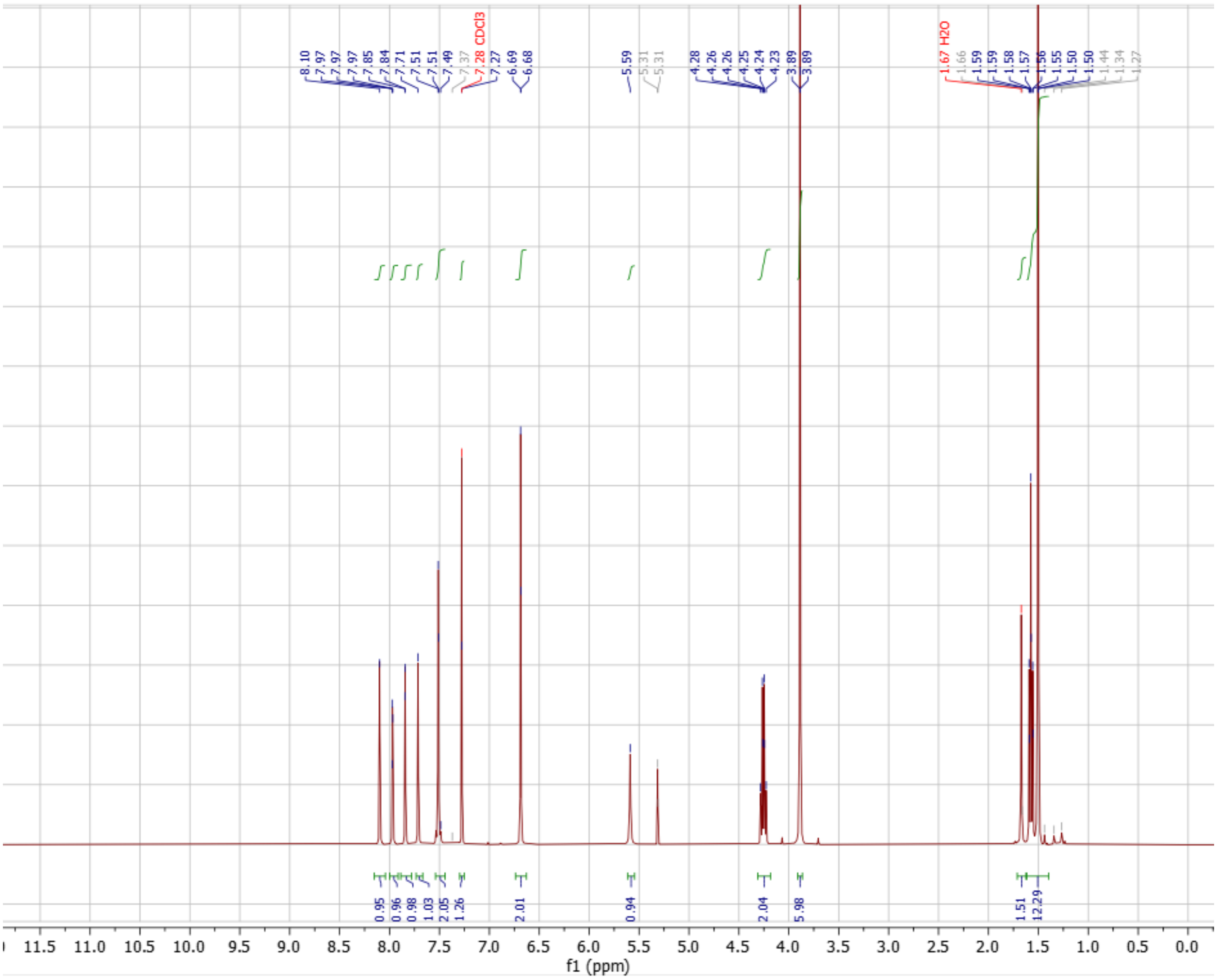

Compound **19**

Molecular Formula: $C_{25}H_{27}N_5O_3$

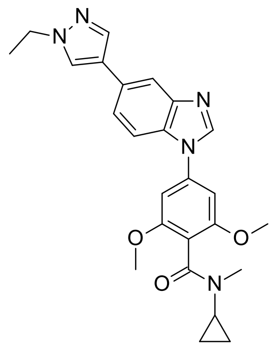

^1H NMR (400 MHz, Chloroform-*d*)

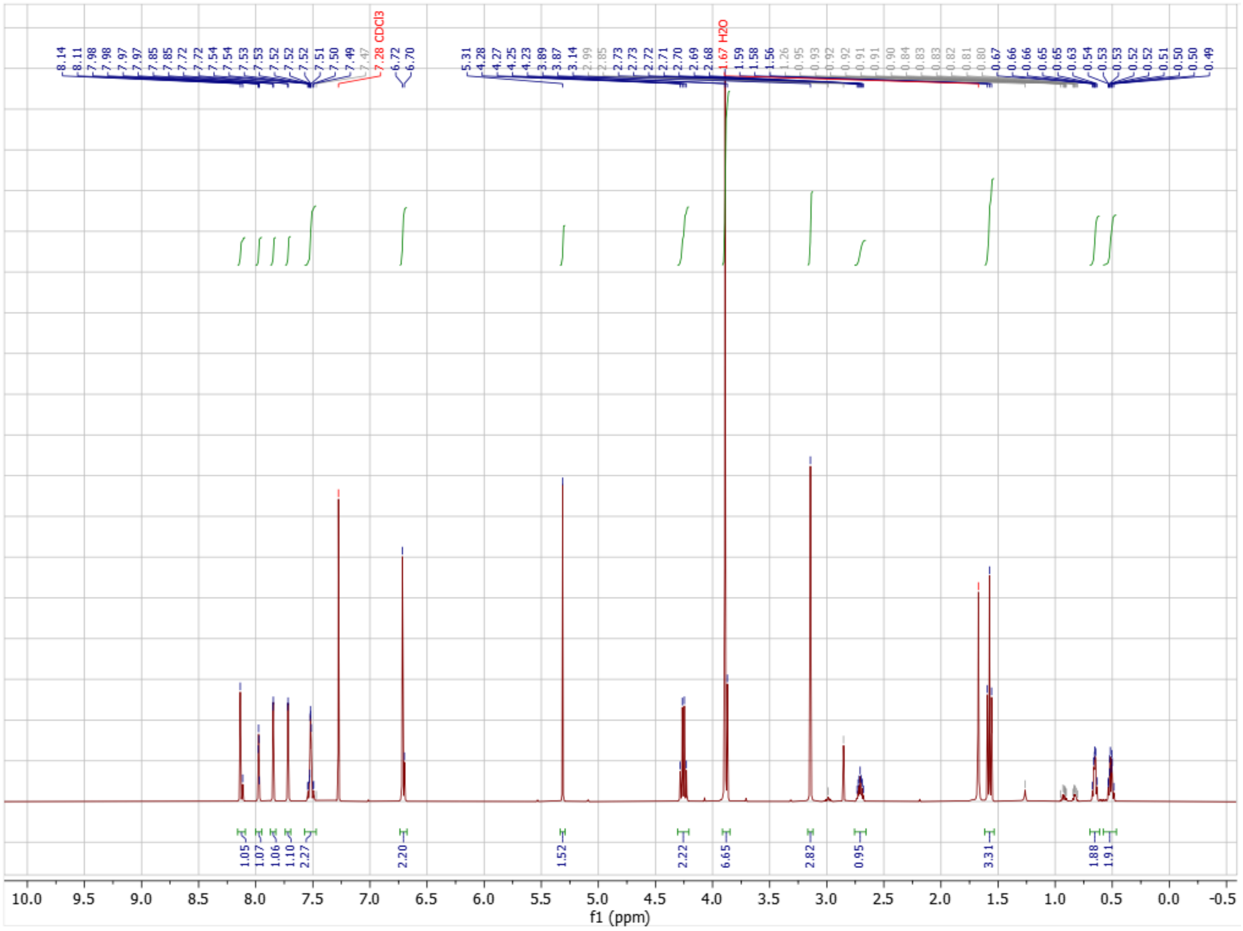

Compound **20**

Molecular Formula: $C_{23}H_{23}F_2N_5O_3$

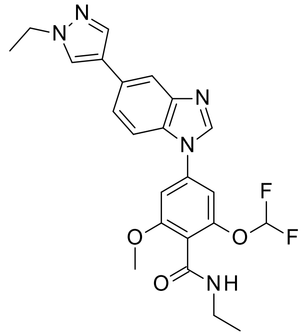

^{1}H NMR (400 MHz, Chloroform-*d*)

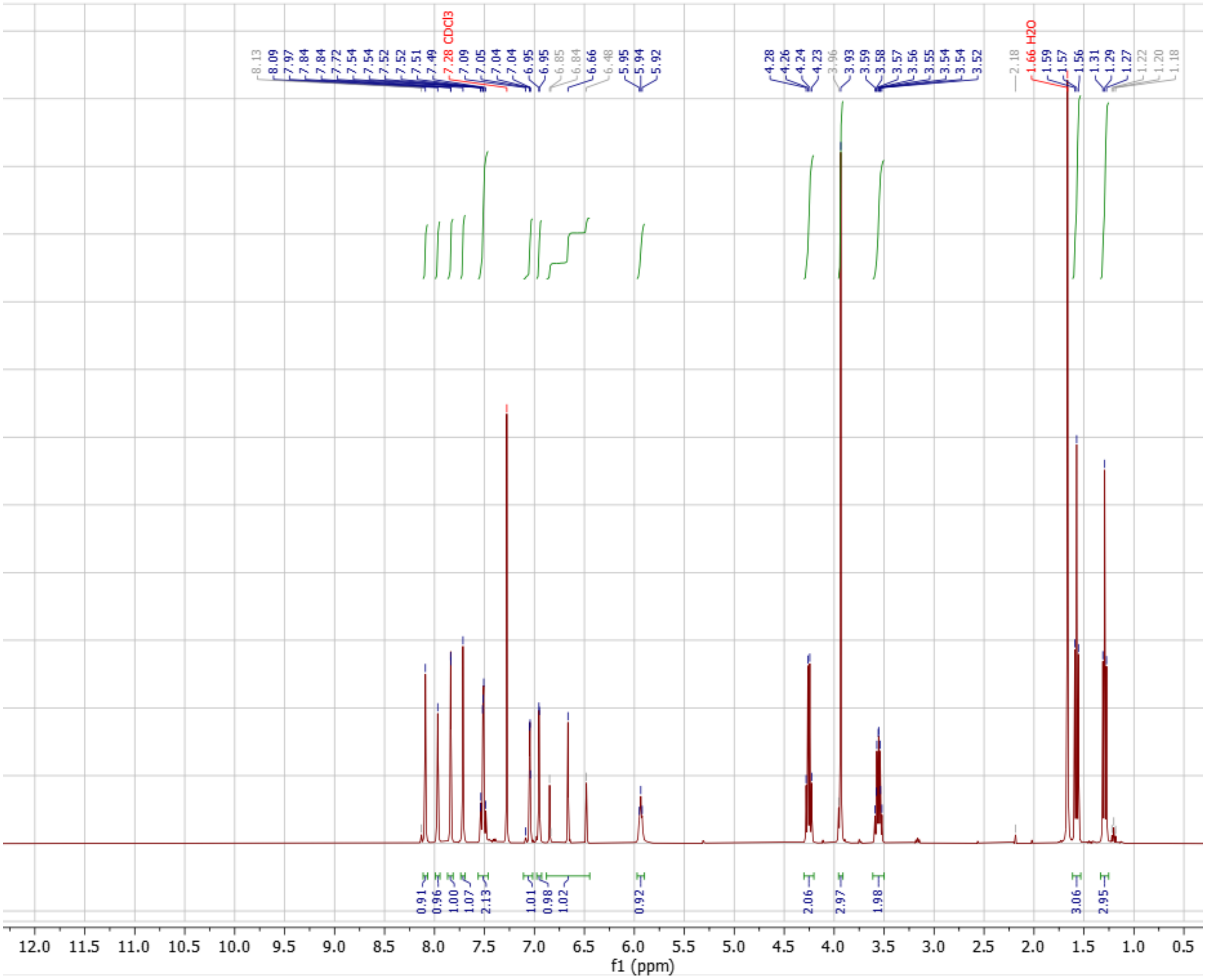

Compound **21**

Molecular Formula: $C_{22}H_{23}N_5O_3$

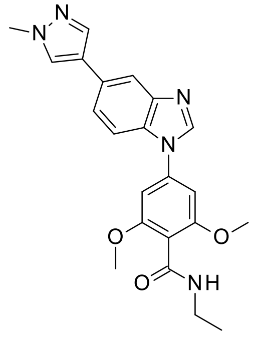

^{1}H NMR (400 MHz, DMSO-d_6)

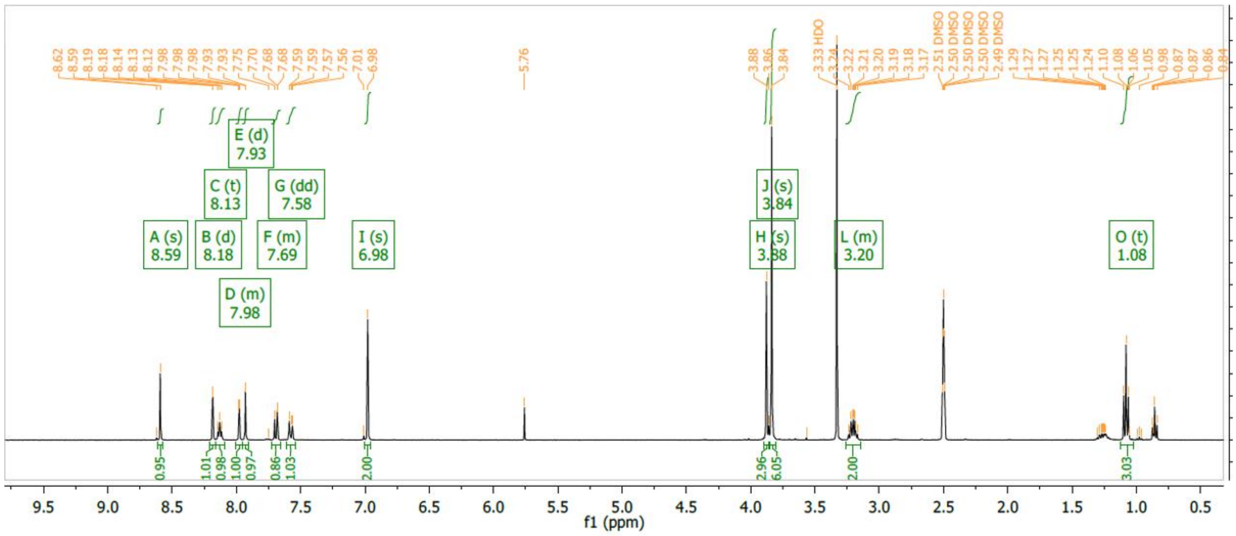

Compound **22**

Molecular Formula: $C_{23}H_{25}N_5O_4$

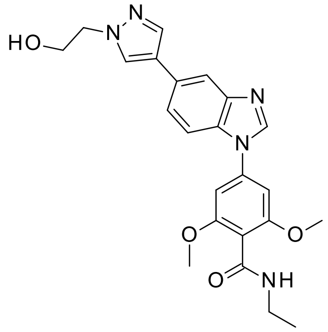

^{1}H NMR (400 MHz, Chloroform-*d*)

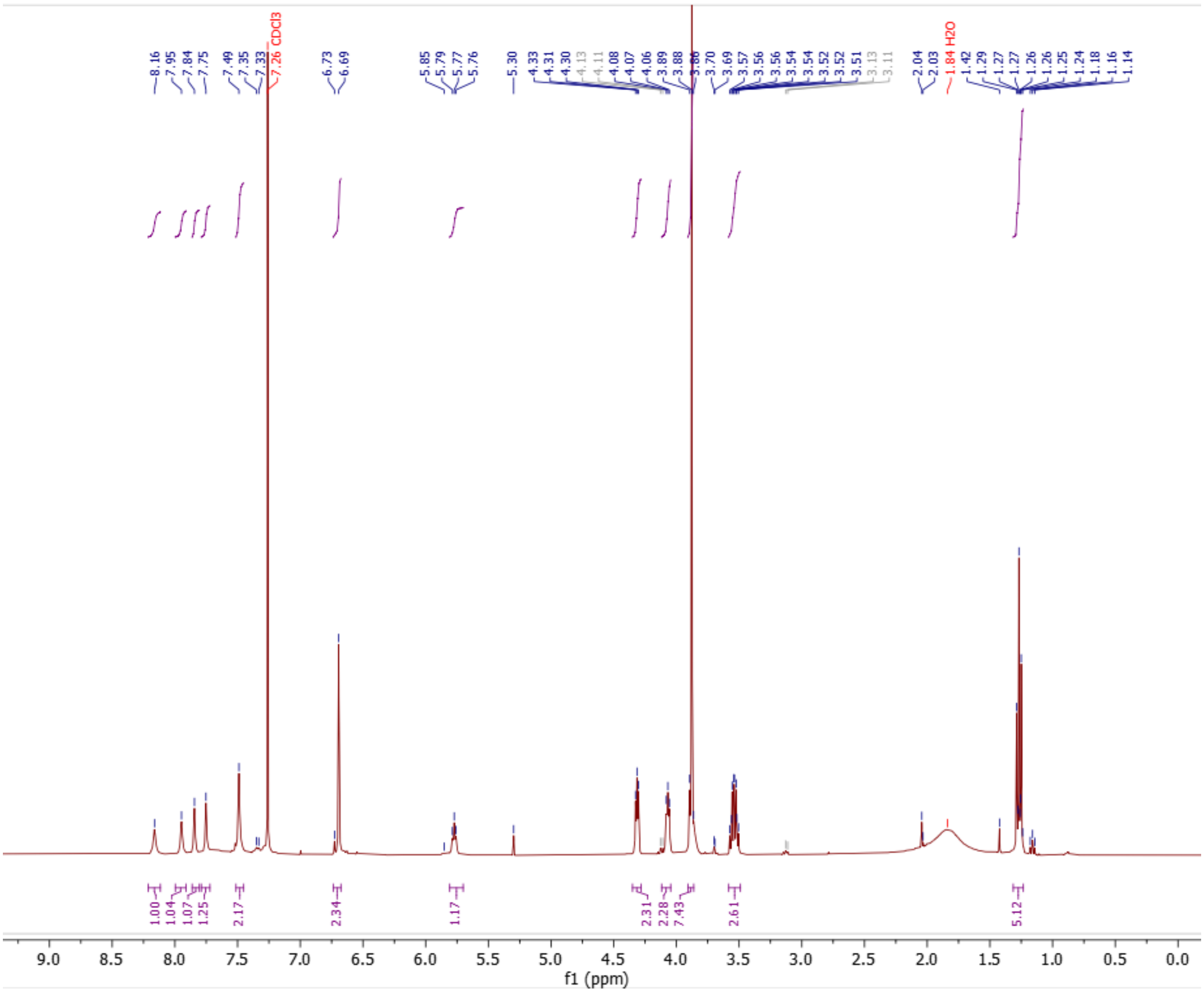

Compound **23**

Molecular Formula: $C_{23}H_{24}N_6O_4$

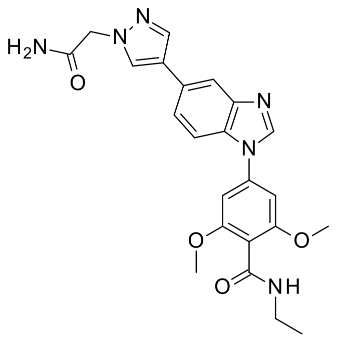

^{1}H NMR (400 MHz, Chloroform-*d*)

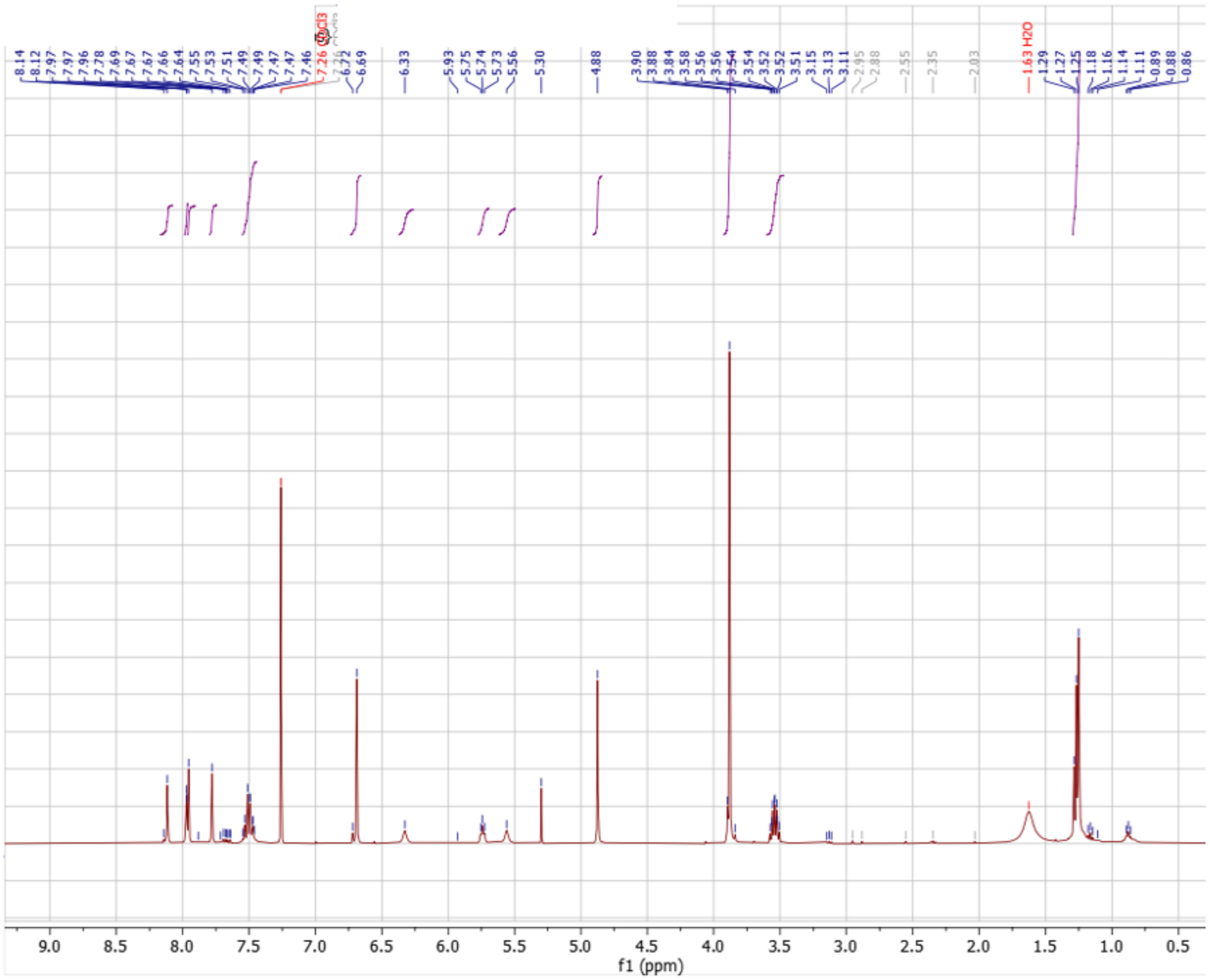

Compound **24**

Molecular Formula: $C_{23}H_{22}N_6O_3$

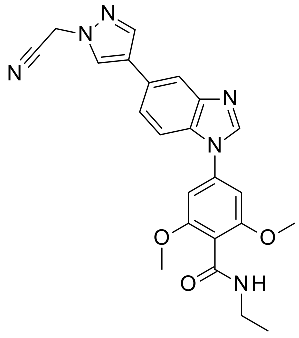

^{1}H NMR (400 MHz, Chloroform-*d*)

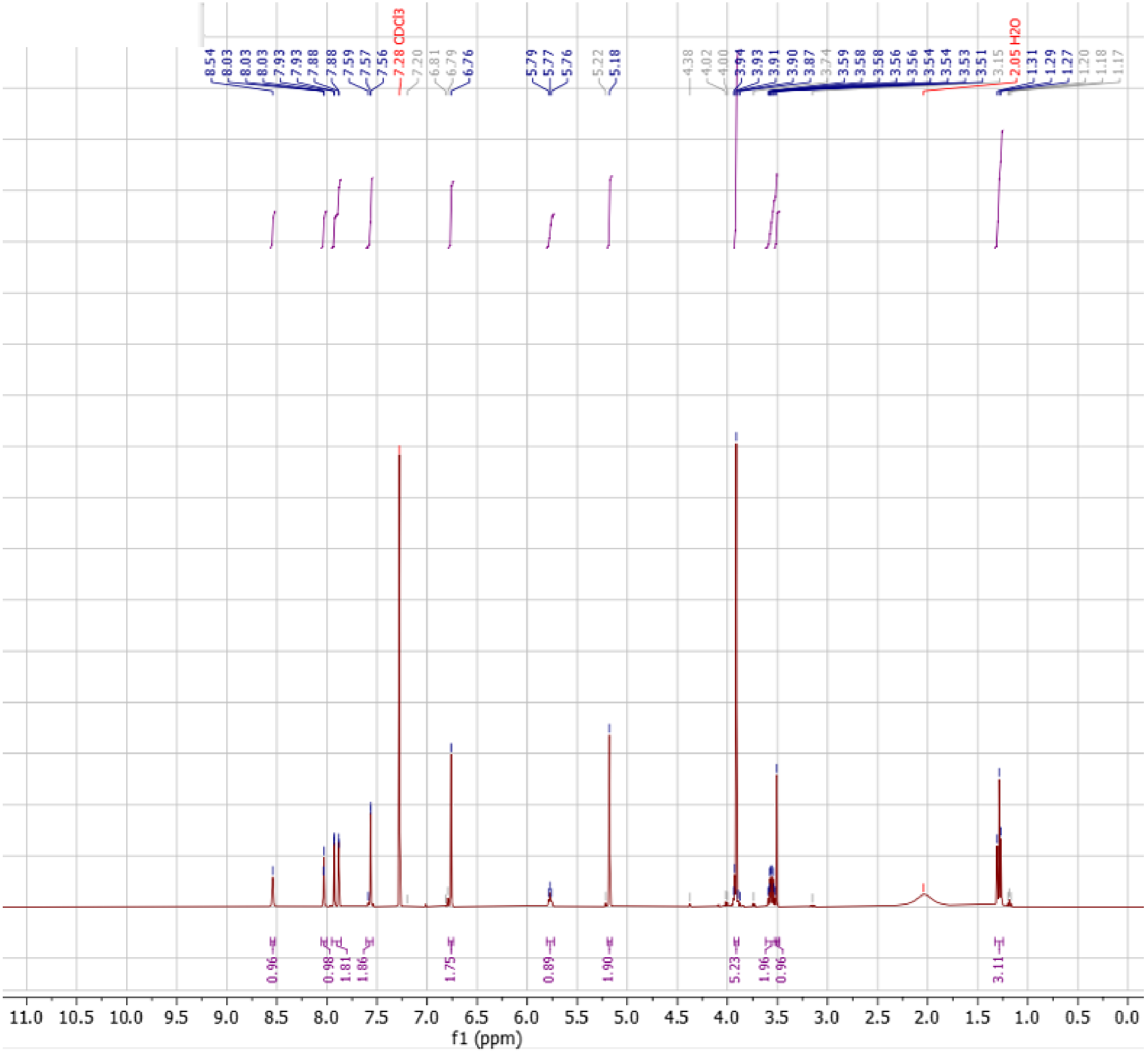

Compound **25**

Molecular Formula: $C_{24}H_{27}N_5O_4$

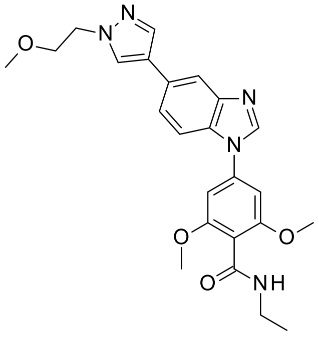

^{1}H NMR (400 MHz, Chloroform-*d*)

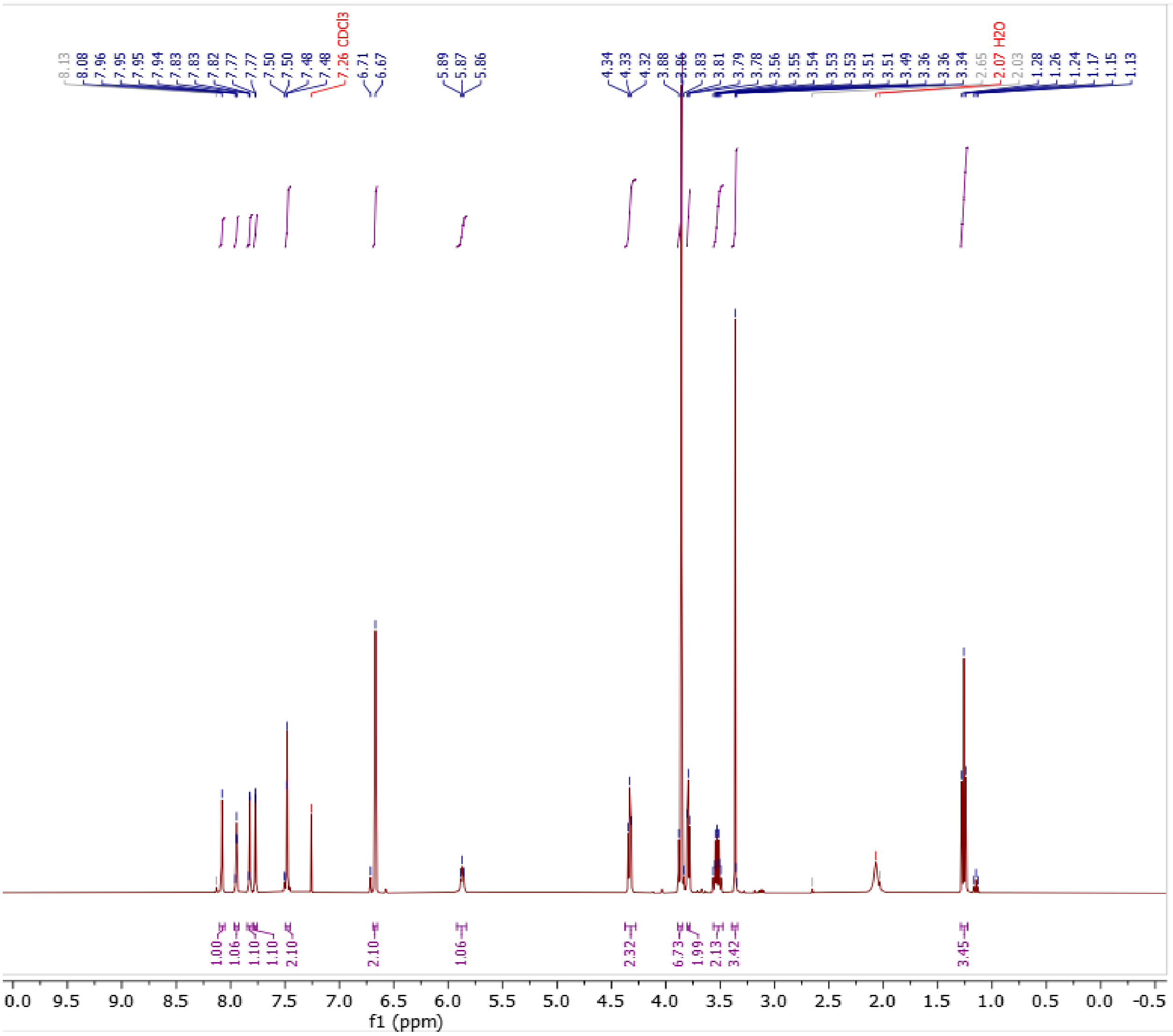

Compound **26**

Molecular Formula: $C_{26}H_{29}N_5O_4$

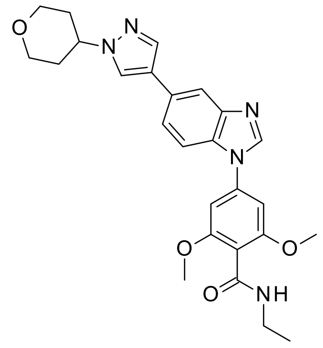

^{1}H NMR (400 MHz, Chloroform-*d*)

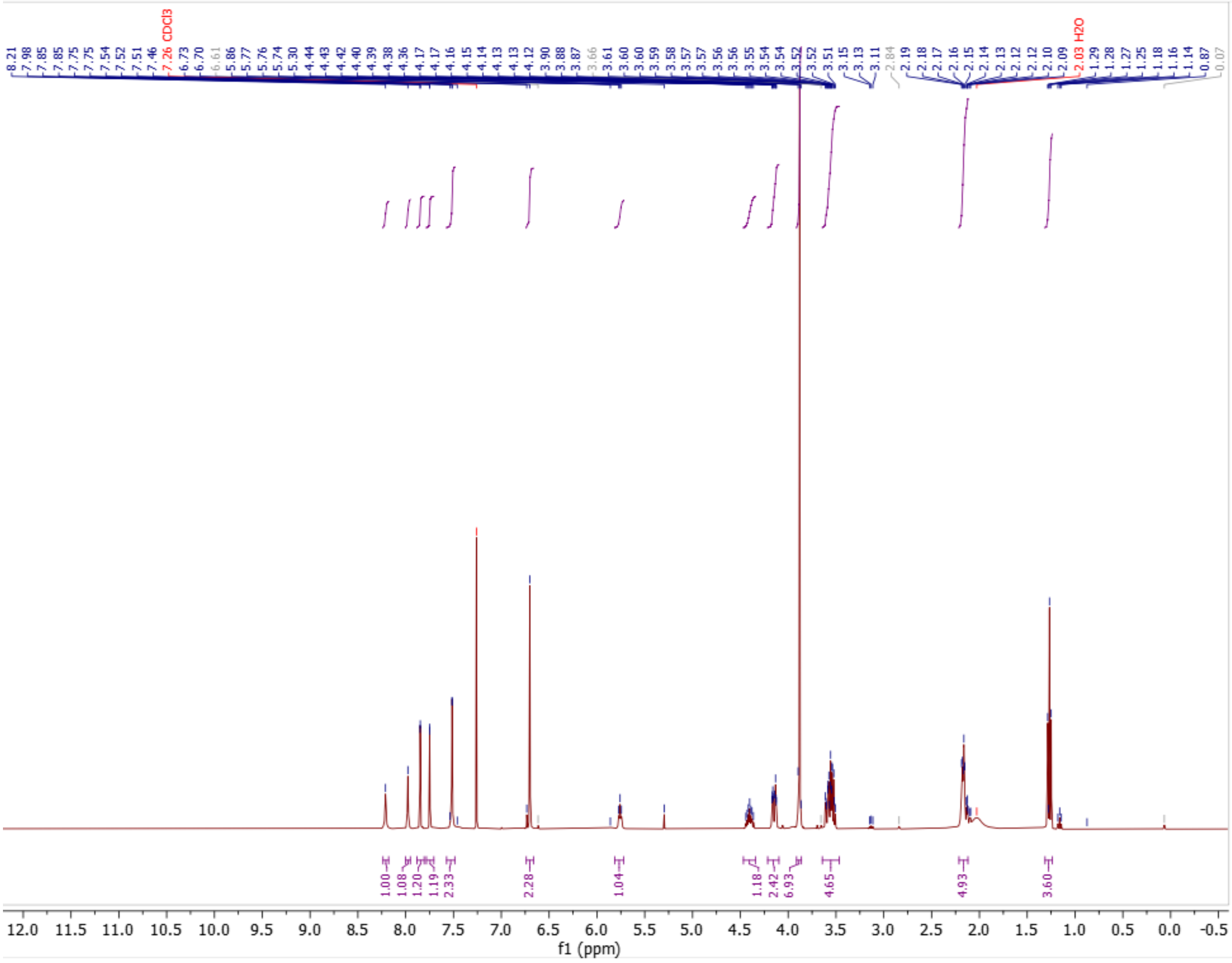

Compound **27**

Molecular Formula: $C_{22}H_{21}F_2N_5O_3$

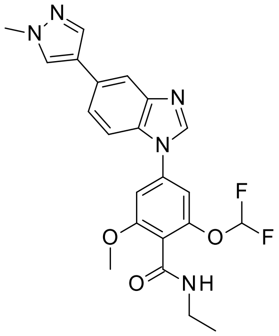

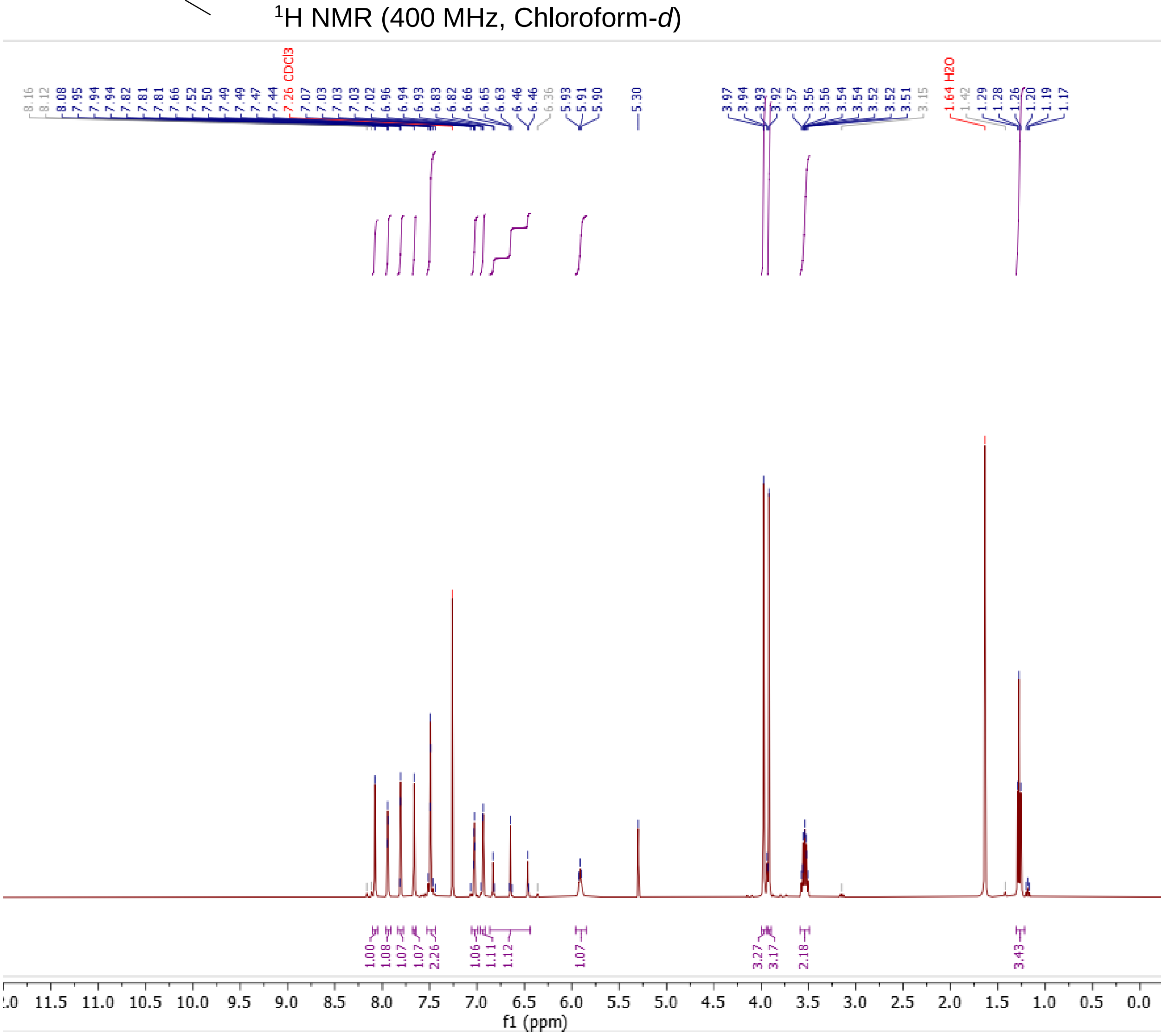

Compound **28**

Molecular Formula: $C_{23}H_{21}F_2N_5O_3$

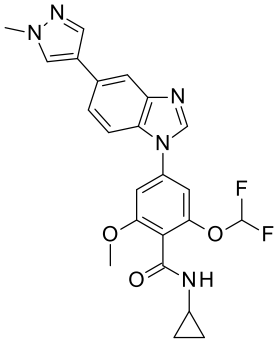

^{1}H NMR (400 MHz, Chloroform-*d*)

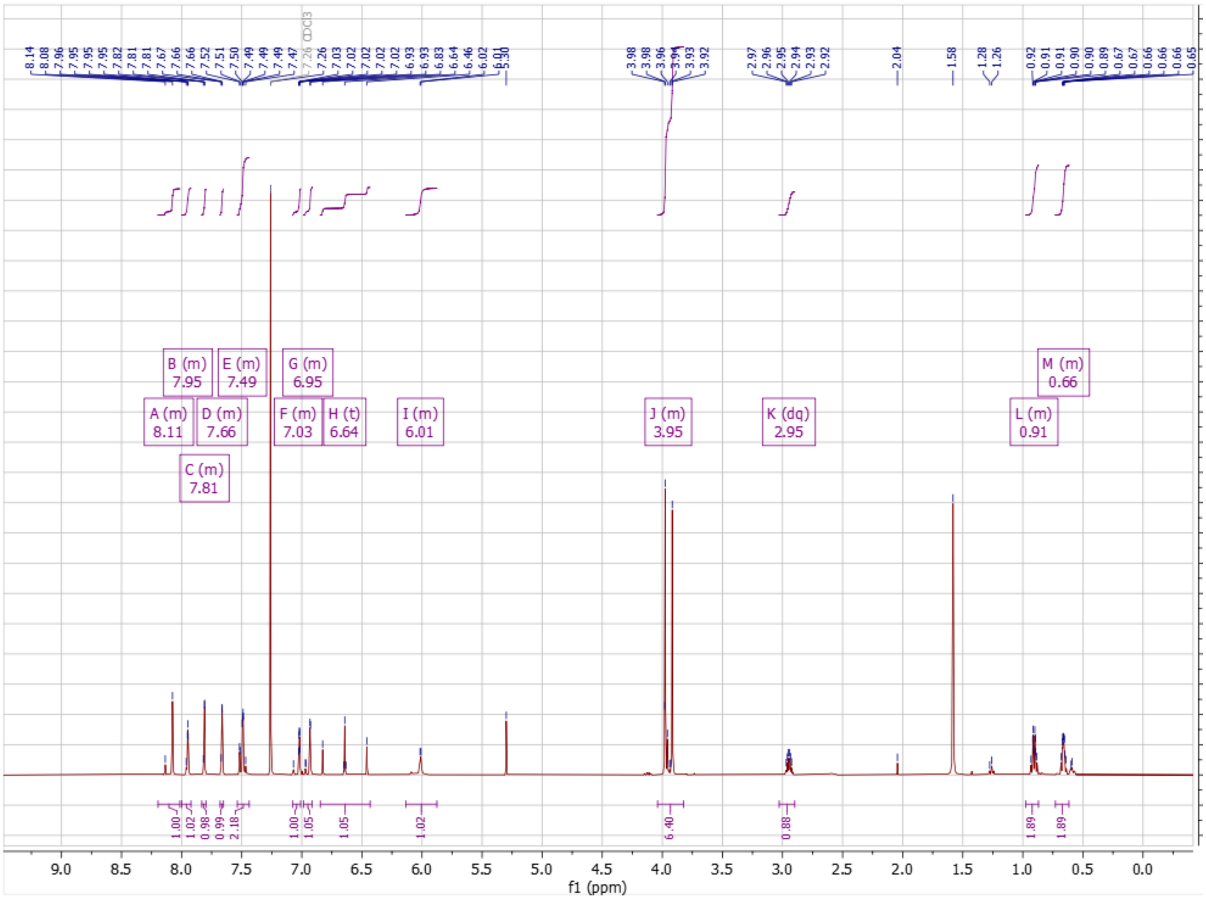

Supplement: Supplementary file 1 — jm3c01428_si_001.pdf [file jm3c01428_si_001.pdf]
